# An 8-week jump training did not boost effort value or the willingness to exert effort in a student sample

**DOI:** 10.1371/journal.pone.0352897

**Published:** 2026-07-16

**Authors:** Johanna Stähler, Maik Bieleke, Wanja Wolff, Markus Gruber, Ursula Fischer, Julia Schüler

**Affiliations:** 1 Department of Sport Science, University of Konstanz, Constance, Germany; 2 Dynamics of Human Performance Regulation Laboratory, Department of Movement Science, University of Hamburg, Hamburg, Germany; 3 Institute for Educational Support for Behaviour, Social-Emotional, and Psychomotor Development, University of Teacher Education in Special Needs, Zurich, Switzerland; Sheffield Hallam University, UNITED KINGDOM OF GREAT BRITAIN AND NORTHERN IRELAND

## Abstract

Physical inactivity remains highly prevalent, partly driven by the aversive nature of effort. However, effort can also be experienced as rewarding, which is associated with greater overall physical activity. Accordingly, the present study investigates whether regular physical exercise alters the self-reported value of physical effort, neural activation during exercise, and the willingness to exert effort. Sixty-two young adults were assigned to either an eight-week high-intensity jump training or a control group. Participants completed a two-task cycling ergometer exercise before and after the intervention. The first task assessed the value of effort at three pre-determined intensity levels, whereas the second task allowed participants to self-select the intensity to measure their willingness to exert effort. Participants’ perceived exertion and state value of physical effort were assessed, along with neural activation in the ventromedial prefrontal cortex and pre-supplementary motor area using functional near-infrared spectroscopy. Bayesian analyses provided evidence against main effects of condition, time, as well as their interaction, for self-reported value of physical effort, willingness to exert effort, and neural activity. This suggests that the value of effort may be relatively stable, as an eight-week training intervention did not alter the value of physical effort or the willingness to exert effort.

## Introduction

Insufficient physical activity is widespread among both adults and adolescents [1,2]. Despite the well-documented physical and mental health benefits of being active [1,2] and numerous global initiatives and interventions aimed at increasing activity levels [e.g., 3, 4], inactivity has remained high or even increased in high-income countries [1,2]. One possible explanation is that sport requires physical effort [5], which is inherently costly [6], often perceived as aversive [7], and therefore frequently avoided [8]. For example, people may choose elevators over stairs or devalue desirable outcomes (e.g., improved fitness) because of the effort required to attain them [9]. At the same time, individuals do exert physical effort when striving for valued outcomes [10]. Yet for many, the long-term health benefits do not sufficiently outweigh physical effort’s costs and aversiveness, resulting in insufficient activity levels [11].

At the same time, accumulating evidence indicates that physical effort can simultaneously be perceived as valuable – at least to some degree, for some people, and in some situations [6,10]. Contrary to the effort minimization theory [12], some people seem to seek out effortful activities for the mere sake of exerting effort [13], such as choosing a physically challenging hiking route just because they like the effort. This apparent preference might reflect a higher subjective valuation of physical effort. Recent studies indicate that higher Value of Physical Effort (VoPE) [14] is associated with a more positive effort experience during exertion [15], reduced boredom in physically effortful tasks [16], and greater overall physical activity [14]. Enhancing VoPE could therefore be a promising way to help people be more physically active [17], with potential applications in rehabilitation, programs for older adults, or interventions targeting sedentary lifestyles. If VoPE can be modified, it may offer a complementary pathway to traditional approaches.

Theoretical work suggests mechanisms by which reduced effort aversiveness can increase individuals’ tendency to mobilize effort over time [see 6]. One prominent example is Learned Industriousness, which proposes that repeatedly pairing effort with a reward reduces effort’s aversiveness and increases willingness to exert effort [18]. Notably, effort aversiveness can in principle be reduced via two mechanisms: by decreasing perceived effort costs and/or by increasing VoPE. However, previous theoretical and empirical work has primarily focused on reductions in effort costs [e.g., 19, 20], whereas potential increases in effort valuation have received considerably less attention [10]. Thus, it remains unclear whether repeated physical exercise can increase VoPE.

A further open question concerns the generalizability of potential learning effects. Near-transfer describes greater valuation of effort in the same or similar tasks, whereas far-transfer refers to generalization to different tasks or contexts (e.g., from playing volleyball to other ball games). Findings on both types are mixed [e.g., 20, 21], and some evidence suggests that VoPE may be relatively stable [21]. To date, only one study has examined this mechanism in the context of exercise, focusing exclusively on near-transfer effects. In Bernacer et al. [19], initially inactive participants completed a three-month low-intensity exercise program. After the program effort costs had less influence on effort-based decisions, accompanied by decreased effort-related activity in the anterior cingulate cortex (ACC) [19]. These findings suggest that regular exercise may reduce effort costs and alter neural responses to effort.

This interpretation should be considered in light of the relatively low training intensity employed in the intervention. This may be relevant because effort intensity could play an important role in how effort is evaluated. Given that higher intensities are often valued less [15] and perceived as more aversive [22], they present greater potential for effort to become less aversive through training. In addition, high-intensity exercise may engage neurophysiological mechanisms, such as increased activation of the endogenous opioid and endocannabinoid systems, which have been linked to affective responses and reward processing during intense exertion [23,24]. However, it remains unclear whether training at higher intensities yields stronger effects on effort valuation.

To better understand potential changes in VoPE, it is useful to consider how effort can be assessed at different levels. Effort can be examined in behavioral, subjective, and neurological terms. Research distinguishes between objective effort and subjectively perceived exertion [25]. Objective effort refers to the actual physical effort that can be assessed through physiological markers such as heart rate [26]. Subjective effort includes perceived intensity, typically measured using self-report instruments such as the Rating of Perceived Exertion (RPE) scale [27], as well as valence (positive/negative), assessed, for example, with the Value of Physical Effort (VoPE) Scale [14]. Neurophysiological research further indicates that different brain regions are associated with distinct aspects of effort processing. Activity in the pre-supplementary motor area (preSMA) serves as an indicator of effort intensity [28] and a transcranial magnetic stimulation study suggests that disrupting preSMA activity reduces perceived exertion [29], indicating a key role in effort perception. Regarding effort valence, the ACC primarily tracks decision difficulty [30] or effort costs [19], whereas the ventromedial prefrontal cortex (vmPFC) signals net value (i.e., reward after accounting for effort). Overall, meta-analytic evidence suggest that physical effort exertion and valuation are associated with distinct neural correlates, particularly preSMA and vmPFC [28].

The aim of the present study therefore was to examine whether regular physical exercise alters the value of physical effort during actual physical exertion. Specifically, we investigated whether changes in subjective, behavioral and neural indicators of effort valuation generalized beyond the training task.

## Methods

### Study overview

This study was part of the research project ProPELL and approved by the ethics committee of the University of Konstanz (ref. 31/2022). Participants provided written informed consent prior to participation. This study investigated whether regular physical effort alters individuals’ VoPE and neural responses to physical effort, or whether VoPE remains relatively stable over time. Participants in the training group (TG) completed a multi-week jump training protocol, and VoPE was assessed pre/post using two cycling tasks, allowing us to test far-transfer effects across task domains [18,20]. Subjective, neural, and behavioral measures of VoPE were collected [20]. We hypothesized that (a) state VoPE during exercise would increase in the TG, potentially varying by effort intensity, (b) vmPFC oxygenation during cycling would increase in the TG, with no changes in preSMA [28], and (c) voluntary exertion (power output and heart rate) and RPE would increase in the TG, but not in the control group (CG).

### Participants

Participants were recruited at the local universities between August 22, 2022, and August 11, 2023 (see S1 for inclusion/exclusion criteria). The final sample comprised *N* = 62 participants (52% female, 48% male) with a mean age of 22.8 years (*SD* = 3.1), randomly assigned to either a TG (*n* = 34) or a CG (*n* = 28). Complete fNIRS data were available for 59 participants (TG *n* = 32; CG *n* = 27). A sensitivity analysis using G*Power indicated that, for our 2x2 mixed design and given the actual sample size and 80% power, detectable effect sizes were *f* = 0.31 for between-subjects effects, and *f* = 0.18 for within-subject effects and interactions. Participants were compensated for their participation in the ProPELL project and additionally received 10€ per session for participating in this study.

### Design and procedure

A ten-week longitudinal design was used with pre- and post-measurements and an eight-week training period. The TG performed high-intensity interval jump training (3x/week, ~ 15 minutes), while CG maintained daily routines. Jump training is well-suited for student samples, efficiently improves fitness [e.g., 31], is simple, time-efficient, and can be performed independently. Pre- and post-testing included a standardized warm-up and two cycling tasks (~2h total). Cycling was chosen for assessment because it permits individualized workload adjustments, precise effort manipulation, and more reliable functional near-infrared spectroscopy (fNIRS) recording, which is not feasible during jumping.

#### Training intervention.

Training was adapted to participants’ fitness and comprised a warm-up, hoppings, countermovement jumps (CMJs), and high-intensity CMJs, with volume and intensity progressively increased [see [32] for the protocol]. On average, TG participants reached 92% of maximal heart rate and 92% adherence [32]. The training proved partly effective, yielding specific improvements in CMJ height [32], but failed to increase overall physical fitness (e.g., increase in VO_2peak_ (for details see [32])).

#### Cycling tasks.

The interval task (15 minutes) consisted of low-, moderate-, and vigorous-intensity bouts (order randomized across participants), each lasting three minutes and separated by two-minute recovery breaks. Workload was individualized using the anaerobic threshold (AT) and the respiratory compensation point (RCP) derived from a Cardiopulmonary Exercise Test (CPET) (performed 2–4 days before both measurement sessions) [33]. Intensities were set to 85% of AT (low); the mean of AT and RCP, calculated as (AT[W] + RCP [W])/ 2 (moderate), and 5% above RCP (vigorous) [33,34]. To ensure the intended intensity, participants maintained a cadence of 85–95 revolutions per minute (rpm), while resistance levels were determined by the Powerbike software [Powerbike; 35]. Participants received real-time visual feedback on cadence and target power. At the end of each interval, RPE and state VoPE were assessed. After a ten-minute recovery break, participants completed a free ride task (10 minutes), cycling a virtual route (Allensbach to Langenrain; southern Germany). They freely selected their intensity without a predefined goal. Every two minutes, RPE and state VoPE were assessed. Heart rate (interval and free ride task) and hemodynamic oxygenation in the vmPFC and preSMA (interval task) were continuously recorded.

### Materials

#### Self-report assessment.

In both tasks, single items were presented via an automated recording using NIRSStim (Version 4.0, NIRx Medizintechnik GmbH, Berlin, Germany, 2016). To optimize timing, the verbal prompts were condensed into brief cues (“Exertion?” for RPE rating, “Joy in exertion?” for state VoPE rating, see below), while full questions and response scales were displayed visually. Participants responded verbally.

Perceived exertion (RPE) was assessed with the question “How strong do you exert yourself right now?” using the Category Ratio 10 scale [27,36], ranging from 0 (“no effort at all”) to 10 (“maximal”) or 11 (“even more than max”) [37]. Test-retest reliability was ICC = 0.61 for low, ICC = 0.51 for moderate, and ICC = 0.54 for vigorous intensity.

State value of physical effort (state VoPE) was assessed with “How much do you like exerting yourself right now?” using a bipolar 10-point scale, ranging from −5 (“I don’t like it at all”), 0 (“neutral”), to +5 (“I like it very much”). Test-retest reliability for state VoPE was ICC = 0.74 for low, ICC = 0.75 for moderate, and ICC = 0.78 for vigorous intensity.

#### Cyclus 2 ergometer and powerbike software.

Cycling tasks were performed on a Cyclus 2 ergometer (RBM ElektronikAutomation GmbH, Leipzig) controlled via the Powerbike software [35]. Powerbike enabled predefining interval protocols (resistance and time), and simulated outdoor routes by integrating slope and body weight. For both tasks, a virtual mountain bike was used (MTB Modern 22 Spd; 26/36 and 40/11 gears).

#### Cardiorespiratory measurement.

ECG was measured using the Equivital EQ02 + LifeMonitor (EQ02 + ; Equivital; Cambridge, UK), integrated into a vest and synchronized with PowerLab (PowerLab 16/35; ADInstruments; Oxford, UK). Signals were sampled at 400 Hz and saved via LabChart (Version 8.1.22; ADInstruments, 2022).

#### Functional near-infrared spectroscopy.

**Fig 1:**
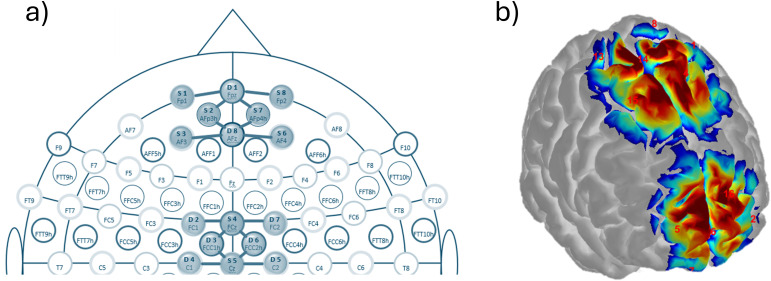
fNIRS montage (a) and sensitivity map of the utilized fNIRS montage (b). a) For the fNIRS measurement, sources (S) and detectors (D) were placed following the international 5/10 system: S1 at Fp1, S2 at AFp3h, S3 at AF3, S4 at FCz, S5 at Cz, S6 at AF4, S7 at AFp4h, S8 at Fp2, D1 at Fpz, D2 at FC1, D3 at FCC1h, D4 at C1, D5 at C2, D6 at FCC2h, D7 at FC2, D8 at AFz. This setup was designed to measure oxygenation in the ventromedial prefrontal cortex (vmPFC) and the pre-supplementary motor area (preSMA). b) The sensitivity map [Atlas Viewer, [39]] indicates a reasonably good capture of the vmPFC and preSMA with the used optode placement.

Oxygenation in the vmPFC and preSMA was measured using a multichannel continuous-wave fNIRS system (NIRSport, NIRx Medical Technologies LLC, NY, USA) and NIRStar software (Version 15.3, NIRx Medical Technologies LLC, NY, USA 2020) at 7.81 Hz (760/850 nm) with eight emitters and eight detectors forming 16 channels (25–35 mm). Optodes were arranged according to the international 5/10 system [38] (see Fig 1a) using a custom elastic NIRScap (NIRScap, EASYCAP GmbH, Herrsching, Germany) in multiple sizes and secured with an overcap (EASYCAP GmbH, Herrsching, Germany) to reduce motion artifacts and ambient light. Sensitivity was verified via Atlas Viewer simulations [39] (see Fig 1b) and optode performance was tested prior to each testing day by running a series of diagnostic procedures (e.g., gain modulation).

### Data Analysis

#### Electrocardiogram preprocessing.

ECG data were processed with Kubios HRV Scientific [version 4.1.0;[40]]. Artifacts were visually inspected and manually corrected [41,42]. Noisy segments were excluded from the analysis, and heart rate values were not computed for intervals with excessive noise. 88% of intervals were noise-free, 9% had minor artifacts and were corrected, 1% had significant noise, and 2% were excluded due to technical issues.

### fNIRS preprocessing

fNIRS data were preprocessed using HOMER3 [43] (MathWorks Inc., 2017b). First, the hmrR_pruneChannels Homer function excluded channels with too weak or too strong intensity (dRange: 1e-02, 3e + 00; SNRthresh: 2; SDrange: 0.0; 45.0). Negative intensity values were corrected using hmrR_PreprocessIntensity_Negative, and missing data were replaced by spline interpolation with hmrR_PreprocessIntensity_NAN. Optical intensity was converted to optical density using hmrR_Intensity2OD. Motion artifacts were addressed using a hybrid approach [44] employing methods that vary in strictness and methodological angle, enabling sensible noise removal without risking filtering out the neural signal altogether. Artifacts were first detected with hmrR_MotionArtifactByChannel (tMotion = 0.5, tMask = 1.5, STDEVthresh = 50.0, AMPthresh = 0.20) and corrected using hmrR_MotionCorrectSpline (p = 0.99), using relatively liberal parameters appropriate for the high-movement cycling task. Subsequently, hmrR_MotionCorrectWavelet was applied with a stricter-than-usual IQR (Iqr = 1.0 vs. typical ~1.5) to attenuate residual motion spikes, counterbalancing the preceding liberal settings. A bandpass filter (hmrR_BandpassFilt) with cutoff frequencies of 0.01 Hz (high-pass) and 0.5 Hz (low-pass) was applied to remove physiological noise, such as cardiac and respiratory artifacts. Subsequently, data were converted to changes in oxy- and deoxyhemoglobin concentration using hmrR_OD2Conc, which applies the modified Beer-Lambert law [45] with differential pathlength factors of 6.0 for both wavelengths. Finally, the hemodynamic response function (HRF) for relevant intervals (t = 60 sec for each interval) was calculated using hmrR_BlockAvg: Block_Average_on_Concentration_Data. Only the second minute of each interval was used, excluding the initial 60-second ramp-up phase, during which heart rate increases and only stabilizes over time [46], as well as the query period at the end of each interval, where participants gave verbal responses.

Overall, 6.3% of channels were rejected due to insufficient signal quality. Of these rejected channels, 29% were excluded automatically by the preprocessing stream, while the remaining 71% were identified and removed through manual inspection. (To assess the robustness of the findings with respect to preprocessing choices, the data were additionally processed using an alternative fNIRS preprocessing pipeline previously applied in physical effort paradigms [47]. This alternative approach yielded highly similar results and did not change the overall pattern of findings, indicating that the reported results were not driven by specific preprocessing parameter choices.)

#### Analyses.

Analyses were conducted in R [version 4.3.1, 48] and JASP [49]. Data, R-script, packages, and JASP files are available in OSF (https://osf.io/acp9d/overview?view_only=9b3a9ed691804ab78450486158fe7572). To test comparability of the three effort intensities across conditions and measurement points, heart rate and perceived exertion (RPE) were analyzed using a 2-between (condition: TG, CG) x 3-within (intensity levels: low, moderate, vigorous) x 2-within (time: pre- and post-training) mixed ANOVA in R.

Training effects on VoPE and neural responses were analyzed using Bayesian repeated-measures ANOVAs in JASP using default Cauchy (0, 0.5) priors for fixed effects [49]. This approach allows to quantify evidence both for the hypotheses and in favor of the null hypothesis [50]. Normal residuals with equal variance across groups were assumed. Bayes factors were estimated via 10,000 integration steps and posterior samples via 10,000 Markov Chain Monte Carlo (MCMC) iterations. Model comparisons were based on Bayes Factors (BF10/BF01) and posterior model probability as indicators of relative model adequacy. The complete output can be found in the JASP files on OSF.

For state VoPE and cerebral oxygenation (oxygenated (HbO) and de-oxygenated (HbR) hemoglobin concentration) of the interval task, mean values of the second minute of the ride were analyzed using the same 2-between (condition: TG, CG) x 3-within (intensity levels: low, moderate, vigorous) x 2-within (time: pre- and post-training) Bayesian repeated measures ANOVA design in JASP. For the free ride task, 2-between (condition: TG, CG) x 2-within (time: pre- and post-training) Bayesian repeated measures ANOVAs were conducted for heart rate (10-minute mean), RPE, rendered power (10-minute mean, normalized to the RCP), and state VoPE. All primary analyses were pre-specified, whereas post hoc analyses were considered exploratory and should be interpreted with appropriate caution given the number of comparisons.

In addition to Bayesian analyses, effect sizes were calculated to quantify the magnitude of observed effects. For ANOVA effects, *ω²* was reported (0.01 = small, 0.06 = medium, 0.14 = large) [51]. For pairwise comparisons, Cohen’s *d* was reported (0.2 = small, 0.5 = medium, 0.8 = large), along with corresponding 95% confidence intervals [51].

## Results

Table 1 displays the descriptive statistics of the interval task and the free ride task for both conditions and both measurement points. The three intensity intervals differ significantly in produced power (all *p* < .001) and in heart rate (all *p* < .001), with the vigorous interval eliciting the highest values, followed by the moderate and low intervals. These results indicate the successful manipulation of physical effort intensity (for detailed results see S2). The TG and CG did not differ significantly in heart rate (*p* = .733), rendered power (*p* = .068), or RPE (*p* = .711) across measurement time points (pre-/post-training), suggesting that the task was comparably demanding in both conditions (see S2). Furthermore, baseline comparability between conditions was examined. Although baseline differences were observed for two variables (see S3), subsequent analyses indicated that these differences could not be attributed to condition.

**Table 1:** Means and standard deviations by interval and free ride task, for the training (TG) and control (CG) groups at both measurement points.

Variable		Low interval		Moderate interval		Vigorous interval		Free ride task	
		TG	CG	TG	CG	TG	CG	TG	CG
**Power [W]**	M1	113.38 ± 29.62	102.79 ± 30.83	156.83 ± 37.17	143.10 ± 38.69	190.13 ± 45.22	174.72 ± 43.64	154.76 ± 45.89	145.61 ± 42.02
	M2	115.78 ± 31.59	97.03 ± 27.54	160.35 ± 37.59	139.96 ± 34.32	193.81 ± 43.16	175.38 ± 40.48	150.88 ± 49.91	140.66 ± 40.21
**Heart rate** **[min** ^**-1**^ **]**	M1	146.47 ± 15.38	150.32 ± 15.16	157.13 ± 13.34	159.39 ± 13.28	162.24 ± 12.07	164.89 ± 9.87	160.00 ± 17.14	162.72 ± 18.20
	M2	144.13 ± 13.89	143.39 ± 12.51	155.86 ± 12.18	152.91 ± 13.30	160.29 ± 12.17	159.83 ± 13.51	158.20 ± 15.90	157.92 ± 17.16
**RPE**	M1	3.12 ± 1.39	3.18 ± 1.36	4.18 ± 1.41	3.82 ± 1.12	5.38 ± 1.53	5.14 ± 1.26	4.10 ± 1.79	3.99 ± 1.47
	M2	2.85 ± 1.40	2.70 ± 1.08	3.75 ± 1.44	3.84 ± 1.43	5.16 ± 1.63	5.11 ± 2.01	3.65 ± 1.52	3.94 ± 1.63
**State VoPE**	M1	2.15 ± 1.73	1.50 ± 1.93	2.03 ± 2.05	1.36 ± 2.11	1.56 ± 1.80	1.00 ± 2.28	2.11 ± 1.76	1.21 ± 2.22
	M2	2.18 ± 1.29	1.18 ± 2.45	2.21 ± 1.59	1.18 ± 2.09	1.54 ± 1.94	0.82 ± 2.54	2.21 ± 1.52	1.13 ± 2.13
**preSMA HbO [µmol/l]**	M1	−0.09 ± 1.06	0.20 ± 1.58	−0.49 ± 0.76	0.27 ± 2.17	−0.54 ± 0.82	−0.38 ± 1.23	–	–
	M2	−0.12 ± 0.71	−0.21 ± 0.84	−0.33 ± 1.03	−0.08 ± 0.93	−0.55 ± 1.18	−0.77 ± 1.24	–	–
**preSMA HbR [µmol/l]**	M1	0.06 ± 0.54	0.34 ± 0.49	0.27 ± 0.52	0.41 ± 0.82	0.34 ± 0.48	0.61 ± 0.63	–	–
	M2	0.11 ± 0.41	0.10 ± 0.39	0.15 ± 0.43	0.28 ± 0.42	0.50 ± 0.53	0.61 ± 0.46	–	–
**vmPFC HbO [µmol/l]**	M1	1.27 ± 3.81	0.42 ± 3.13	1.09 ± 2.56	0.80 ± 2.46	0.59 ± 3.17	0.52 ± 2.85	–	–
	M2	0.50 ± 1.81	0.54 ± 2.40	1.42 ± 3.55	1.43 ± 2.79	0.72 ± 3.51	0.84 ± 3.19	–	–
**vmPFC HbR [µmol/l]**	M1	0.36 ± 1.04	0.29 ± 0.91	0.72 ± 1.12	0.70 ± 1.09	0.81 ± 1.00	0.75 ± 0.63	–	–
	M2	0.27 ± 0.45	0.25 ± 0.74	0.63 ± 1.03	0.63 ± 0.75	0.85 ± 0.97	0.86 ± 0.75	–	–

M1 = pre-training, M2 = post-training; RPE = Rating of Perceived Exertion, range 0–10; state VoPE = state Value of Physical Effort, range −5 - + 5; preSMA = pre-supplementary motor area; vmPFC = ventromedial prefrontal cortex; HbO = oxygenated hemoglobin; HbR = deoxygenated hemoglobin.

### Interval cycling task

#### State VoPE.

A Bayesian repeated measures ANOVA tested whether state VoPE would increase in the TG while remaining stable in the CG. The best model including the Time x Condition interaction, also included main effects of Time, Condition, and Interval, BF_10_ = 4.98, P(M/data) =.04, *ω²* = 0.017 (for complete results tables, see OSF), providing substantial evidence for the alternative hypothesis [52]. However, model-averaged inclusion probabilities indicated that only the main effect of interval should be retained, BF_incl_ = 16.60, P(incl/data) =.98. There was substantial evidence against the main effect of Time, BF_incl_ = 0.12, P(incl/data) =.25, and against the Time x Condition interaction, BF_incl_ = 0.13, P(incl/data) =.06. Evidence for the main effect of Condition was inconclusive, BF_incl_ = 0.51, P(incl/data) =.59.

**Fig 2:**
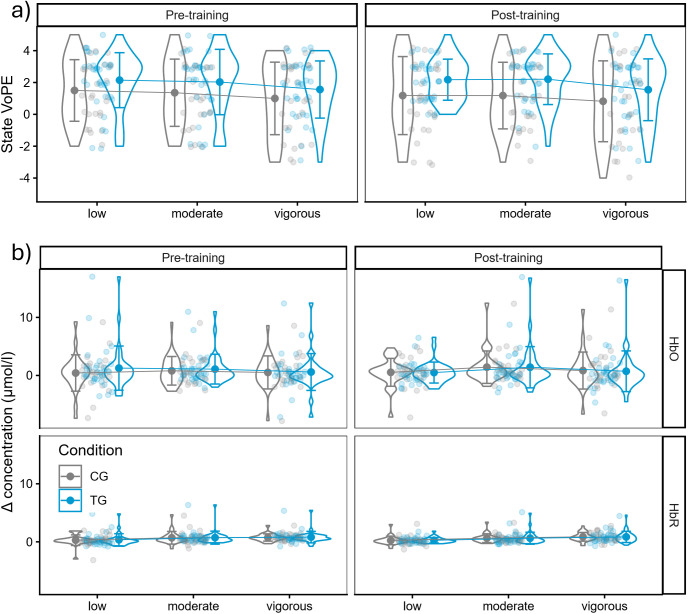
Violin plots with mean, standard deviation, and raw data points for the control and training groups for a) state value of physical effort and b) the average HbO and HbR concentrations in the vmPFC for each intensity level before and after the training phase. CG = control group, TG = training group. **a)** The main effect of Interval was strongly supported by the data. Post hoc comparisons indicated strong evidence that state VoPE was lower during the vigorous interval compared to the low and moderate intervals. b) vmPFC HbR: The main effect of Interval was strongly supported by the data, with lower concentration in the low than in the moderate and vigorous intervals.

The Interval effect was further supported by the second-highest Bayes Factor among candidate models, BF_10_ = 37.97, P(M/data) =.31, providing very strong evidence. Exploratory post hoc comparisons indicated strong evidence that state VoPE ratings were lower during the vigorous interval (*M* = 1.26, *SD* = 2.13) compared to the low (*M* = 1.79, *SD* = 1.89), BF_10_ = 167.43, Odds_post_ = 98.35, *d* = 0.26, 95% CI [0.04, 0.48], and moderate intervals, BF_10_ = 140.77, Odds_post_ = 82.69, *d* = 0.23, 95% CI [0.04, 0.43], with both differences corresponding to small effect sizes. Substantial evidence supported equivalent ratings between the low and moderate (*M* = 1.73, *SD* = 1.98) intervals, BF_10_ = 0.12, Odds_post_ = 0.07 (see Table 1 and Fig 2a; for complete results table, see OSF).

#### HbO concentration vmPFC.

A Bayesian repeated measures ANOVA tested whether the vmPFC HbO concentration would increase in the TG while remaining stable in the CG. The best model including the Time x Condition interaction, also included main effects of Time and Condition, BF_01_ = 37.19, P(M/data) =.01, providing very strong evidence for the null hypothesis [52] (see Fig 2b). Additionally, model-averaged inclusion probabilities indicated that the null model was substantially more likely than all competing models that included the predictors Interval, BF_excl_ = 9.81, P(excl/data) =.78, Time, BF_excl_ = 10.62, P(excl/data) =.79, Condition, BF_excl_ = 8.10, P(excl/data) =.74, or their interactions, BF_excl_ = 26.82, P(excl/data) =.74 (for complete results table, see OSF).

### HbR concentration vmPFC

A Bayesian repeated measures ANOVA tested whether the vmPFC HbR concentration would increase in the TG while remaining stable in the CG. The best model including the Time x Condition interaction, also included main effects of Time, Condition, and Interval, BF_10_ = 8441.33, P(M/data) =.23, *ω²* = 0.000, providing very strong evidence for the alternative hypothesis. However, model-averaged inclusion probabilities indicated that only the main effect of Interval should be retained in the model, BF_incl_ = 151530.18, P(incl/data) = 1.00. There was evidence against the other predictors, such as Time, BF_incl_ = 0.10, P(incl/data) =.22, Condition, BF_incl_ = 0.12, P(incl/data) =.25, and their Interactions, BF_incl_ = 0.03, P(incl/data) =.02 (for complete results table, see OSF).

The Interval effect was further supported by the highest Bayes Factor among all candidate models, BF_10_ = 401155.86, P(M/data) =.60, providing decisive evidence. Exploratory post hoc comparisons indicated strong evidence that the HbR concentration in the vmPFC was lower during the low interval compared to the moderate (BF_10_ = 223.31, Odds_post_ = 131.17, *d* = 0.41, 95% CI [0.10, 0.67], small effect) and vigorous intervals (BF_10_ = 7.23*10^+7^, Odds_post_ = 4.25*10^+7^, *d* = 0.58, 95% CI [0.33, 0.83], moderate effect). Evidence for a vmPFC HbR difference between the moderate and vigorous intervals was inconclusive, BF_10_ = 0.91, Odds_post_ = 0.54 (see Table 1 and Fig 2b).

#### HbO concentration preSMA.

A Bayesian repeated measures ANOVA tested whether the preSMA HbO concentration would be stable across conditions and differ between intervals. The best model including the main effect of Interval and the Time x Condition interaction, also included main effects of Time and Condition, BF_10_ = 12.93, P(M/data) =.04, *ω²* = 0.000, providing strong evidence [52]. However, model-averaged inclusion probabilities indicated that only the main effect of Interval should be retained, BF_incl_ = 56.14, P(incl/data) =.99. There was substantial evidence against including Time, BF_incl_ = 0.19, P(incl/data) =.34, and the Time x Condition interaction, BF_incl_ = 0.18, P(incl/data) =.08, while evidence against including Condition was inconclusive, BF_incl_ = 0.32, P(incl/data) =.47 (for complete results tables, see OSF).

The Interval effect was further supported by the highest Bayes Factor among all candidate models, BF_10_ = 120.25, P(M/data) =.37, providing decisive evidence. Exploratory post hoc comparisons indicated strong evidence that the preSMA HbO concentration was lower during the vigorous compared to the low (BF_10_ = 54483.89, Odds_post_ = 32003.89, *d* = 0.44, 95% CI [0.21, 0.67], small effect) and moderate intervals (BF_10_ = 10.50, Odds_post_ = 6.17, *d* = 0.35, 95% CI [0.02, 0.68], small effect). There was substantial evidence in favor of similar preSMA HbO concentrations between the low and moderate intervals (BF_10_ = 0.23, Odds_post_ = 0.14) (see Table 1 and S2 in the OSF).

#### HbR concentration preSMA.

A Bayesian repeated measures ANOVA tested whether the preSMA HbR concentrations would be stable across conditions and differ between intervals. The best model including the main effect of Interval and the Time x Condition interaction, also included main effects of Time, Condition, and the Time x Interval interaction, BF_10_ = 1993.84, P(M/data) =.03, *ω²* = 0.027, providing decisive evidence [52]. However, model-averaged inclusion probabilities indicated that only the main effect of Interval should be retained, BF_incl_ = 8615.71, P(incl/data) = 1.00. There was substantial evidence against including Time, BF_incl_ = 0.13, P(incl/data) =.27, and the Time x Condition interaction, BF_incl_ = 0.12, P(incl/data) =.05, whereas evidence regarding Condition was inconclusive, BF_incl_ = 0.54, P(incl/data) =.60 (for complete results tables, see OSF).

The Interval effect was also supported by the second-highest Bayes Factor among all candidate models, BF_10_ = 19943.26, P(M/data) =.31, providing decisive evidence. Exploratory post hoc comparisons indicated strong evidence for higher preSMA HbR concentrations during the vigorous compared to the low (BF_10_ = 134766.05, Odds_post_ = 79161.72, *d* = 0.70, 95% CI [0.31, 1.09], moderate-to-large effect) and moderate intervals (BF_10_ = 90.32, Oddspost = 53.05, *d* = 0.45, 95% CI [0.12, 0.78], small-to-moderate effect). Evidence was inconclusive about the difference between the moderate and the low interval, BF_10_ = 2.01, Odds_post_ = 1.29 (see Table 1).

### Free ride cycling task

#### Heart rate.

**Fig 3:**
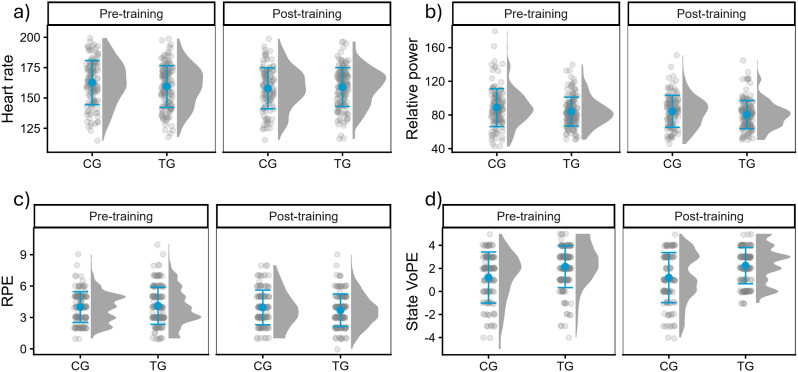
Raincloud plots for a) heart rate, b) relative power, c) perceived exertion, and d) state value of physical effort for both conditions and measurement points. In the raincloud plots, the mean values are shown with standard deviation instead of the typically displayed box plots. CG = control group, TG = training group. **a)** Heart rate in min-1. **b)** Relative power in percent of the threshold value of the RCP. **c)** RPE = Rating of Perceived Exertion. d) state VoPE = state Value of Physical Effort. The main effect of Condition was substantially supported by the data (BF_01_ = 0.33), with higher state VoPE in the TG than the CG.

A Bayesian repeated measures ANOVA tested whether heart rate would increase in the TG while remaining stable in the CG. The best model including the Time x Condition interaction, also included main effects of Time and Condition, BF_01_ = 14.66, P(M/data) =.03 (for complete results tables, see OSF), providing strong evidence for the null hypothesis [52]. Model-averaged results indicated substantial evidence against including the Time x Condition interaction, BF_excl_ = 8.05, P(excl/data) =.97, and the main effect of Condition, BF_excl_ = 3.60, P(excl/data) =.71, whereas evidence for Time was inconclusive, BF_excl_ = 2.31, P(excl/data) =.61 (see Fig 3a).

#### Relative power.

A Bayesian repeated measures ANOVA tested whether relative power would increase in the TG while remaining stable in the CG. The best model including Time x Condition interaction, also included main effects of Time and Condition, BF_01_ = 15.46, P(M/data) =.03, providing strong evidence for the null hypothesis [52] (for complete results tables, see OSF). Model-averaged results indicated substantial evidence against including the Time x Condition interaction, BF_excl_ = 8.63, P(excl/data) =.97, whereas evidence for Time, BF_excl_ = 2.89, P(excl/data) =.66, and Condition, BF_excl_ = 2.66, P(excl/data) =.64, was inconclusive (see Fig 3b).

#### Perceived exertion.

A Bayesian repeated measures ANOVA tested whether RPE would increase in the TG while remaining stable in the CG. The best model including the Time x Condition interaction, also included main effects of Time and Condition, BF_01_ = 4.75, P(M/data) =.07, providing substantial evidence for the null hypothesis [52] (for complete results tables, see OSF). Model-averaged results indicated that evidence for Time was inconclusive, BF_excl_ = 1.17, P(excl/data) =.44. There was substantial evidence against the inclusion of Condition, BF_excl_ = 3.43, P(excl/data) =.70, and the Time x Condition interaction, BF_excl_ = 3.39, P(excl/data) =.93 (see Fig 3c).

### State VoPE

Bayesian repeated measures ANOVA tested whether state VoPE would increase in the TG while remaining stable in the CG. The best model including the Time x Condition interaction, also included main effects of Time and Condition, BF_01_ = 6.15, P(M/data) =.03, providing substantial evidence for the null hypothesis [52] (for complete results tables, see OSF). Model-averaged results indicated evidence against including the main effect of Time, BF_excl_ = 6.27, P(excl./data) =.81, and the Time x Condition interaction, BF_excl_ = 7.50, P(excl./data) =.97 (see Fig 3d). The main effect for Condition, BF_excl_ = 0.48, P(excl/data) =.24, *ω²* = 0.039, was anecdotally supported. Exploratory post hoc comparisons for Condition further indicated substantial evidence that state VoPE was higher in the TG (*M* = 2.16, *SD* = 1.64) than the CG (*M* = 1.17, *SD* = 2.17) (BF_01_ = 0.05, Odds_post_ = 0.05, *d* = 0.55, 95% CI [0.19, 0.92], moderate effect).

To account for baseline differences in VoPE, we conducted a Bayesian ANCOVA with baseline VoPE as covariate. Model comparison favored the model including only baseline VoPE, BF_01_ = 6.37x10^-11^, (P(M/data) =.64), over the model including baseline and Condition, BF_01_ = 0.316, P(M/data = .36). Effect inclusion revealed evidence for excluding Condition as additional predictor, BF_excl_ = 1.74, P(excl/data) = 0.635, indicating that Condition did not explain variance beyond baseline VoPE.

## Discussion

This study investigated whether regular exposure to physical effort through high-intensity exercise alters individuals’ value of physical effort (VoPE) and neural responses to physical effort. The design offers several strengths, including high ecological validity [19], the successful implementation of three effort intensities, and a rigorous multi-method approach to capture different facets of effort valuation. Contrary to our hypotheses, eight weeks of jump training did not yield reliable evidence for changes in state VoPE, vmPFC responses during exertion, or voluntary exerted effort in a transfer task. These findings suggest that VoPE may be less readily modified than anticipated. Interestingly, although the TG reported generally higher state VoPE than the CG in the free ride task, ANCOVA controlling for baseline VoPE indicated that this difference was independent of the training. This reinforces the notion that VoPE may be relatively stable, particularly when far-transfer is tested.

Importantly, evidence for transfer effects in the domain of physical effort is considerably more limited and less consistent than in cognitive effort research, with learning effects often appearing to be task- and context-specific [53]. Although previous studies have demonstrated both near- and far-transfer effects, like reduced perceived physical effort costs and increased industriousness, in both animals [54] and humans [19], the conditions under which such transfer effects occur remain poorly understood.

Several characteristics of the intervention provide useful starting points for explaining the absence of learning-related changes. First, the training protocol was considerably more intense than in earlier studies [19,54]. While we initially assumed that high-intensity training would provide a strong learning signal, lower-intensity training may offer a more favorable context for effort-based learning. Our findings suggest that the aversive nature of intense effort [22] could attenuate the development of positive effort valuation. Importantly, the effectiveness of a given training intensity may depend on individual differences such as preference and tolerance to physical effort [55], cardiorespiratory fitness, or prior levels of physical activity. Future research should therefore systematically examine how different intensities interact with individual characteristics to shape VoPE learning, and to identify which individuals benefit most from specific training intensities.

Second, despite the high intensity of the training protocol, its overall training effect was limited [32]. Although jump performance improved, general fitness did not increase, suggesting that the training might not have been sufficiently effective to induce broader changes affecting higher-order motivational processes such as VoPE.

Moreover, according to the theory of Learned Industriousness [18], effort must be paired with rewards to reduce its subjective costs and potentially enhance its value. Such reinforcement can be inherent to the task itself or provided externally. In the context of physical training, inherent rewards may include improvements in physical fitness, weight loss, or body toning. Previous studies reporting decreased perceived effort costs and increased industriousness without explicit external reinforcement [19,54] may therefore have benefited from such inherent rewards. Based on these findings, and to avoid interference with other aspects of the research project, we refrained from implementing external rewards, expecting the training itself to be sufficiently rewarding. However, our findings suggest that this may not have held true. Participants exerted substantial effort, yet experienced little objectively noticeable improvements [32], which may have weakened reinforcement and limited increases in VoPE to a transfer task. Importantly, participants’ perception of progress or sense of competence were not assessed, which might have varied between participants even in the absence of measurable gains. This represents a methodological limitation that constrains interpretations regarding whether reinforcement via inherent rewards contributed to learning-related mechanisms. Interestingly, we did not observe a decrease in VoPE either, indicating that unrewarded effort neither enhances nor diminishes VoPE in a transfer task. Although previous studies also lacked explicit external rewards [19,54], the considerably higher effort intensity of our training protocol might have contributed to the different results. Given that higher efforts are typically associated with more negative affect [22] and thus greater inherent costs, the lack of adequate reinforcement following such strenuous effort may have further limited learning-related changes in VoPE. However, evidence from the cognitive domain is mixed regarding whether secondary reinforcement alone can effectively alter effort valuation [20,21]. To test whether consistent pairing of effort and reward is necessary for VoPE learning, future studies could implement structured reinforcement strategies, such as increasing awareness of progress through fitness assessments, explicit feedback, or by providing rewards proportionally to exertion rather than outcomes. This would offer a more rigorous test of Learned Industriousness in the domain of physical effort.

Another factor may lie in the timing of state VoPE assessment. Theories on effort suggest that individuals are more likely to value effort retrospectively once it has been completed, rather than during the effort [6,17]. Effort might be processed differently before, during, and after an activity [17]. In particular, during physical exertion, individuals are often focused on performance optimization and minimizing discomfort [12,17]. In contrast, retrospective evaluations may allow for a more reflective and positively biased appraisal of the experience [17]. Notably, these conceptual distinctions were articulated more clearly in the literature only after data collection for the present study had been completed, whereas the theoretical landscape at the time of study planning was less differentiated. Accordingly, VoPE was assessed concurrently during active exertion in a separate laboratory task, and participants were not asked to retrospectively evaluate the effort involved in the training itself. Future studies could benefit from assessing VoPE at multiple times to more accurately capture the temporal dynamics of effort valuation and potential learning effects [17].

Another possible explanation lies in the developmental sensitivity of learning VoPE [10]. Adolescents appear to be less sensitive to effort costs and exert more effort relative to task demands and rewards at stake than adults [56]. This period of heightened responsiveness may facilitate the learning of the relationship between effort and reward, making it easier to internalize the contingency that greater effort leads to greater rewards. By contrast, by the time individuals reach early adulthood, as in the current sample, it may be more challenging to relearn or modify their VoPE.

While this observation opens an interesting avenue for developmental research, it is also important to consider potential moderating factors such as previous learning experiences in the sport context, or genetic predispositions (e.g., differences in the dopaminergic reward system) that may influence how VoPE is learned. The present study was designed to test whether VoPE can be modified through regular physical exercise. Consequently, potential moderation or subgroup effects were not systematically examined. Moreover, given the modest sample size, the study was not sufficiently powered to reliably detect moderation effects. Future studies with lager sample sizes should therefore test similar interventions in adolescent samples and systematically examine individual differences that may shape sensitivity to learning VoPE. In addition, it may be informative to consider further variables such as physiological indicators (e.g., VO_2_max), prior physical activity levels, and individual differences in effort tolerance as potential additional moderators.

Finally, the current study assessed neural activation during periods of (partially) intense physical exertion, which offers valuable ecological validity. However, although fNIRS is relatively robust to motion artifacts [57], it is important to note that motion-related noise cannot be fully excluded, particularly in the context of physical demanding tasks such as a cycling task. Despite careful preprocessing and quality control procedures, residual motion artifacts may have influenced the signal and could have contributed to the observed null findings. Importantly, however, no corresponding effects were observed at the behavioral or subjective level, suggesting that the absence of neural differences is consistent across multiple levels of analysis and not solely attributable to measurement noise.

Furthermore, physical exercise involves multiple concurrent cognitive processes [58], which may obscure neural mechanisms specific to VoPE. Moreover, interpreting activation in specific brain regions necessarily relies on simplified heuristic functional attributions, which entails the risk of oversimplification and reverse inference. While this approach was useful for addressing the present research question at the current stage of the literature, it may have constrained our ability to draw clearer inferences, as network neuroscience emphasizes that functions likely emerge from interactions rather than one-to-one mappings between regions and processes.

Future research could expand on this by using whole brain functional analyses, examining neural activation during physical effort-related decision-making [19] or by employing more localized physical effort paradigms, such as grip force tasks, rather than whole-body exercises like cycling. Although such paradigms offer lower ecological validity, they would help disentangle distinct cognitive components and additionally allow the use of neuroimaging techniques like fMRI. In addition, the limitation of fNIRS to cortical surface activity [59] could be addressed in future studies by using fMRI to investigate subcortical regions known to be involved in effort and reward processing such as the amygdala [60], nucleus accumbens [61], and ventral striatum [62].

Taken together, our findings suggest that VoPE may be more stable and less malleable through training than previously assumed [e.g., [18][21]]. Nevertheless, limitations of the study design may have prevented capturing potential changes in VoPE, as suggested by previous research in both animals [54] and humans [19], as well as findings from the domain of cognitive effort [20]. Rather than providing a conclusive answer, our results open multiple avenues for future research to better understand the conditions under which VoPE potentially can be modified. Such efforts could help clarify whether VoPE is indeed systematically modifiable through training, or whether it represents a more stable, trait-like construct that resists modification under certain conditions.

## Conclusion

In summary, this study provides initial insights into whether regular physical exercise can influence the valuation of physical effort during exercise across behavioral, subjective, and neural levels. Contrary to our expectations, eight weeks of high-intensity jump training did not alter participants’ willingness to exert effort, their self-reported value of physical effort (VoPE), or neural responses in the vmPFC associated with effort valuation. Together, these findings suggest a relative stability of concurrent effort valuation in the context of the present training paradigm. Future research should therefore examine whether different training characteristics (e.g., training intensity), timing of VoPE assessment, the role of intrinsic and extrinsic rewards, or other populations (e.g., active individuals, adolescents) influence the modifiability of effort valuation.

### 5.1 Use of Generative AI

During the preparation of this work, the authors used ChatGPT to improve the readability and language of the manuscript. After using this tool, the authors reviewed and edited the content as needed and take full responsibility for the content of the published article.

## References

[1] Guthold R, Stevens GA, Riley LM, Bull FC. Global trends in insufficient physical activity among adolescents: a pooled analysis of 298 population-based surveys with 1·6 million participants. Lancet Child Adolesc Health. 2020;4(1):23–35. doi: 10.1016/S2352-4642(19)30323-2 31761562 PMC6919336

[2] Guthold R, Stevens GA, Riley LM, Bull FC. Worldwide trends in insufficient physical activity from 2001 to 2016: a pooled analysis of 358 population-based surveys with 1·9 million participants. Lancet Glob Health. 2018;6(10):e1077–86. doi: 10.1016/S2214-109X(18)30357-7 30193830

[3] WOOP my life. https://woopmylife.org/en/home. Accessed 2024 July 25.

[4] World Health Organization. Global action plan on physical activity 2018–2030: more active people for a healthier world. Geneva: World Health Organization. 2018. https://iris.who.int/handle/10665/272722

[5] Sport. Cambridge Dictionary. https://dictionary.cambridge.org/dictionary/english/sport. Accessed 2023 October 1.

[6] Inzlicht M, Shenhav A, Olivola CY. The Effort Paradox - Effort Is Both Costly and Valued. Trends in Cognitive Sciences. 2018;22(4):337–49. doi: 10.1016/j.tics.2018.01.007 29477776 PMC6172040

[7] Kurzban R. The sense of effort. Current Opinion in Psychology. 2016;7:67–70. doi: 10.1016/j.copsyc.2015.08.003

[8] Kool W, McGuire JT, Rosen ZB, Botvinick MM. Decision making and the avoidance of cognitive demand. J Exp Psychol Gen. 2010;139(4):665–82. doi: 10.1037/a0020198 20853993 PMC2970648

[9] Chong TT-J, Apps M, Giehl K, Sillence A, Grima LL, Husain M. Neurocomputational mechanisms underlying subjective valuation of effort costs. PLoS Biol. 2017;15(2):e1002598. doi: 10.1371/journal.pbio.1002598 28234892 PMC5325181

[10] Stähler J, Bieleke M, Wolff W, Schüler J. Different functions of physical effort in physical activity and sports: a scoping review of the value of physical effort. Motiv Emot. 2025. doi: 10.1007/s11031-025-10123-3

[11] Maltagliati S, Sarrazin P, Fessler L, Lebreton M, Cheval B. Why people should run after positive affective experiences instead of health benefits. J Sport Health Sci. 2024;13(4):445–50. doi: 10.1016/j.jshs.2022.10.005 36334885 PMC11184383

[12] Cheval B, Boisgontier MP. The Theory of Effort Minimization in Physical Activity. Exercise and Sport Sciences Reviews. 2021 Jul;49(3):168–78. doi: 10.1249/JES.0000000000000252 34112744 PMC8191473

[13] Loewenstein G. Because it is there: The challenge of mountaineering… for utility theory. Kyklos. 1999;52(3):315–43. doi: 10.1111/j.1467-6435.1999.tb00221.x

[14] Bieleke M, Stähler J, Wolff W, Schüler J. Development and Validation of the Value of Physical Effort (VoPE) Scale. Collabra: Psychology. 2025;11(1). doi: 10.1525/collabra.140736

[15] Stähler J, Bieleke M, Wolff W, Gruber M, Barzyk P, Schüler J. The experience of physical effort is related to the value of physical effort during low, moderate, and vigorous exercise. OSF. https://osf.io/eh2b6_v1. 2025. Accessed 2025 April 24.

[16] Wolff W, Stähler J, Schüler J, Bieleke M. On the specifics of valuing effort: a developmental and a formalized perspective on preferences for cognitive and physical effort. Peer Community Journal. 2024;4. doi: 10.24072/pcjournal.444

[17] Cheval B, Maltagliati S, Desplanques F, Wolff W. Unpacking the dynamic role of physical effort in shaping behavior. Trends Cogn Sci. 2025;29(12):1086–96. doi: 10.1016/j.tics.2025.04.012 40441966

[18] Eisenberger R. Learned industriousness. Psychological Review. 1992;99(2):248. 1594725 10.1037/0033-295x.99.2.248

[19] Bernacer J, Martinez-Valbuena I, Martinez M, Pujol N, Luis EO, Ramirez-Castillo D, et al. An amygdala-cingulate network underpins changes in effort-based decision making after a fitness program. Neuroimage. 2019;203:116181. doi: 10.1016/j.neuroimage.2019.116181 31521824

[20] Clay G, Mlynski C, Korb FM, Goschke T, Job V. Rewarding cognitive effort increases the intrinsic value of mental labor. Proc Natl Acad Sci U S A. 2022;119(5):e2111785119. doi: 10.1073/pnas.2111785119 35101919 PMC8812552

[21] Lin H, Westbrook A, Fan F, Inzlicht M. An experimental manipulation of the value of effort. Nat Hum Behav. 2024;8(5):988–1000. doi: 10.1038/s41562-024-01842-7 38438651

[22] Ekkekakis P, Petruzzello SJ. Acute Aerobic Exercise and Affect. Sports Med. 1999;28(5):337–47. doi: 10.2165/00007256-199928050-00005 10593646

[23] Saanijoki T, Tuominen L, Tuulari JJ, Nummenmaa L, Arponen E, Kalliokoski K, et al. Opioid Release after High-Intensity Interval Training in Healthy Human Subjects. Neuropsychopharmacology. 2018;43(2):246–54. doi: 10.1038/npp.2017.148 28722022 PMC5729560

[24] Heyman E, Gamelin F-X, Goekint M, Piscitelli F, Roelands B, Leclair E, et al. Intense exercise increases circulating endocannabinoid and BDNF levels in humans--possible implications for reward and depression. Psychoneuroendocrinology. 2012;37(6):844–51. doi: 10.1016/j.psyneuen.2011.09.017 22029953

[25] Steele J. What is (perception of) effort? Objective and subjective effort during attempted task performance. PsyArXiv. 2020. doi: 10.31234/osf.io/kbyhm

[26] Åstrand PO. Work tests with the bicycle ergometer. Monark. 1965.

[27] Borg G. Borg’s Perceived Exertion and Pain Scales. Champaign, IL, USA: Human Kinetics. 1998.

[28] Lopez-Gamundi P, Yao Y-W, Chong TT-J, Heekeren HR, Mas-Herrero E, Marco-Pallarés J. The neural basis of effort valuation: A meta-analysis of functional magnetic resonance imaging studies. Neurosci Biobehav Rev. 2021;131:1275–87. doi: 10.1016/j.neubiorev.2021.10.024 34710515

[29] Zénon A, Sidibé M, Olivier E. Disrupting the Supplementary Motor Area Makes Physical Effort Appear Less Effortful. J Neurosci. 2015;35(23):8737–44. doi: 10.1523/JNEUROSCI.3789-14.2015 26063908 PMC6605204

[30] Hogan PS, Galaro JK, Chib VS. Dissociable roles of ventromedial prefrontal cortex and anterior cingulate in subjective valuation of prospective effort. 2016. doi: 10.1101/079467 PMC6735256 30541111

[31] Kramer A, Kümmel J, Mulder E, Gollhofer A, Frings-Meuthen P, Gruber M. High-Intensity Jump Training Is Tolerated during 60 Days of Bed Rest and Is Very Effective in Preserving Leg Power and Lean Body Mass: An Overview of the Cologne RSL Study. PLoS One. 2017;12(1):e0169793. doi: 10.1371/journal.pone.0169793 28081223 PMC5231329

[32] Gruber M, Howaldt J, Schwenk M, Moreno-Villanueva M, Bieleke M, Schüler J, et al. Effects of jump training on power, strength, balance and aerobic performance in nonexercising young adults. SportRxiv. 2025. 10.3389/fspor.2026.1746624 PMC12979136 41835023

[33] Beaver WL, Wasserman K, Whipp BJ. A new method for detecting anaerobic threshold by gas exchange. J Appl Physiol (1985). 1986;60(6):2020–7. doi: 10.1152/jappl.1986.60.6.2020 3087938

[34] Binder RK, Wonisch M, Corra U, Cohen-Solal A, Vanhees L, Saner H, et al. Methodological approach to the first and second lactate threshold in incremental cardiopulmonary exercise testing. Eur J Cardiovasc Prev Rehabil. 2008;15(6):726–34. doi: 10.1097/HJR.0b013e328304fed4 19050438

[35] Dahmen T. Modeling, simulation, and optimization of pacing strategies for road cycling on realistic tracks. Konstanz: Universität Konstanz. 2016. https://kops.uni-konstanz.de/handle/123456789/40448

[36] Borg E, Borg G, Larsson K, Letzter M, Sundblad B‐M. The Borg CR Scales Folder. Scandinavian Journal of Medicine & Science in Sports. 2010;20(4):644–50. doi: 10.1111/j.1600-0838.2009.00985.x 19602182

[37] Pageaux B. Perception of effort in Exercise Science: Definition, measurement and perspectives. Eur J Sport Sci. 2016;16(8):885–94. doi: 10.1080/17461391.2016.1188992 27240002

[38] Oostenveld R, Praamstra P. The five percent electrode system for high-resolution EEG and ERP measurements. Clin Neurophysiol. 2001;112(4):713–9. doi: 10.1016/s1388-2457(00)00527-7 11275545

[39] Aasted CM, Yücel MA, Cooper RJ, Dubb J, Tsuzuki D, Becerra L, et al. Anatomical guidance for functional near-infrared spectroscopy: AtlasViewer tutorial. Neurophotonics. 2015;2(2):020801. doi: 10.1117/1.NPh.2.2.020801 26157991 PMC4478785

[40] Oulu, Finland: Kubios Oy. 2023.

[41] Laborde S, Mosley E, Thayer JF. Heart Rate Variability and Cardiac Vagal Tone in Psychophysiological Research - Recommendations for Experiment Planning, Data Analysis, and Data Reporting. Front Psychol. 2017;8:213. doi: 10.3389/fpsyg.2017.00213 28265249 PMC5316555

[42] Task Force of the European Society of Cardiology, North American Society of Pacing Electrophysiology. Heart rate variability. Eur Heart J. 1996;17. 8737210

[43] Huppert TJ, Diamond SG, Franceschini MA, Boas DA. HomER: a review of time-series analysis methods for near-infrared spectroscopy of the brain. Appl Opt. 2009;48(10):D280-98. doi: 10.1364/ao.48.00d280 19340120 PMC2761652

[44] Gao L, Wei Y, Wang Y, Wang G, Zhang Q, Zhang J, et al. Hybrid motion artifact detection and correction approach for functional near-infrared spectroscopy measurements. J Biomed Opt. 2022;27(02). doi: 10.1117/1.JBO.27.2.025003 PMC8871689 35212200

[45] Delpy DT, Cope M, van der Zee P, Arridge S, Wray S, Wyatt J. Estimation of optical pathlength through tissue from direct time of flight measurement. Phys Med Biol. 1988;33(12):1433–42. doi: 10.1088/0031-9155/33/12/008 3237772

[46] Bunc V, Heller J, Leso J. Kinetics of heart rate responses to exercise. J Sports Sci. 1988;6(1):39–48. doi: 10.1080/02640418808729792 3404576

[47] Wolff W, Bieleke M, Hirsch A, Wienbruch C, Gollwitzer PM, Schüler J. Increase in prefrontal cortex oxygenation during static muscular endurance performance is modulated by self-regulation strategies. Sci Rep. 2018;8(1):15756. doi: 10.1038/s41598-018-34009-2 30361513 PMC6202346

[48] R Core Team. R: A Language and Environment for Statistical Computing. Vienna, Austria: R Foundation for Statistical Computing. 2023.

[49] JASP Team. JASP. 2024.

[50] Kruschke JK. Bayesian Analysis Reporting Guidelines. Nat Hum Behav. 2021;5(10):1282–91. doi: 10.1038/s41562-021-01177-7 34400814 PMC8526359

[51] Cohen J. Statistical power analysis for the behavioral sciences. 2nd ed. Hoboken: Taylor and Francis. 2013.

[52] Jeffreys H. Theory of probability. Oxford, England: Oxford University Press. 1961.

[53] Giboin L-S, Gruber M, Kramer A. Task-specificity of balance training. Hum Mov Sci. 2015;44:22–31. doi: 10.1016/j.humov.2015.08.012 26298214

[54] Laurence NC, Labuschagne LG, Lura BG, Hillman KL. Regular Exercise Enhances Task-Based Industriousness in Laboratory Rats. PLoS One. 2015;10(6):e0129831. doi: 10.1371/journal.pone.0129831 26083255 PMC4470589

[55] Teixeira DS, Ekkekakis P, Andrade A, Rodrigues F, Evmenenko A, Faria J, et al. Preference for and tolerance of the intensity of exercise questionnaire (PRETIE-Q): validity, reliability and gender invariance in Portuguese health club exercisers. Curr Psychol. 2023;42(5):4119–32. doi: 10.1007/s12144-021-01718-3

[56] Sullivan-Toole H, DePasque S, Holt-Gosselin B, Galván A. Worth working for: The influence of effort costs on teens’ choices during a novel decision making game. Dev Cogn Neurosci. 2019;37:100652. doi: 10.1016/j.dcn.2019.100652 31075712 PMC6969283

[57] Perrey S. Non-invasive NIR spectroscopy of human brain function during exercise. Methods. 2008;45(4):289–99. doi: 10.1016/j.ymeth.2008.04.005 18539160

[58] Hyland-Monks R, Marchant D, Cronin L. Self-Paced Endurance Performance and Cerebral Hemodynamics of the Prefrontal Cortex: A Scoping Review of Methodology and Findings. Percept Mot Skills. 2022;129(4):1089–114. doi: 10.1177/00315125221101017 35609231 PMC9301167

[59] Ferrari M, Quaresima V. A brief review on the history of human functional near-infrared spectroscopy (fNIRS) development and fields of application. Neuroimage. 2012;63(2):921–35. doi: 10.1016/j.neuroimage.2012.03.049 22510258

[60] Gottfried JA, O’Doherty J, Dolan RJ. Encoding predictive reward value in human amygdala and orbitofrontal cortex. Science. 2003;301(5636):1104–7. doi: 10.1126/science.1087919 12934011

[61] Corbit LH, Balleine BW. The General and Outcome-Specific Forms of Pavlovian-Instrumental Transfer Are Differentially Mediated by the Nucleus Accumbens Core and Shell. J Neurosci. 2011;31(33):11786–94. doi: 10.1523/jneurosci.2711-11.2011 21849539 PMC3208020

[62] Schultz W, Tremblay L, Hollerman JR. Reward processing in primate orbitofrontal cortex and basal ganglia. Cerebral Cortex. 2000;10(3):272–83. doi: 10.1093/cercor/10.3.272 10731222

