# Supplementary material for: An 8-week jump training did not boost effort value or the willingness to exert effort in a student sample
Source: PLoS One. 2026 Jul 16;21(7):e0352897. doi: 10.1371/journal.pone.0352897 (PMC13375129; doi:10.1371/journal.pone.0352897)
Supplement: Attachment — Submitted filename: Response to Reviewers.docx [file pone.0352897.s002.docx]

**Point-by-point reply**

**Manuscript number: #PONE-D-25-42417**

**Article title: An 8-week jump training did not boost effort value or the willingness to exert effort in a student sample**

--------------------------------------------- comments Christopher Kirk ----------------------------------------

**Dear XXX,**

**Thank you for submitting your manuscript to PLOS ONE. After careful consideration, we feel that it has merit but does not fully meet PLOS ONE’s publication criteria as it currently stands. Therefore, we invite you to submit a revised version of the manuscript that addresses the points raised during the review process.**

**Please submit your revised manuscript by Dec 25 2025 11:59PM. If you will need more time than this to complete your revisions, please reply to this message or contact the journal office at plosone@plos.org. When you're ready to submit your revision, log on to https://www.editorialmanager.com/pone/ and select the 'Submissions Needing Revision' folder to locate your manuscript file.**

**Please include the following items when submitting your revised manuscript:**

- **A rebuttal letter that responds to each point raised by the academic editor and reviewer(s). You should upload this letter as a separate file labeled 'Response to Reviewers'.**
- **A marked-up copy of your manuscript that highlights changes made to the original version. You should upload this as a separate file labeled 'Revised Manuscript with Track Changes'.**
- **An unmarked version of your revised paper without tracked changes. You should upload this as a separate file labeled 'Manuscript'.**

**If you would like to make changes to your financial disclosure, please include your updated statement in your cover letter. Guidelines for resubmitting your figure files are available below the reviewer comments at the end of this letter.**

**If applicable, we recommend that you deposit your laboratory protocols in protocols.io to enhance the reproducibility of your results. Protocols.io assigns your protocol its own identifier (DOI) so that it can be cited independently in the future. For instructions see: https://journals.plos.org/plosone/s/submission-guidelines#loc-laboratory-protocols. Additionally, PLOS ONE offers an option for publishing peer-reviewed Lab Protocol articles, which describe protocols hosted on protocols.io. Read more information on sharing protocols at https://plos.org/protocols?utm_medium=editorial-email&utm_source=authorletters&utm_campaign=protocols.**

**We look forward to receiving your revised manuscript.**

**Kind regards,**

**Christopher Kirk, PhD**

**Academic Editor**

**PLOS ONE**

**Journal Requirements:**

**When submitting your revision, we need you to address these additional requirements.**

**1.Please ensure that your manuscript meets PLOS ONE's style requirements, including those for file naming. The PLOS ONE style templates can be found at https://journals.plos.org/plosone/s/file?id=wjVg/PLOSOne_formatting_sample_main_body.pdf and**

**https://journals.plos.org/plosone/s/file?id=ba62/PLOSOne_formatting_sample_title_authors_affiliations.pdf**

Thank you for pointing us to these templates. We have revised the manuscript accordingly.

**2. Thank you for stating the following financial disclosure:**

**“The authors gratefully acknowledge funding from the Committee on Research (AFF) at the University of Konstanz for the research initiative "ProPELL: Promoting Physical Exercise in Lab and Life“”**

**Please state what role the funders took in the study. If the funders had no role, please state: "The funders had no role in study design, data collection and analysis, decision to publish, or preparation of the manuscript."**

**If this statement is not correct you must amend it as needed.**

**Please include this amended Role of Funder statement in your cover letter; we will change the online submission form on your behalf.**

We appreciate your comment and have now included the Role of Funder statement in the cover letter.

**3. We note that the grant information you provided in the ‘Funding Information’ and ‘Financial Disclosure’ sections do not match.**

**When you resubmit, please ensure that you provide the correct grant numbers for the awards you received for your study in the ‘Funding Information’ section.**

Thank you for pointing this out. We have now revised this information.

**4. Please note that funding information should not appear in any section or other areas of your manuscript. We will only publish funding information present in the Funding Statement section of the online submission form. Please remove any funding-related text from the manuscript.**

We appreciate this guidance and have accordingly removed the funding statement from the manuscript.

**5. Please include your full ethics statement in the ‘Methods’ section of your manuscript file. In your statement, please include the full name of the IRB or ethics committee who approved or waived your study, as well as whether or not you obtained informed written or verbal consent. If consent was waived for your study, please include this information in your statement as well.**

Thank you for this comment. We have accordingly included the requested information in the Methods section (p. 13).

**6. If the reviewer comments include a recommendation to cite specific previously published works, please review and evaluate these publications to determine whether they are relevant and should be cited. There is no requirement to cite these works unless the editor has indicated otherwise.**

**Additional Editor Comments:**

**Please pay particular attention to comments regarding how the fNIRS data have been analysed and presented.**

**There are also several comments requesting that some statements in the discussion need to be modified to ensure they are fully in keeping with the what the data show.**

**The length of the introduction needs to be reduced considerably, and please ensure that the required information is all presented in the appropriate sections. The introduction also needs to provide a more robust theoretical justification for the study design and aims.**

**You may disregard comments from Reviewer 2 in this instance, as they have only provided 2 sentences that do not provide any value to the assessment of this manuscript. This reviewer will not be invited to review the next submission of this work.**

We would like to express our sincere gratitude to you and the reviewers for your constructive feedback on our manuscript. We have carefully considered your thoughtful comments and made revisions accordingly. In particular, we focused on reviewing the fNIRS-related sections, clarifying the discussion of the results, streamlining the introduction and shortening the manuscript to about 20 pages.

We believe all changes have greatly improved the quality of our manuscript, and we truly appreciate your time and effort in helping us enhance our work.

In the following, you will find our responses highlighted in blue, along with citations of the manuscript in bold and italics. Newly added passages related to the comment are indicated in red to ensure clarity.

-------------------------------------------------- comments Reviewer #1 ----------------------------------------

**Thank you for the invitation to review. I have the following comments:**

1. **Introduction:**
a. **In several places the text uses “effort cost” and “effort value” interchangeably, although these are two distinct psychological and neurobiological constructs. It would be helpful to emphasise more clearly that effort can be both costly and valuable at the same time, and that the perception of this relationship depends on context, the individual, and prior experience.**

We appreciate this helpful comment and fully agree that effort cost and value represent distinct constructs. We have carefully revised the manuscript to ensure that cost and value are no longer used interchangeably. In addition, we explicitly clarified that these constructs are conceptually distinct and may co-occur. We believe these revisions improve the conceptual clarity of the manuscript

**“*Conversely, accumulating evidence indicates that physical effort can simultaneously be perceived as valuable – at least to some degree, for some people, and in some situations (12,20).” (pp. 4-5)***

***“Consistent with this, theoretical work suggests mechanisms by which reduced effort aversiveness can increase individuals’ tendency to mobilize effort over time (see 12). One prominent example is Learned Industriousness, which proposes that repeatedly pairing effort with reward reduces effort’s aversiveness and increases willingness to exert effort (30). Notably, effort aversiveness can in principle be reduced via two mechanisms: by decreasing perceived effort costs and/or by increasing VoPE. However, whether Learned Industriousness also increases VoPE remains unclear, as both theoretical and empirical work have predominantly focused on reductions in effort costs (31–34) rather than potential increases in VoPE (20).” (p. 5)***

**“*Thus, while previous research has begun to explore near-transfer effects in decision-making (31), it remains largely unclear whether regular physical effort alters how effort is valued (rather than its costs) during actual exertion, whether such changes generalize across tasks, and how they depend on exercise intensity (20).*” (p. 7)**

b. **The impact of training on VoPE (Value of Physical Effort) may be modulated by factors such as previous sporting experience, temperamental traits, intrinsic motivation, or genetic predispositions (e.g. differences in the dopaminergic reward system). The text focuses mainly on group-level effects while overlooking potentially important individual differences.**

We agree that the impact of training on the value of physical effort may vary across individuals as a function of prior sporting experience, motivational traits and other individual differences. As our study focuses on group-level effects, we now explicitly acknowledge this in the discussion and note that future work is needed to examine such moderating influences. (see our answer to your comment 9e)

**“Another possible explanation lies in the developmental sensitivity of learning VoPE (20). Adolescents appear to be less sensitive to effort costs and exert more effort relative to task demands and rewards at stake than adults (78,79). This period of heightened responsiveness may facilitate the learning of the relationship between effort and reward, making it easier to internalize the contingency that greater effort leads to greater rewards. By contrast, by the time individuals reach early adulthood, as in the current sample, it may be more challenging to relearn or modify their VoPE. While this observation opens an interesting avenue for developmental research, it is also important to consider potential moderating factors such as previous learning experiences in the sport context, or genetic predispositions (e.g., differences in the dopaminergic reward system) that may influence how VoPE is learned. Future research should therefore test similar interventions with adolescent samples and systematically examine individual differences that may shape sensitivity to learning VoPE*.*” (pp. 37-38)**

c. **The authors correctly note that most studies concern low to moderate intensities. However, there is no clear indication that high-intensity efforts (e.g. maximal intervals) may involve different neurophysiological mechanisms (e.g. stronger engagement of the opioid and endocannabinoid systems), which could alter the relationship between effort cost and value.**

This is an important comment, and we thank the reviewer for raising this point. In response, we have revised the Introduction to explicitly note that different exercise intensities may engage distinct neurophysiological mechanisms, particularly with respect to the endogenous opioid and endocannabinoid systems. These intensity-related differences may, in turn, influence the valuation of physical effort, thereby strengthening the rationale for considering high-intensity exercise (see also Reviewer #4, comment 1).

**“*In Bernacer et al. (31), initially inactive participants completed a three-month low-intensity exercise program, after which effort costs had less influence on effort-based decisions, accompanied by decreased effort-related activity in the anterior cingulate cortex (ACC) (31). These findings suggest that regular exercise may reduce effort costs and alter neural responses to effort (31). However, as effort intensity was low and higher intensities are often valued less (26) and perceived as more aversive (39–41), it remains unclear whether training at higher intensities, might yield stronger effects on effort valuation. Notably, high-intensity exercise may engages additional neurophysiological mechanisms, such as increased activation of the endogenous opioid and endocannabinoid systems, which have been linked to affective responses and reward processing during intense exertion (39,40). Thus, while previous research has begun to explore near-transfer effects in decision-making (31), it remains largely unclear whether regular physical effort alters how effort is valued (rather than its costs) during actual exertion, whether such changes generalize across tasks, and how they depend on exercise intensity (20).*” (pp. 6-7)**

d. **The discussion of near- and far-transfer is valuable, but it lacks an explicit acknowledgement that in the literature on physical effort the transfer of effects is far harder to demonstrate than in cognitive effort research. Learning mechanisms in the context of physical activity may be more task-specific, which should be highlighted as a potential limitation.**

Thank you for this important comment. We have revised the Discussion to explicitly acknowledge that evidence for transfer effects in the domain of physical effort is considerably more limited than in cognitive effort research. In particular, we now highlight that far-transfer effects are more difficult to demonstrate for physical effort and that learning mechanisms in the context of physical activity tend to be more task- and context-specific.

**“*Importantly, evidence for transfer effects in the domain of physical effort is considerably more limited and less consistent than in cognitive effort research, with learning effects often appearing to be task- and context-specific (75). Although previous studies have demonstrated both near- and far-transfer effects, like reduced perceived physical effort costs and increased industriousness, in both animals (76) and humans (31), the conditions under which such transfer effects occur remain poorly understood.*“ (p. 34)**

e. **The argument relies on a few fMRI studies concerning the ACC and vmPFC. However, the methodological limitations are not addressed, such as the low statistical power of neuroimaging studies or the problem of reverse inference. Assigning overly direct functions to brain regions (e.g. ACC = “cost”, vmPFC = “net value”) risks oversimplification.**

Thank you for highlighting this important conceptual and methodological concern. We have revised the Discussion and the interpretation of our results now explicitly takes these limitations into account. In line with your suggestions, we now clarify that the assignment of roles to specific brain regions represents a simplified framework, rather than definitive functional localization. We further emphasize that this simplification is useful at the current stage of research but should be interpreted with caution when discussing our findings.

Discussion: “***Finally, the current study assessed neural activation during periods of (partially) intense physical exertion, which offers valuable ecological validity. However, physical exercise involves multiple concurrent cognitive processes (80,81), which may obscure neural mechanisms specific to VoPE. Moreover, interpreting activation in specific brain regions necessarily relies on simplified heuristic functional attributions, which entails the risk of oversimplification and reverse inference. While this approach was useful for addressing the present research question at the current stage of the literature, it may have constrained our ability to draw clearer inferences, as network neuroscience emphasizes that functions likely emerge from interactions rather than one-to-one mappings between regions and processes. Future research could expand on this by using whole brain functional analyses, examining neural activation during physical effort-related decision-making (31) or by employing more localized physical effort paradigms, such as grip force tasks, rather than whole-body exercises like cycling.***” **(p. 38)**

f. **The text provides a good summary of the state of knowledge, but practical implications are missing. If regular training can alter the neural valuation of effort, it would be important to discuss how this knowledge could be applied in designing health interventions, e.g. in programmes for activating older adults, in rehabilitation, or in strategies to counteract sedentary lifestyles.**

We appreciate this valuable comment and fully agree that highlighting potential practical implications strengthens the relevance of the work. In response, we have briefly expanded the Introduction to address this point, while keeping the section concise to maintain the overall flow of the manuscript.

**“*Conversely, accumulating evidence indicates that physical effort can simultaneously be perceived as valuable – at least to some degree, for some people, and in some situations (12,20). Contrary to the effort minimization theory (23), some people seem to seek out effortful activities for the mere sake of exerting effort (24), such as choosing a physically challenging hiking route just because they like the effort. This apparent preference might reflect a higher subjective valuation of physical effort. Recent studies indicate that higher Value of Physical Effort (VoPE) (25) is associated with a more positive effort experience during exertion (26), reduced boredom in physically effortful tasks (27), and greater overall physical activity (25,28). Enhancing VoPE could therefore be a promising way to help people be more physically active (29), with potential applications in rehabilitation, programs for older adults, or interventions targeting sedentary lifestyles. If VoPE can be modified, it may offer a complementary pathway to traditional approaches.*” (pp. 4-5)**

2. **I recommend shortening the Methods section – it is overly lengthy and repetitive.**

We appreciate this helpful recommendation and have revised the Methods section accordingly. Particular emphasis was placed on the Procedure and Data Analysis sections, as requested by Reviewer #3 in comments 3 and 4. In doing so, we removed redundancies and streamlined the text to improve clarity and conciseness and reduced the text by 25% (≈ 1.5 pages).

3. **Procedure and Materials:**
a. **Selection of time window for fNIRS – using only the second minute of each interval (excluding onset and offset) is methodologically justified in terms of signal stabilisation and limiting artefacts, but may restrict the ability to capture effort dynamics. A comparative analysis with the full interval duration should be considered to ensure that important changes were not missed.**

We appreciate this thoughtful suggestion. We carefully considered the selection of the fNIRS time window. As described in the manuscript, we excluded the first minute of the ride because this phase is characterized by a different effort experience than once the target intensity has been maintained for a full minute. This could lead to a ramping-up effect. The verbal query took place at the end of the interval; unfortunately, this timing is not ideal, as the neurological data from the third minute would also have been of interest. However, due to the verbal responses, these data are contaminated by motion artifacts and are not usable for analysis.

For this reason, we specified the analysis to include only the second minute, which contains minimal motion artifacts and reflects a stable effort level without a pronounced ramping-up phase, as explained in the manuscript:

“***Only the second minute of each interval was used, excluding the initial 60-second ramp-up phase, during which heart rate increases and only stabilizes over time (69), as well as the query period at the end of each interval, where participants gave verbal responses.***” **(p. 18)**

We also conducted our analysis including minutes 1-2 and found no differences in the results. For reasons of brevity, this detail was omitted from the manuscript. However, we should be happy to include it in the manuscript or OSF should the editor or reviewers consider it relevant.

b. **Definition of exercise intensity – basing intensity on metabolic thresholds (AT, RCP) is a strength as it allows individualisation of loads. Nevertheless, adopting fixed values (e.g. 85% AT, mean of AT and RCP, RCP + 5%) may produce differences in the subjective perception of effort between participants. Additional indices (e.g. % VO₂max, % HRmax) would be valuable.**

Thank you very much for this thoughtful comment. This is an important point that we considered carefully during the development of the study design. We deliberately chose not to prescribe the intervals based on fixed percentages of maximal heart rate or VO_2_max, as intensity markers relative to the maximum are not perceived as subjectively equivalent across individuals.

Specifically, the required energy can be supplied through different metabolic pathways across individuals. For some participants, 50% of their VO_2_max still reflects predominantly aerobic energy provision, whereas for others this level already entails a substantial anaerobic contribution. Because perceived exertion is strongly influenced by the underlying metabolic processes, it was particularly important for us to distinguish between these metabolic states when defining exercise intensity.

The metabolic thresholds, in contrast, reflect points at which a transition in metabolic regulation occurs. For this reason, we consider metabolic thresholds to provide a more meaningful and physiologically relevant basis for describing and standardizing exercise intensity than fixed percentages of HR max or VO_2_max.

Given the current length of the manuscript, we refrained from elaborating these considerations in the manuscript. However, we are happy to do so upon request.

c. **The use of single-item questions and verbal scales is convenient but may reduce measurement sensitivity. Participants might struggle to differentiate states precisely in short time frames. Was test–retest reliability (ICC) checked? – 10.1016/j.jcm.2016.02.012**

Thank you for this helpful comment. We followed up on your suggestion and examined the test-retest reliability of perceived exertion (RPE) and state VoPE across the three intensity intervals. RPE showed moderate reliability across intensities (ICC_low_ = 0.61, ICC_moderate_ = 0.51, ICC_vigorous_ = 0.54), whereas state VoPE showed moderate reliability at low intensity and good reliability at moderate and vigorous intensities (ICClow = 0.74, ICCmoderate = 0.75, ICCvigorous = 0.78).

“***Perceived exertion (RPE) was assessed with the question “How strong do you exert yourself right now?” using the Category Ratio 10 scale (47,62), ranging from 0 (“no effort at all”) to 10 (“maximal”) or 11 (“even more than max”) (63). Test-retest reliability was ICC = 0.61 for low, ICC = 0.51 for moderate, and ICC = 0.54 for vigorous intensity.***

***State value of physical effort (state VoPE) was assessed with “How much do you like exerting yourself right now?” using a bipolar 10-point scale, ranging from -5 (“I don’t like it at all”), 0 (“neutral”), to +5 (“I like it very much”). Test-retest reliability for state VoPE was ICC = 0.74 for low, ICC = 0.75 for moderate, and ICC = 0.78 for vigorous intensity.***” **(p. 15)**

Since participants were free to choose the intensity for the free ride task, the actual intensities likely differed between M1 and M2. For this reason, we did not calculate ICCs for the single-item measures of the free ride task.

d. **fNIRS measurement – the chosen optode configuration and sensitivity simulations support measurement reliability, but fNIRS in the vmPFC and preSMA is particularly susceptible to motion artefacts and circulatory confounds. The procedures for signal quality control (e.g. filtering, artefact correction, trial rejection) should be described in more detail.**

Thank you for raining this point. As described in the data preprocessing section, motion artifacts as well as cardiac- and respiratory-related physiological noise were carefully addressed during fNIRS preprocessing. Importantly, preprocessing parameters were specifically adapted to the used task, which was expected to induce an increased number of movement-related artifacts. This task-specific adjustment allowed us to reduce motion-related noise while minimizing the risk of over-filtering and excessive data loss.

To provide a clearer overview of the preprocessing pipeline, we have summarized the individual fNIRS preprocessing steps, including the applied HOMER3 functions and their purpose, in a tabular format for the reviewers.

| Processing step | HOMER3 function | Purpose for data preparation |
| --- | --- | --- |
| Channel quality pruning | hmrR_pruneChannels  dRange: 1e-02 – 3e+00  SNRthresh:2  SDrange: 0-45 | Excludes channels with unreliable signal quality (too weak/strong intensity or low signal-to-noise ratio) to ensure valid data input for further processing |
| Correction of negative intensity values | hmrR_PreprocessIntensity_Negative | Corrects physically implausible negative intensity values that can arise from hardware or recording artifacts. |
| Handling of missing data | hmrR_PreprocessingIntensity_NAN (spline interpolation) | Replaces short segments of missing data to maintain continuity of the time series and avoid disruptions in downstream analyses. |
| Conversion to optical density | hmrR_Intensity2OD | Transforms raw light intensity into optical density, a prerequisite for motion correction and hemoglobin concentration estimation. |
| Motion artifact detection | hmrR_MotionArtifactByChannel  tMotion = 0.5  tMask = 1.5  STDEVthresh = 50, AMPthresh = 0.2 | Identifies time segments affected by abrupt signal changes caused by participant movement. |
| Motion correction (spline interpolation) | hmrR_MotionCorrectSpline  p = 0.99 | Corrects large motion-related signal deviations by interpolating over detected artifact segments. |
| Motion correction (wavelet filtering) | hmrR_MotionCorrectWavelet  Iqr = 1.0 | Attenuates residual motion-related spikes in the wavelet domain by removing statistically extreme coefficients while preserving neural signals. |
| Temporal bandpass filtering | hmrR_BandpassFilt  0.01-0.5 Hz | Removes low-frequency signal drifts and high-frequency physiological noise (e.g., cardiac and respiratory artifacts). |
| Conversion to hemoglobin concentrations | hmrR_OD2Conc  DPF = 6.0 | Converts optical density data into changes in oxy- and deoxyhemoglobin concentration using the modified Beer-Lambert law |
| Estimation of task-related responses | hmrR_BlockAvg | Commputes the hemodynamic response function (HRF) time-locked to task intervals for subsequent statistical analysis. |

In addition, we have expanded the description of the fNIRS setup by specifying that optode performance was verified before each measurement day. This addition strengthens the methodological rigor of the manuscript by explicitly documenting quality control procedures for signal stability and data reliability.

„ ***Oxygenation in the vmPFC and preSMA was measured using a multichannel continuous-wave fNIRS system (NIRSport, NIRx Medical Technologies LLC, NY, USA) and NIRStar software (Version 15.3, NIRx Medical Technologies LLC, NY, USA 2020) at 7.81 Hz (760/850 nm) with eight emitters and eight detectors forming 16 channels (25-35 mm). Optodes were arranged according to the international 5/10 system (61) (see Fig 1a) using a custom elastic NIRScap (NIRScap, EASYCAP GmbH, Herrsching, Germany) in multiple sizes and secured with overcap (EASYCAP GmbH, Herrsching, Germany) to reduce motion artifacts and ambient light. Sensitivity was verified via Atlas Viewer simulations (62) (see Fig 1b) and optode performance was tested prior to each testing day by running a series of diagnostic procedures (e.g., gain modulation).***“ **(pp. 16-17)**

e. **The whole session lasts around two hours and involves both high-intensity exercise and additional measurement tasks. This may induce fatigue in participants, potentially affecting RPE, VoPE and physiological parameters. It should be stated how fatigue effects were controlled (e.g. recovery breaks, monitoring of resting HR between tasks).**

We appreciate this suggestion and have now clarified the procedure by explicitly highlighting the recovery breaks between the intervals as well as the break between the interval task and the free ride task.

Because the interval task was designed such that the prescribed intensity intervals were theoretically comparable in terms of exertion across participants, and because a 10-minute rest period was included between the two tasks, we did not implement additional control measures for fatigue beyond this standardized recovery phase.

“***The interval task (15 minutes) consisted of low, moderate, and vigorous intensities (randomized across participants) were completed in three-minute bouts separated by two-minute recovery breaks. Workload was individualized using the anaerobic threshold (AT) and the respiratory compensation point (RCP) derived from a Cardiopulmonary Exercise Test (CPET) (performed 2-4 days before both measurement sessions) (56). Intensities were set to 85% of AT (low); the mean of AT and RCP, calculated as (AT[W] + RCP [W]) / 2 (moderate), and 5% above RCP (vigorous) (56,57). To ensure the intended intensity, participants maintained a cadence of 85-95 revolutions per minute (rpm), while resistance levels were determined by the Powerbike software (Powerbike; 58). Participants received real-time visual feedback on cadence and target power. At the end of each interval, RPE and state VoPE were assessed. After a ten-minute recovery break, participants completed a free ride task (10 minutes), cycling a virtual route (Allensbach to Langenrain; southern Germany). They freely selected their intensity without a predefined goal. Every two minutes, RPE and state VoPE were assessed. Heart rate and hemodynamic oxygenation in the vmPFC (value-related) and preSMA (effort-intensity-related) were continuously recorded.***” **(pp. 13-14)**

4. **L241 – “training group (n = 34) or a control group (n = 28).” – According to the conducted analyses, this sample size was sufficient to detect a large effect with 80% power. Please indicate this in the manuscript – 10.1016/j.apmr.2025.05.013**

We appreciate this comment. Our study employed a 2x2 mixed design (group x time). We conducted a sensitivity analysis in G*Power and added the corresponding information for each effect to the manuscript:

***“A sensitivity analysis using G*Power indicated that, for our 2x2 mixed design and given the actual sample size and 80% power, detectable effect sizes were f = 0.31 for between-subjects effects, and f = 0.18 for within-subject effects and interactions.”* (p. 11)**

5. **L361 – “used Bayesian repeated measures ANOVAs” – Please calculate effect sizes and provide confidence intervals. For group differences (Cohen’s d or Hedges’ g), small, medium, and large effects should correspond to 0.1, 0.4, and 0.8, respectively – 10.1016/j.apmr.2025.05.013**

Thank you very much for this important comment. In response, we have added to the Data Analysis section that effect sizes and corresponding confidence intervals were calculated for all identified effects. We believe that this addition improves the transparency and interpretability of the reported results.

**“*Effect sizes were calculated to quantify the strength of any observed effects using Cohen’s d along with corresponding 95% confidence intervals (73).*” (p. 20)**

6. **L366 – Bayes Factors and Cohen’s d represent different approaches: BF indicates the strength of evidence for a hypothesis, while d describes the magnitude of the effect. Literature (e.g. Lakens, 2013; van Doorn et al., 2021) often recommends reporting both, as they complement one another: Bayes Factor = evidential strength, Cohen’s d = effect size irrespective of evidence sufficiency.**

See answer to comment 5 and 7.

7. **Results – Please highlight which findings fall within medium and which within large effect sizes. In the conclusions, please base interpretations only on significant p values and large effect sizes.**

We appreciate this insightful comment. We have now reported effect sizes for all statistically identified effects in the Results section and provided an interpretation of their magnitude.

**“*The Interval effect was further supported by the second-highest Bayes Factor among candidate models, BF10 = 37.97, P(M/data) = .31, providing very strong evidence. Post hoc comparisons indicated strong evidence that state VoPE ratings were lower during the vigorous interval (M = 1.26, SD = 2.13) compared to the low (M = 1.79, SD = 1.89), BF10 = 167.43, Oddspost = 98.35, d = 0.26, 95% CI [0.01, 0.51], and moderate intervals, BF10 = 140.77, Oddspost = 82.69, d = 0.23, 95% CI [-0.02, 0.48], with both differences corresponding to small effect sizes. Substantial evidence supported equivalent ratings between the low and moderate (M = 1.73, SD = 1.98) intervals, BF10 = 0.12, Oddspost = 0.07 (see Table 1 and Fig 2a; for complete results table, see OSF).*” (pp. 25-26)**

**“*The Interval effect was further supported by the highest Bayes Factor among all candidate models, BF10 = 401155.86, P(M/data) = .60, providing decisive evidence. Post hoc comparisons indicated strong evidence that the HbR concentration in the vmPFC was lower during the low interval compared to the moderate (BF10 = 223.31, Oddspost = 131.17, d = 0.41, 95% CI [0.16, 0.67], moderate effect) and vigorous intervals (BF10 = 7.23*10+7, Oddspost = 4.25*10+7, d = 0.64, 95% CI [0.37, 0.90], moderate-to-large effect). Evidence for a vmPFC HbR difference between the moderate and vigorous intervals was inconclusive, BF10 = 0.91, Oddspost = 0.54 (see Table 1 and Fig 2b).*” (pp. 27-28)**

**“*The Interval effect was further supported by the highest Bayes Factor among all candidate models, BF10 = 120.25, P(M/data) = .37, providing decisive evidence. Post hoc comparisons indicated strong evidence that the preSMA HbO concentration was lower during the vigorous compared to the low (BF10 = 54483.89, Oddspost = 32003.89, d = 0.45, 95% CI [0.19, 0.71], moderate effect) and moderate intervals (BF10 = 10.50, Oddspost = 6.17, d = 0.31, 95% CI [0.05, 0.57], small-to-moderate effect). There was substantial evidence in favor of similar preSMA HbO concentrations between the low and moderate intervals (BF10 = 0.23, Oddspost = 0.14) (see Table 1 and S2 in the OSF).*” (pp. 28-29)**

**“*The Interval effect was also supported by the second-highest Bayes Factor among all candidate models, BF10 = 19943.26, P(M/data) = .31, providing decisive evidence. Post hoc comparisons indicated strong evidence for higher preSMA HbR concentrations during the vigorous compared to the low (BF10 = 134766.05, Oddspost = 79161.72, d = 0.72, 95% CI [0.45, 0.98], moderate-to-large effect) and moderate intervals (BF10 = 90.32, Oddspost = 53.05, d = 0.43, 95% CI [0.17, 0.69], moderate effect). Evidence was inconclusive about the difference between the moderate and the low interval, BF10 = 2.01, Oddspost = 1.29 (see Table 1).*” (pp. 29-30)**

**“*Bayesian repeated measures ANOVA tested whether state VoPE would increase in the TG while remaining stable in the CG. The best model including the Time x Condition interaction, also included main effects of Time and Condition, BF01 = 6.15, P(M/data) = .03, providing substantial evidence for the null hypothesis (76) (for complete results tables, see OSF). Model-averaged results indicated evidence against including the main effect of Time, BFexcl = 6.27, P(excl./data) = .81, and the Time x Condition interaction, BFexcl = 7.50, P(excl./data) = .97 (see Fig 3d). The main effect for Condition, BFexcl = 0.48, P(excl/data) = .24, was anecdotally supported. Post hoc comparisons for Condition further indicated substantial evidence that state VoPE was higher in the TG (M = 2.16, SD = 1.64) than the CG (M = 1.17, SD = 2.17) (BF01 = 0.05, Oddspost = 0.05, d = 0.55, 95% CI [0.19, 0.92], moderate effect).*” (p. 32)**

8. **L383 – I suggest closing the paragraph with the statistical outcomes, while the procedural details described by the authors should, in my opinion, be moved earlier.**

Thank you for this helpful comment. In response, we have revised the structure of the Data Analysis section to improve clarity and readability. This information is now organized in a more chronological manner, with data preprocessing described first, followed by the description of the statistical analyses. We believe that this reorganization facilitates comprehension of the analysis pipeline and provides a clearer overview of the analytical steps.

9. **Discussion and Conclusions:**
a. **The authors suggest that the lack of change in VoPE indicates its relative stability. However, it is possible that the effect was masked by the specifics of the intervention (high intensity, lack of systematic rewards). Therefore, the conclusion about “stability” should be stated more cautiously, with limitations of protocol sensitivity underlined.**

Thank you very much for this important and helpful comment. Although we had already aimed to phrase this statement cautiously, we have now revised the relevant sections in both the Discussion and the Conclusion to address your concern more explicitly:

**„** ***This study investigated whether regular exposure to physical effort through high-intensity exercise alters individuals’ value of physical effort (VoPE) and neural responses to physical effort. The design offers several strengths, including high ecological validity (31), the successful implementation of three effort intensities, and a rigorous multi-method approach to capture different facets of effort valuation. Contrary to our hypotheses, eight weeks of jump training did not yield reliable evidence for changes in state VoPE, vmPFC responses during exertion, or voluntary exerted effort in a transfer task. These findings suggest that VoPE may be less readily modified than anticipated. Interestingly, although the TG reported generally higher state VoPE than the CG in the free ride task, ANCOVA controlling for baseline VoPE indicated that this difference was independent of the training. This reinforces the notion that VoPE may be relatively stable, particularly when far-transfer is tested.* (pp. 33-34)**

**“*In summary, this study provides initial insights into whether regular physical exercise can influence the value of physical effort (VoPE). Contrary to our expectations, eight weeks of high-intensity jump training did not alter participants’ willingness to exert effort, their self-reported VoPE, or neural responses in the vmPFC associated with effort valuation. While these findings point to a relative stability of VoPE, they may also reflect specific characteristics of the intervention, such as its high training intensity and the absence of systematic rewards, which could have confounded potential effects on effort valuation. Future research should investigate potential moderating factors, such as the timing of VoPE assessment, the role of intrinsic and extrinsic rewards, or developmental sensitivity, to better understand the conditions under which VoPE might be modifiable.*” (p. 39)**

b. **The point that high intensity may be less beneficial than low or moderate intensity is valuable. However, no moderation analysis was performed – e.g. whether individual tolerance to effort (VO₂max, previous physical activity) influenced the results. Such an analysis could provide important insights into who benefits most from the intervention.**

We thank the reviewer for this valuable and comment. This point highlights an important perspective on how training intensity may interact with individual differences in effort tolerance. Due to the relatively small and highly selective nature of the sample (e.g., one of inclusion criteria being low physical activity), there was limited variability and statistical power to reliably examine potential moderating effects. However, we have now incorporated this consideration into the Discussion, where we outline how future studies could examine factors such as cardiorespiratory fitness or prior physical activity levels to better understand who may benefit most from interventions of varying intensity. We believe that this addition provides a more nuanced interpretation of our findings and helps to guide future research in this area.

**“*Several characteristics of the intervention provide useful starting points for explaining the absence of learning-related changes. First, the training protocol was considerably more intense than in earlier studies (31,76). While we initially assumed that high-intensity training would provide a strong learning signal, lower-intensity training may offer a more favorable context for effort-based learning. Our findings suggest that the aversive nature of intense effort (36,37) could attenuate the development of positive effort valuation. Importantly, the effectiveness of a given training intensity may depend on individual differences such as preference and tolerance to physical effort (77), cardiorespiratory fitness, or prior levels of physical activity. Future research should therefore systematically examine how different intensities interact with individual characteristics to shape VoPE learning, and to identify which individuals benefit most from specific training intensities.*” (pp. 34-35)**

c. **The authors refer to the theory of Learned Industriousness and attribute the lack of effect to the absence of rewards. This is a coherent interpretation, but the question remains whether a sense of competence (e.g. improvements in fitness tests) may not have been sufficiently emphasised. The absence of monitoring participants’ perception of progress is a significant limitation.**

Thank you for this valuable comment. We agree that, within the framework of Learned Industriousness, perceived competence and progress may act as reinforcers inherent to the task. As noted, the present study did not assess participants’ perception of progress, which limits conclusions regarding whether effort was effectively reinforced.

We now acknowledge this limitation explicitly in the Discussion and note that future studies should monitor perceived progress and competence to better evaluate reinforcement mechanisms and learning effects.

**“*Moreover, according to the theory of Learned Industriousness (30), effort must be paired with rewards to reduce its subjective costs and potentially enhance its value. Such reinforcement can be inherent to the task itself or provided externally. In the context of physical training, inherent rewards may include improvements in physical fitness, weight loss, or body toning. Previous studies reporting decreased perceived effort costs and increased industriousness without explicit external reinforcement (31,76) may therefore have benefited from such inherent rewards. Based on these findings, and to avoid interference with other aspects of the research project, we refrained from implementing external rewards, expecting the training itself to be sufficiently rewarding. However, our findings suggest that this may not have held true. Participants exerted substantial effort, yet experienced little objectively noticeable improvements (58), which may have weakened reinforcement and limited increases in VoPE to a transfer task. Importantly, participants’ perception of progress or sense of competence were not assessed, which might have varied between participants even in the absence of measurable gains.*** ***This represents a methodological limitation that constrains interpretations regarding whether reinforcement via inherent rewards contributed to learning-related mechanisms. Interestingly, we did not observe a decrease in VoPE either, indicating that unrewarded effort neither enhances nor diminishes VoPE in a transfer task. Although previous studies also lacked explicit external rewards (31,76), the considerably higher effort intensity of our training protocol might have contributed to the different results. Given that higher efforts are typically associated with more negative affect (36,37) and thus greater inherent costs, the lack of adequate reinforcement following such strenuous effort may have further limited learning-related changes in VoPE. However, evidence from the cognitive domain is mixed regarding whether secondary reinforcement alone can effectively alter effort valuation (32,35). To test whether consistent pairing of effort and reward is necessary for VoPE learning, future studies could implement structured reinforcement strategies, such as increasing awareness of progress through fitness assessments, or explicit feedback, or by providing rewards proportionally to exertion rather than outcomes. This would offer a more rigorous test of Learned Industriousness in the domain of physical effort.*” (pp. 35-36)**

d. **The discussion that VoPE ratings during effort may differ from retrospective evaluation is very valuable. However, the study lacks even a single retrospective measure (e.g. a post-training-cycle questionnaire). This omission prevents verification of one of the authors’ key hypotheses.**

Thank you for this valuable comment concerning the temporal aspects of effort valuation. The aim of our study was to examine whether the regular engagement in physical effort can increase VoPE during the exertion itself. Our research question did not address whether effort is perceived more positively after completion of exertion (i.e., retrospective valuation). Instead, we specifically focused on valuation while effort is being exerted, as this may be directly relevant for promoting sustained physical activity behavior (Maltagliati et al., 2024; <https://doi.org/10.51224/SRXIV.375>). A retrospective assessment would therefore constitute an interesting but distinct research question.

Our methodological choices reflect this temporal focus: in addition to collecting subjective state VoPE ratings during exertion, we also measured neural activity during physical effort. In contrast, studies examining retrospective evaluations of effort typically focus their neural measurements on the immediate post-exertion phase (Jiang & Zheng, 2023; Wu & Zheng, 2023).

Furthermore, theoretical distinctions regarding the timing of effort valuation (i.e., prospective, concurrent, and retrospective) were elaborated more clearly in the literature only after data collection for the present study had been completed (Cheval et al., 2025). At the time of study planning, these distinctions were discussed only more broadly (Inzlicht et al., 2018).

We have now clarified this temporal sequence in the revised manuscript to ensure that readers understand why retrospective measures were not included and how our approach aligns with our research aims. We also agree that investigating retrospective valuation constitutes an interesting avenue for future research and have highlighted this in the revised discussion.

Finally, we have revised the wording of our hypotheses to more clearly articulate the temporal focus of our study.

“***Another factor may lie in the timing of state VoPE assessment. Theories on effort suggest that individuals are more likely to value effort retrospectively once it has been completed, rather than during the effort (12,29). Effort might be processed differently before, during, and after an activity (29). In particular, during physical exertion, individuals are often focused on performance optimization and minimizing discomfort (23,29). In contrast, retrospective evaluations may allow for a more reflective and positively biased appraisal of the experience (29). Notably, these conceptual distinctions were articulated more clearly in the literature only after data collection for the present study had been completed, whereas the theoretical landscape at the time of study planning was less differentiated. Accordingly, VoPE was assessed concurrently during active exertion in a separate laboratory task, and participants were not asked to retrospectively evaluate the effort involved in the eight-week training itself. Future studies could benefit from assessing VoPE at multiple times, such as before, during, and after an intervention, to more accurately capture the temporal dynamics of effort valuation and potential learning effects (29).* “ (p. 37)**

**“*We hypothesized that (a) state VoPE during exercise would increase in the TG, potentially varying by effort intensity, (b) vmPFC oxygenation during cycling would increase in the TG, with no changes in preSMA (45), and (c) voluntarily exertion (power output and heart rate) and RPE would increase in the TG, but not in the control group (CG).*” (p. 11)**

e. **The observation that young people may be more susceptible to VoPE modification opens an interesting research avenue. Nevertheless, potential sex, cultural or personality differences (e.g. temperamental traits) that may modulate the learning of effort value should also be discussed.**

We appreciate this insightful comment. We have now incorporated this important consideration into the Discussion and believe that it has enriched the manuscript by highlighting additional factors that may modulate the learning and modification of effort valuation. (see also our answer to you comment 1b)

**“Another possible explanation lies in the developmental sensitivity of learning VoPE (20). Adolescents appear to be less sensitive to effort costs and exert more effort relative to task demands and rewards at stake than adults (78,79). This period of heightened responsiveness may facilitate the learning of the relationship between effort and reward, making it easier to internalize the contingency that greater effort leads to greater rewards. By contrast, by the time individuals reach early adulthood, as in the current sample, it may be more challenging to relearn or modify their VoPE. While this observation opens an interesting avenue for developmental research, it is also important to consider potential moderating factors such as previous learning experiences in the sport context, or genetic predispositions (e.g., differences in the dopaminergic reward system) that may influence how VoPE is learned. Future research should therefore test similar interventions with adolescent samples and systematically examine individual differences that may shape sensitivity to learning VoPE*.*” (pp. 37-38)**

f. **The authors rightly note that fNIRS is limited to cortical surface areas and does not capture subcortical structures (e.g. nucleus accumbens). However, their conclusion that no effect was observed “neuronally” is too far-reaching. It should be made clear that the findings refer only to vmPFC and preSMA activity, not the entire reward or effort network.**

Thank you very much for this important and valuable comment. We fully agree with this point and revised the Conclusion accordingly:

**„*In summary, this study provides initial insights into whether regular physical exercise can influence the value of physical effort (VoPE). Contrary to our expectations, eight weeks of high-intensity jump training did not alter participants’ willingness to exert effort, their self-reported VoPE, or neural responses in the vmPFC associated with effort valuation.*“ (p. 39)**

**Yours sincerely,**

Thank you very much for your positive and encouraging feedback. We were particularly impressed by the depth and thoroughness of your review and are sincerely grateful for your constructive suggestions and thoughtful remarks. We have carefully considered and incorporated your comments into the revised manuscript and hope that the changes adequately address your points.

------------------------------------------------ comments Reviewer #3 ------------------------------------------

**The topic of the study is relevant, the results are interesting to the reader, and the manuscript is generally well written.**

**However, in my opinion, the text contains an excess of detail and unnecessary information, which forces the reader to go back and forth to fully understand the context. A manuscript exceeding 20 pages becomes difficult to follow. I strongly recommend that the authors summarize and streamline the content to improve readability and focus on the main findings.**

Thank you very much for this feedback. In response, we have carefully shortened and streamlined the entire manuscript to improve clarity and focus.

**1. For example: The Introduction consists of ten paragraphs, which is considerably more than the average research paper. Several of these paragraphs serve as a literature review rather than a justification for the study.**

Thank you for raising this helpful comment. We have shortened the Introduction about 50% from 6 pages to 3 and streamlined the information presented. We believe these revisions improved the clarity and focus of the manuscript, and we appreciate the suggestion to make this section more concise.

**2. The subsection titled Present Research should not appear within the Introduction; instead, it should be incorporated succinctly in the Methods section.**

Thank you for this comment. In line with your suggestion, we have now removed this subsection from the Introduction and integrated the relevant content into the Methods section.

See for example:

**“*This study was part of the research project x and approved by the ethics committee of the x (ref. 31/2022). Participants provided written informed consent prior to participation. We investigated whether regular physical effort alters individuals’ VoPE and neural responses to physical effort, or whether VoPE remains relatively stable over time. Participants in the training group (TG) completed a multi-week jump training protocol, and VoPE was assessed pre/post using two cycling tasks, allowing us to test far-transfer effects across task domains (30,32). Subjective, neural, and behavioral measures of VoPE were collected (20). We hypothesized that (a) state VoPE during exercise would increase in the TG, potentially varying by effort intensity, (b) vmPFC oxygenation during cycling would increase in the TG, with no changes in preSMA (45), and (c) voluntarily exertion (power output and heart rate) and RPE would increase in the TG, but not in the control group (CG).*” (pp. 10-11)**

**3. The description of the experimental protocol is excessively long and could be condensed.**

Thank you for this suggestion, which we have gladly implemented (see also Reviewer #1, comment 2). We have removed secondary information, reducing the text by about 20% (approx. 1.5 pages), while retaining all relevant content, making the description of the procedure even clearer.

**4. The Data Analysis section extends to six paragraphs, followed by further detailed descriptions of data processing for each signal, which is again overly elaborate.**

Thank you for this helpful comment. As our dataset is quite extensive (including fNIRS and cardiovascular measures), a transparent description of the preprocessing and analysis procedures is somewhat longer than in many other studies without such data. We retained the detailed description of the fNIRS preprocessing steps in response to Reviewer #1’s comment 3d. Nevertheless, we have now streamlined and substantially shortened (from 6 to 3 paragraphs) the data analysis section to present our approach more clearly and coherently.

**5. In Table 1, the first two variables present means and standard deviations as rounded numbers. Please ensure that all variables use the same number of decimal places.**

Thank you for this suggestion. In response, we have now rounded all values in the table to two decimal places (p. 23)

**6. Each results table is followed by three to four paragraphs of explanation, which is too extensive for a single table.**

Thank you for pointing this out. In response we have substantially shortened the Results section, reducing it by approximately 600 words while retaining all essential statistical information. We believe that this revision improves the clarity and conciseness of the manuscript.

**In summary, my comments aim to encourage the reduction of redundant and irrelevant information to help maintain the reader’s attention on the study’s key findings and contributions.**

Thank you for your positive and helpful feedback. We greatly appreciate your suggestions, and we have made several revisions to improve our manuscript. Most importantly, we have shortened the manuscript to about 20 pages, as suggested, and we believe that this improves its clarity and readability.

------------------------------------------------ comments Reviewer #4 ------------------------------------------

**This paper looks at whether an 8-week high-intensity jump training program can change how people value physical effort and their willingness to try hard. This is an important question, and the study is ambitious, with strengths like a randomized controlled design, real-world relevance, multiple assessment methods (self-report, behavior, and fNIRS), high participation, and open data access. The paper is well-written, and the discussion offers useful insights about no significant findings.
However, several key issues need major changes before the paper can be published. These issues affect how we understand and trust the main conclusions.**

Thank you very much for your constructive and thoughtful comments. We are pleased that the strengths of our study and manuscript were recognized, and we sincerely appreciate your helpful suggestions for further improvement. We have carefully considered your feedback and have incorporated the proposed changes into the revised manuscript. **1. Key Issues Needing Major Changes
Inconsistency in Study Design Reasoning
The paper mentions that high-intensity effort is often unpleasant. Despite this, the study uses a high-intensity program as the main intervention. This is inconsistent and might have reduced the chance of seeing positive changes. This choice must be clearly explained in the introduction, and the risks and challenges should be discussed openly.**

Thank you for this thoughtful comment. We agree that high-intensity physical effort is often perceived as aversive and that this may pose challenges for observing positive changes in effort valuation. We have therefore revised the Introduction to more clearly explain our rationale for choosing a high-intensity intervention. Specifically, we now argue that the greater aversiveness of high-intensity exercise may provide a stronger basis for changes in effort valuation, given evidence that such intensities engage neurophysiological mechanisms related to reward and affect regulation, including the endogenous opioid and endocannabinoid systems (see also answer to Reviewer #1, comment 1c).

**“*In Bernacer et al. (31), initially inactive participants completed a three-month low-intensity exercise program, after which effort costs had less influence on effort-based decisions, accompanied by decreased effort-related activity in the anterior cingulate cortex (ACC) (31). These findings suggest that regular exercise may reduce effort costs and alter neural responses to effort (31). However, as effort intensity was low and higher intensities are often valued less (26) and perceived as more aversive (39–41), it remains unclear whether training at higher intensities, might yield stronger effects on effort valuation. Notably, high-intensity exercise may engages additional neurophysiological mechanisms, such as increased activation of the endogenous opioid and endocannabinoid systems, which have been linked to affective responses and reward processing during intense exertion (39,40). Thus, while previous research has begun to explore near-transfer effects in decision-making (31), it remains largely unclear whether regular physical effort alters how effort is valued (rather than its costs) during actual exertion, whether such changes generalize across tasks, and how they depend on exercise intensity (20).*” (pp. 6-7)

2. fNIRS Data Processing Concerns
The authors used "less strict" methods to correct motion artifacts. This increases the risk of noise in the neural data, which might hide real effects. The authors should either:
Provide extra analyses with stricter filtering to show the results are strong, or
Be cautious in interpreting the neural findings, acknowledging that the results might be unreliable due to noise.**

We truly appreciate this insightful comment. We agree that the previous description of our motion artifact correction was unclear. In fact, we applied a processing pipeline employing methods that vary in strictness and methodological angle, enabling sensible noise removal without risking of filtering out the neural signal altogether.

For the first step, spline interpolation, some parameters were set relatively liberally to accommodate the high movement nature of the cycling task. In a subsequent step, we applied wavelet motion correction with a stricter-than-usual IQR (1.0 vs. the more common 1.5), counterbalancing the preceding liberal spline interpolation. We now state this rationale more clearly in the paper as we did not provide sufficient information before.

**„*Motion artifacts were addressed using a hybrid approach (67) employing methods that vary in strictness and methodological angle, enabling sensible noise removal without risking filtering out the neural signal altogether. Artifacts were first detected with hmrR_MotionArtifactByChannel (tMotion = 0.5, tMask = 1.5, STDEVthresh = 50.0, AMPthresh = 0.20) and corrected using hmrR_MotionCorrectSpline (p = 0.99), using relatively liberal parameters appropriate for the high-movement cycling task. Subsequently, hmrR_MotionCorrectWavelet was applied with a stricter-than-usual IQR (Iqr = 1.0 vs. typical ~1.5) to attenuate residual motion spikes, counterbalancing the preceding liberal settings.*“ (p. 18)**

**3. Baseline Group Differences in VoPE Not Addressed
The training group had higher VoPE than the control group at the start in the free ride task. This is a major issue that affects the fairness of the randomization. The authors need to:
Clearly show baseline differences,
Use statistical methods (e.g., ANCOVA) with baseline VoPE as a factor, and
Discuss how this affects the idea that VoPE is "stable."**

We thank the reviewer for pointing this out. We have addressed baseline differences in the free ride task by conducting a Bayesian ANCOVA with baseline VoPE as a covariate. This analysis showed that condition did not explain variance beyond baseline VoPE, indicating that the observed differences in post-training VoPE were largely accounted for by initial baseline differences. We have added this analysis to the Results section and discussed it in the Discussion.

**„*To account for baseline differences in VoPE, we conducted a Bayesian ANCOVA with baseline VoPE as covariate. Model comparison favored the model including only baseline VoPE, BF01 = 6.37x10-11, (P(M/data) =.64), over the model including baseline and condition, BF01 = 0.316, P(M/data =.36). Effect inclusion revealed evidence for excluding condition as additional predictor, BFexcl = 1.74, P(excl/data) = 0.635, indicating that condition did not explain variance beyond baseline VoPE.*“ (p. 33)**

**“These findings suggest that VoPE may be less readily modified than anticipated. Interestingly, although the TG reported generally higher state VoPE than the CG in the free ride task, ANCOVA controlling for baseline VoPE indicated that this difference was independent of the training. This reinforces the notion that VoPE may be relatively stable, particularly when far-transfer is tested.” (pp. 33-34)

4. Intervention Fails to Produce Overall Fitness Improvement
While jump height improved, VO₂peak and general fitness did not. This suggests the program might not have been strong enough to change higher-level motivation like VoPE. This should be seen not just as a lack of reinforcement but also as a possible failure of the program's strength.**

Thank you for this insightful comment. We agree that the lack of change in general fitness highlights a potential limitation in the intensity or scope of the training program. While our intervention effectively improved jumping ability, it may not have been sufficiently demanding to elicit broader physiological adaptations that could influence higher-level motivational processes such as VoPE. Consequently, the absence of VoPE increases may reflect not only limited reinforcement but also the strength of the training itself. We have now highlighted this consideration in the Discussion as an additional limitation.

“***Second, despite the high intensity of the training protocol, its overall training effect was limited (55). Although jump performance improved, general fitness did not increase, suggesting that the training might not have been sufficiently effective to induce broader changes affecting higher-order motivational processes such as VoPE.*” (p. 35)

In summary, while this study makes an important attempt to test the malleability of VoPE, the above concerns must be resolved before the conclusions can be considered robust. Addressing these issues will require additional analyses, reframing of theoretical justification, and methodological clarification. Therefore, I recommend Major Revision.**

Thank you very much for your constructive and valuable feedback. We have carefully addressed all comments and are confident that our manuscript has substantially improved as a result.
