# Supplementary material for: An 8-week jump training did not boost effort value or the willingness to exert effort in a student sample
Source: PLoS One. 2026 Jul 16;21(7):e0352897. doi: 10.1371/journal.pone.0352897 (PMC13375129; doi:10.1371/journal.pone.0352897)
Supplement: Attachment — Submitted filename: Response_to_Reviewers_auresp_2.docx [file pone.0352897.s003.docx]

**Point-by-point reply**

**Manuscript number: #PONE-D-25-42417**

**Article title: An 8-week jump training did not boost effort value or the willingness to exert effort in a student sample**

--------------------------------------------- comments Christopher Kirk ----------------------------------------

**Dear XXX,**

**Thank you for submitting your manuscript to PLOS ONE. After careful consideration, we feel that it has merit but does not fully meet PLOS ONE’s publication criteria as it currently stands. Therefore, we invite you to submit a revised version of the manuscript that addresses the points raised during the review process.**

**Please submit your revised manuscript by Apr 20 2026 11:59PM. If you will need more time than this to complete your revisions, please reply to this message or contact the journal office at plosone@plos.org. When you're ready to submit your revision, log on to https://www.editorialmanager.com/pone/ and select the 'Submissions Needing Revision' folder to locate your manuscript file.**

**Please include the following items when submitting your revised manuscript:**

- **A letter that responds to each point raised by the academic editor and reviewer(s). You should upload this letter as a separate file labeled 'Response to Reviewers'.**
- **A marked-up copy of your manuscript that highlights changes made to the original version. You should upload this as a separate file labeled 'Revised Manuscript with Track Changes'.**
- **An unmarked version of your revised paper without tracked changes. You should upload this as a separate file labeled 'Manuscript'.**

**If you would like to make changes to your financial disclosure, please include your updated statement in your cover letter. Guidelines for resubmitting your figure files are available below the reviewer comments at the end of this letter.**

**If applicable, we recommend that you deposit your laboratory protocols in protocols.io to enhance the reproducibility of your results. Protocols.io assigns your protocol its own identifier (DOI) so that it can be cited independently in the future. For instructions see: https://journals.plos.org/plosone/s/submission-guidelines#loc-laboratory-protocols. Additionally, PLOS ONE offers an option for publishing peer-reviewed Lab Protocol articles, which describe protocols hosted on protocols.io. Read more information on sharing protocols at https://plos.org/protocols?utm_medium=editorial-email&utm_source=authorletters&utm_campaign=protocols.**

**We look forward to receiving your revised manuscript.**

**Kind regards,**

**Christopher Kirk, PhD**

**Academic Editor**

**PLOS One**

**Journal Requirements:**

**1. If the reviewer comments include a recommendation to cite specific previously published works, please review and evaluate these publications to determine whether they are relevant and should be cited. There is no requirement to cite these works unless the editor has indicated otherwise.**

**Additional Editor Comments:**

**Two reviewers are asking for relatively simple amendments to the structure of the manuscript, and specific details regarding the interpretation of the reported effect sizes. The third reviewer has highlighted some more detailed requirements about how of the variables and measurement methods have been discussed and interpreted.**

We sincerely thank you for your continued constructive and helpful feedback. We have revised the manuscript accordingly and improved the overall structure of the Introduction to enhance clarity and readability. In addition, we now report and more carefully interpret effect sizes.

Furthermore, we reprocessed the fNIRS data using an alternative preprocessing pipeline to assess the robustness of our findings. The results remained consistent, supporting the stability of our results. We have also strengthened the discussion by placing the findings more clearly within the broader context of the study.

Lastly, we reviewed the abstract to make it clearer, remove redundancies, and improve readability.

“***Physical inactivity remains highly prevalent, partly driven by the aversive nature of effort. However, effort can also be experienced as rewarding, which is associated with greater overall physical activity. Accordingly, the present study investigates whether regular physical exercise alters the self-reported value of physical effort, neural activation during exercise, and the willingness to exert effort. Sixty-two young adults were assigned to either an eight-week high-intensity jump training or a control group. Participants completed a two-task cycling ergometer exercise before and after the intervention. The first task assessed the value of effort at three pre-determined intensity levels, whereas the second task allowed participants to self-select the intensity to measure their willingness to exert effort. Participants’ perceived exertion and state value of physical effort were assessed, along with neural activation in the ventromedial prefrontal cortex and pre-supplementary motor area using functional near-infrared spectroscopy. Bayesian analyses provided evidence against main effects of condition, time, as well as their interaction, for self-reported value of physical effort, willingness to exert effort, and neural activity. This suggests that the value of effort may be relatively stable, as an eight-week training intervention did not alter the value of physical effort or the willingness to exert effort.***” (p. 3)

We believe that these revisions have again improved the manuscript and appreciate the opportunity to refine our work.

-------------------------------------------------- comments Reviewer #1 ----------------------------------------

**In the section on statistical analyses, the authors state that ‘Effect sizes were calculated to quantify the strength of any observed effects using Cohen’s d along with corresponding 95% confidence intervals’. However, they do not specify which effect size magnitudes these calculations refer to, nor which interpretative thresholds were applied. This constitutes a methodological shortcoming, as there exists a broad spectrum of effect size magnitudes, and without explicit criteria it is unclear how the authors interpret their results. For this reason, I suggested providing concrete thresholds. Specifically, for Cohen’s d, the following cut-offs should be used: 0.1, 0.4, and 0.8, respectively, with the corresponding citation (DOI: 10.1016/j.apmr.2025.05.013). Additionally, for ANOVA, the following thresholds should be reported: Small: W ≥ 0.1, Medium: W ≥ 0.3, Large: W ≥ 0.5, with appropriate citation (DOI: 10.11613/BM.2026.010101). According to these guidelines, the analysis should have been conducted accordingly; however, this information is still absent from the manuscript. Yours sincerely**

We thank the reviewer for this suggestion. However, we followed the conventional thresholds for Cohen’s d (0.2 = small, 0.5 = medium, 0.8 = large) as originally proposed by Cohen (1988), which remain the most widely used and broadly accepted benchmark across disciplines.

For ANOVA effect sizes, we used ω² instead of Kendall’s W, which is more appropriate for variance-based effect size estimation in mixed ANOVA designs.

***“In addition to Bayesian analyses, effect sizes were calculated to quantify the magnitude of observed effects. For ANOVA effects, ω² was reported (0.01 = small, 0.06 = medium, 0.14 = large) (50). For pairwise comparisons, Cohen’s d was reported (0.2 = small, 0.5 = medium, 0.8 = large), along with corresponding 95% confidence intervals (50).” (p. 21)***

Results – see for example:

***“The best model including the Time x Condition interaction, also included main effects of Time, Condition, and Interval, BF10 = 4.98, P(M/data) = .04, ω² = 0.017 (for complete results tables, see OSF), providing substantial evidence for the alternative hypothesis (51).” (p. 26)***

------------------------------------------------ comments Reviewer #3 ------------------------------------------

**I appreciate the effort the authors have made in revising the manuscript. The text is now considerably clearer and more pleasant to read.**

**In this review, I will focus primarily on the revisions made in response to my initial comments, as I recognize that several additional modifications were implemented to address remarks from other reviewers.**

Thank you very much for this helpful feedback. We have addressed the comments accordingly and believe that these revisions have further improved our manuscript.

1. **Introduction: Although the section has been substantially reduced, the inclusion of subtopics within the introduction is not standard practice. I recommend restructuring this section to present a more direct and straightforward justification of the study.**

Thank you for this remark. We have revised the introduction so that separate subtopics have been integrated into the main text. The section has also been streamlined and more clearly focused on the present study, providing a more direct justification of our research. (See pp. 4-11)

2. **Objectives: The objectives are currently presented as a justification for the study rather than as clear statements. I suggest rewriting them in an affirmative and explicit manner.**

Thank you for this helpful suggestion. The objectives are now presented more explicitly and in an affirmative manner. Additionally, the study aims are now stated more clearly to more directly outline the purpose of the study.

***“The aim of the present study therefore was to examine whether regular physical exercise alters the value of physical effort during actual physical exertion. Specifically, we investigated whether changes in subjective, behavioral and neural indicators of effort valuation generalized beyond the training task.” (p. 9)***

3. **Conclusions: The conclusions refer only to VoPE. It would be appropriate to also incorporate the other variables examined in the study to provide a more comprehensive summary of the findings.**

We appreciate this helpful suggestion and revised the conclusion to reflect the broader set of variables. In particular, we now explicitly refer to the behavioral and neural measures alongside VoPE to provide a more comprehensive summary of the study’s findings. (See also our response to Reviewer 4, comment #4)

***“In summary, this study provides initial insights into whether regular physical exercise can influence the valuation of physical effort during exercise across behavioral, subjective, and neural levels. Contrary to our expectations, eight weeks of high-intensity jump training did not alter participants’ willingness to exert effort, their self-reported value of physical effort (VoPE), or neural responses in the vmPFC associated with effort valuation. Together, these findings suggest a relative stability of concurrent effort valuation in the context of the present training paradigm. Future research should therefore examine whether different training characteristics (e.g., training intensity), timing of VoPE assessment, the role of intrinsic and extrinsic rewards, or other populations (e.g., active individuals, adolescents), influence the modifiability of effort valuation.” (p. 41)***

**References: Approximately 87 references appear excessive for a single article. I recommend reducing the number to around 60, ensuring that only the most relevant and essential sources are retained.**

Thank you for this suggestion. We have carefully reviewed the references and reduced the number of citations to 61 by removing less essential and redundant references while retaining the most relevant sources necessary to support the argumentation and theoretical background of the manuscript.

------------------------------------------------ comments Reviewer #4 ------------------------------------------

**Thanks to you, your work has been revised. The manuscript is improved; however, there are still several critical points that should be corrected to be published.**

**1. fNIRS Data Robustness (High Priority) you failed to perform the sensitivity analysis with more stringent motor-correction parameters. Signal quality aspects such as percent rejected channels, SNR or channel acceptance rates were not reported. Findings of neural nulls are not explained well in the light of potential motion artifact.**

**Required: Conduct more severe settings sensitivity analysis, report signal quality metrics, and talk more about motion related limitations.**

We thank the reviewer for this important and constructive comment. To assess robustness of the present results against different preprocessing choices, we preprocessed the data with another processing stream that has been used in prior research that employed fNIRS in the context of a physical effort task (see, doi: 10.1038/s41598-018-34009-2). This did not change our results. If the editor wishes, we would be happy to provide these additional analyses as supplementary material.

enPruneChannels function: remove channels when the signal was too weak or too strong

Intensity_to_OD: optical intensity converted to optical density

Wavelet_Motion_Correction: remove motion artifacts (IQR of 1).

low pass filter (0.5 Hz)

converted to oxy- and deoxyhemoglobin with the modified Beer-Lambert law

Furthermore, we have now included explicit reporting of signal quality metrics in the Method section:

***“Overall, 6.3% of channels were rejected due to insufficient signal quality. Of these rejected channels, 29% were excluded automatically by the preprocessing stream, while the remaining 71% were identified and removed through manual inspection.” (p. 19)***

Finally, we have expanded the Discussion to more explicitly address the potential impact of motion-related artifacts:

***“Finally, the current study assessed neural activation during periods of (partially) intense physical exertion, which offers valuable ecological validity. However, although fNIRS is relatively robust to motion artifacts (56), it is important to note that motion-related noise cannot be fully excluded, particularly in the context of physical demanding tasks such as a cycling task. Despite careful preprocessing and quality control procedures, residual motion artifacts may have influenced the signal and could have contributed to the observed null findings. Importantly, however, no corresponding effects were observed at the behavioral or subjective level, suggesting that the absence of neural differences is consistent across multiple levels of analysis and not solely attributable to measurement noise.” (p. 39)***

**2. Multiple Comparisons (High Priority) You compared a large number of behavioral and neural outcomes and at various levels and times. You have not mentioned whether you have made adjustments to several comparisons. The risk management of Type I errors is not clear.**

**Note: indicate whether you used multiplicity corrections. Otherwise, justify and distinguish between confirmatory and exploratory analysis.**

Thank you for this important comment. We agree that the large number of comparisons requires careful consideration and transparent reporting.

All primary analyses were conducted within a Bayesian framework, in which multiplicity corrections are not required (e.g., 10.1080/19345747.2011.618213), as Bayes factors quantify the relative evidence for each model given the observed data, rather than relying on a repeated-sampling logic that necessitates control of Type I error rates (10.3758/s13423-017-1343-3). Nevertheless, we acknowledge that conducting multiple analyses increases the risk of overinterpretation. To further strengthen the interpretability of our findings and guard against over-interpretation, we now explicitly label analyses as confirmatory or exploratory in the Methods section.

***“For state VoPE and cerebral oxygenation (oxygenated (HbO) and de-oxygenated (HbR), hemoglobin concentration) of the interval task, mean values of the second minute of the ride were analyzed using the same 2-between (condition: TG, CG) x 3-within (intensity levels: low, moderate, vigorous) x 2-within (time: pre- and post-training) Bayesian repeated measures ANOVA design in JASP. For the free ride task, 2-between (condition: TG, CG) x 2-within (time: pre- and post-training) Bayesian repeated measures ANOVAs were conducted for heart rate (10-minute mean), RPE, rendered power (10-minute mean, normalized to the RCP), and state VoPE. All primary analyses were pre-specified, whereas post hoc analyses were considered exploratory and should be interpreted with appropriate caution given the number of comparisons.” (p. 21)***

An example of this clarification can be found in the revised Results section:

“***Exploratory post hoc comparisons indicated strong evidence that state VoPE ratings were lower during the vigorous interval (M = 1.26, SD = 2.13) compared to the low (M = 1.79, SD = 1.89), BF10 = 167.43, Oddspost = 98.35, d = 0.26, 95% CI [0.04, 0.48], and moderate intervals, BF10 = 140.77, Oddspost = 82.69, d = 0.23, 95% CI [0.04, 0.43], with both differences corresponding to small effect sizes.***” (p. 26)

**3. Baseline Transparency (High Priority) Your ANCOVA, but no CONSORT-type baseline equivalence table. Group comparability on demographics and baseline variables were not clearly reported.**

**Necessary: Provide a detailed and presented Table 1 with a demographic and baseline comparison, statistical tests, and effect sizes.**

To improve transparency, we created a table summarizing the demographic characteristics and baseline values of all analyzed variables for both conditions and tested whether the conditions differed on these variables at baseline.

Two significant baseline differences were identified. First, state VoPE in the Free Ride task differed, as already reported in the manuscript, t(262) = -3.930, p <.001, d = -0.46 [-0.68, 0.23]. Second, a baseline difference was observed for preSMA HbR values during the low-intensity interval, t(55) = 2.019, p = .048, d = 0.53 [0.00, 1.05], with stronger activation for the CG than the TG.

To examine whether these baseline differences influenced the main analyses, we conducted additional Bayesian ANCOVAs including the respective baseline values as covariates. For both variables, the analysis of effect inclusion indicated evidence for excluding condition as an additional predictor, suggesting that condition did not explain variance beyond baseline levels.

The ANCOVA results for state VoPE are already reported in the main test. To avoid unnecessarily increasing the manuscript length, the results of the ANCOVA for preSMA HbR are provided in the JASP analyses script available on OSF and are mentioned in the Table note. In addition, the full baseline table has been uploaded to OSF for transparency and again to avoid making the manuscript overly lengthy.

Note in the Results section of the manuscript:

“***Furthermore, baseline comparability between conditions was examined. Although baseline differences were observed for two variables (see S3), subsequent analyses indicated that these differences could not be attributed to condition.***” (p. 23)

Document “Baseline Table” in OSF „***Table 1***

***Means and standard deviations of the main variables per intensity interval and for the free ride task, separate for the training and control groups, and for the two measurement points.***

| Variable | TG (n = 34) | CG (n = 28) | Test | p | Effect size |
| --- | --- | --- | --- | --- | --- |
| **Age** | 22.85 ± 3.58 | 22.71 ± 2.42 | *t*(177) = -0.45 | .751 | *d* = -0.05 [-0.33, -0.24] |
| **Sex** | ♀ 50%, ♂ 50% | ♀ 54%, ♂ 46% | *χ²*(1) = 0.114 | .736 | *φ* = 0.04 |
| **Power [W]** |  |  |  |  |  |
| Low | 113.38 ± 29.62 | 102.79 ± 30.83 | *t*(57) = -1.370 | .176 | *d* = -0.35 [-0.85, 0.15] |
| Moderate | 156.83 ± 37.17 | 143.10 ± 38.69 | *t*(57) = -1.416 | .162 | *d* = -0.36 [-0.87, 0.14] |
| Vigorous | 190.13 ± 45.22 | 174.72 ± 43.64 | *t*(58) = -1.361 | .179 | *d* = -0.35 [-0.85, 0.16] |
| Free ride | 154.76 ± 45.89 | 145.61 ± 42.02 | *t*(299) = -1.808 | .072 | *d* = -0.21 [-0.43, 0.02] |
| **Heart rate**  **[min^-1^]** |  |  |  |  |  |
| Low | 146.47 ± 15.38 | 150.32 ± 15.16 | *t*(56) = 0.979 | .332 | *d* = 0.25 [-0.26, 0.76] |
| Moderate | 157.13 ± 13.34 | 159.39 ± 13.28 | *t*(54) = 0.654 | .516 | *d* = 0.17 [-0.34, 0.68] |
| Vigorous | 162.24 ± 12.07 | 164.89 ± 9.87 | *t*(59) = 0.941 | .350 | *d* = 0.24 [-0.27, 0.74] |
| Free ride | 160.00 ± 17.14 | 162.72 ± 18.20 | *t*(279) = 1.320 | .188 | *d* = 0.15 [-0.07, 0.38] |
| **RPE** |  |  |  |  |  |
| Low | 3.12 ± 1.39 | 3.18 ± 1.36 | *t*(58) = 0.174 | .863 | *d* = 0.04 [-0.46, 0.54] |
| Moderate | 4.18 ± 1.41 | 3.82 ± 1.12 | *t*(60) = -1.102 | .275 | *d* = -0.27 [-0.78, 0.23] |
| Vigorous | 5.38 ± 1.53 | 5.14 ± 1.26 | *t*(60) = -0.676 | .502 | *d* = -0.17 [-0.67, 0.33] |
| Free ride | 4.10 ± 1.79 | 3.99 ± 1.47 | *t*(308) = -0.579 | .563 | *d* = -0.06 [-0.29, 0.16] |
| **State VoPE** |  |  |  |  |  |
| Low | 2.15 ± 1.73 | 1.50 ± 1.93 | *t*(55) = -1.376 | .175 | *d* = -0.36 [-0.86, 0.15] |
| Moderate | 2.03 ± 2.05 | 1.36 ± 2.11 | *t*(57) = -1.263 | .212 | *d* = -0.32 [-0.83, 0.18] |
| Vigorous | 1.56 ± 1.80 | 1.00 ± 2.28 | *t*(51) = -1.056 | .296 | *d* = -0.28 [-0.78, 0.23] |
| Free ride | 2.11 ± 1.76 | 1.21 ± 2.22 | *t*(262) = -3.930 | **<.001** | *d* = -0.46 [-0.68, 0.23] |
| **preSMA HbO [µmol/l]** |  |  |  |  |  |
| Low | -0.09 ± 1.06 | 0.20 ± 1.58 | *t*(42) = 0.778 | .441 | *d* = 0.21 [-0.31, 0.73] |
| Moderate | -0.49 ± 0.76 | 0.27 ± 2.17 | *t*(30) = 1.689 | .102 | *d* = 0.48 [-0.04, 1.01] |
| Vigorous | -0.54 ± 0.82 | -0.38 ± 1.23 | *t*(42) = 0.589 | .559 | *d* = 0.16 [-0.36, 0.68] |
| **preSMA HbR [µmol/l]** |  |  |  |  |  |
| Low | 0.06 ± 0.54 | 0.34 ± 0.49 | *t*(55) = 2.019 | **.048** | *d* = 0.53 [0.00, 1.05] |
| Moderate | 0.27 ± 0.52 | 0.41 ± 0.82 | *t*(41) = 0.772 | .445 | *d* = 0.21 [-0.31, 0.73] |
| Vigorous | 0.34 ± 0.48 | 0.61 ± 0.63 | *t*(46) = 1.811 | .077 | *d* = 0.49 [-0.04, 1.02] |
| **vmPFC HbO [µmol/l]** |  |  |  |  |  |
| Low | 1.27 ± 3.81 | 0.42 ± 3.13 | *t*(56) = -0.925 | .359 | *d* = -0.24 [-0.76, 0.28] |
| Moderate | 1.09 ± 2.56 | 0.80 ± 2.46 | *t*(54) = -0.436 | .665 | *d* = -0.11 [-0.63, 0.40] |
| Vigorous | 0.59 ± 3.17 | 0.52 ± 2.85 | *t*(55) = -0.091 | .928 | *d* = -0.02 [-0.54, 0.49] |
| **vmPFC HbR [µmol/l]** |  |  |  |  |  |
| Low | 0.36 ± 1.04 | 0.29 ± 0.91 | *t*(56) = -0.281 | .780 | *d* = -0.07 [-0.57, 0.44] |
| Moderate | 0.72 ± 1.12 | 0.70 ± 1.09 | *t*(54) = -0.078 | .938 | *d* = -0.02 [-0.54, 0.50] |
| Vigorous | 0.81 ± 1.00 | 0.75 ± 0.63 | *t*(54) = -0.300 | .767 | *d* = -0.08 [-0.59, 0.44] |

***Note. RPE = Rating of Perceived Exertion, range 0 - 10; state VoPE = state Value of Physical Effort, range -5 - +5; preSMA = pre-supplementary motor area; vmPFC = ventromedial prefrontal cortex; HbO = oxygenated hemoglobin; HbR = deoxygenated hemoglobin; Baseline differences between conditions were observed for two variables (highlighted in bold). To account for these differences, Bayesian ANCOVAs were conducted with the baseline as a covariate to examine the effect of condition. For both variables, the analysis of effect inclusion indicated evidence for excluding condition as an additional predictor, suggesting that condition did not explain variance beyond the baseline. For state VoPE, see the main article. For preSMA HbR, see the JASP script.***“

**4. VoPE Stability (Medium Priority) Conclusions may be excessive. Boundary conditions are not clearly spelt out.**

**Limitations: Conclusions may be made only pertaining to: High-intensity, non-reinforced training, Young, low-active adults, Concurrent measurement paradigm.**

Thank you for this helpful comment. We have revised the conclusion to avoid overly general interpretations and to more clearly acknowledge that our findings pertain to the training paradigm used here. We have also adjusted the wording of the conclusion to better reflect these boundary conditions and to highlight directions for future research. (See also our response to Reviewer 2 comment #3).

***“In summary, this study provides initial insights into whether regular physical exercise can influence the valuation of physical effort during exercise across behavioral, subjective, and neural levels. Contrary to our expectations, eight weeks of high-intensity jump training did not alter participants’ willingness to exert effort, their self-reported value of physical effort (VoPE), or neural responses in the vmPFC associated with effort valuation. Together, these findings suggest a relative stability of concurrent effort valuation in the context of the present training paradigm. Future research should therefore examine whether different training characteristics (e.g., training intensity), timing of VoPE assessment, the role of intrinsic and extrinsic rewards, or other populations (e.g., active individuals, adolescents), influence the modifiability of effort valuation.” (p. 41)***

**5. Limitations of Moderation Analysis (Medium Priority) You failed to perform subgroup analyses or moderation. It is a limitation that is brought up but not emphasized.**

**Additional: Add a limitation paragraph explaining clearly why moderation could not be done, why (e.g., VO 2max, prior activity, effort tolerance) should be measured in the future studies.**

We appreciate this important comment and have expanded the discussion with the limitation of the absence of subgroup or moderation analyses. Specifically, we explain that the present study was primarily designed to test whether VoPE can be modified, and that the modest sample size did not provide sufficient statistical power to reliably detect moderation effects.

***“While this observation opens an interesting avenue for developmental research, it is also important to consider potential moderating factors such as previous learning experiences in the sport context, or genetic predispositions (e.g., differences in the dopaminergic reward system) that may influence how VoPE is learned. The present study was designed to test whether VoPE can be modified through regular physical exercise. Consequently, potential moderation or subgroup effects were not systematically examined. Moreover, given the modest sample size, the study was not sufficiently powered to reliably detect moderation effects. Future studies with lager sample sizes should therefore test similar interventions in adolescent samples and systematically examine individual differences that may shape sensitivity to learning VoPE. In addition, it may be informative to consider further variables such as physiological indicators (e.g., VO2max), prior physical activity levels, and individual differences in effort tolerance as potential additional moderators.” (p. 39)***

Thank you very much for your thoughtful and constructive comments. We have considered and incorporated your suggestions into the revised manuscript and hope that the changes adequately address your points.
