# Supplementary material for: An 8-week jump training did not boost effort value or the willingness to exert effort in a student sample
Source: PLoS One. 2026 Jul 16;21(7):e0352897. doi: 10.1371/journal.pone.0352897 (PMC13375129; doi:10.1371/journal.pone.0352897)
Supplement: Attachment — Submitted filename: Response_to_Reviewers_auresp_4.docx [file pone.0352897.s005.docx]

**Point-by-point reply**

**Manuscript number: #PONE-D-25-42417**

**Article title: An 8-week jump training did not boost effort value or the willingness to exert effort in a student sample**

--------------------------------------------- comments Christopher Kirk ----------------------------------------

**Dear Dr. XXX,**

**Thank you for submitting your manuscript to PLOS ONE. After careful consideration, we feel that it has merit but does not fully meet PLOS ONE’s publication criteria as it currently stands. Therefore, we invite you to submit a revised version of the manuscript that addresses the points raised during the review process.**

**The reviewers require one final point to be addressed: Required Revisions (Minor)**

**1. fNIRS sensitivity analysis**

**The authors provided signal quality data (6.3% channel rejection) and re-processed the data with a different preprocessing pipeline that yielded consistent results. A point of interest, however, you are specifically asked for an analysis of the sensitivity of the results to tighter motion-correction parameters (such as tighter IQR limits). This query has to be rectified.**

**REVISE: Perform requested sensitivity analysis using more stringent parameters and report the results, or, if the sensitivity analysis is not needed, justify briefly in manuscript why this is not needed.**

**Please either complete and report these analyses, or provide a justification as to why such an analysis would not be needed in this instance.**

**Please submit your revised manuscript by Jul 05 2026 11:59PM. If you will need more time than this to complete your revisions, please reply to this message or contact the journal office at plosone@plos.org. When you're ready to submit your revision, log on to https://www.editorialmanager.com/pone/ and select the 'Submissions Needing Revision' folder to locate your manuscript file.**

**Please include the following items when submitting your revised manuscript:**

- **A letter that responds to each point raised by the academic editor and reviewer(s). You should upload this letter as a separate file labeled 'Response to Reviewers'.**
- **A marked-up copy of your manuscript that highlights changes made to the original version. You should upload this as a separate file labeled 'Revised Manuscript with TrackChanges'.**
- **An unmarked version of your revised paper without tracked changes. You should upload this as a separate file labeled 'Manuscript'.**

**If you would like to make changes to your financial disclosure, please include your updated statement in your cover letter. Guidelines for resubmitting your figure files are available below the reviewer comments at the end of this letter.**

**If applicable, we recommend that you deposit your laboratory protocols in protocols.io to enhance the reproducibility of your results. Protocols.io assigns your protocol its own identifier (DOI) so that it can be cited independently in the future. For instructions see: https://journals.plos.org/plosone/s/submission-guidelines#loc-laboratory-protocols. Additionally, PLOS ONE offers an option for publishing peer-reviewed Lab Protocol articles, which describe**

**protocols hosted on protocols.io. Read more information on sharing protocols at https://plos.org/protocols?utm_medium=editorial-email&utm_source=authorletters&utm_campaign=protocols.**

**As the corresponding author, your ORCID iD is verified in the submission system and will appear in the published article. PLOS supports the use of ORCID, and we encourage all coauthors to register for an ORCID iD and use it as well. Please encourage your coauthors to verify their ORCID iD within the submission system before final acceptance, as unverified ORCID iDs will not appear in the published article. Only the individual author can complete the verification step; PLOS staff cannot verify ORCID iDs on behalf of authors.**

**We look forward to receiving your revised manuscript.**

**Kind regards,**

**Christopher Kirk, PhD, FCASES**

**Academic Editor**

**PLOS One**

**Journal Requirements:**

**1. If the reviewer comments include a recommendation to cite specific previously published works, please review and evaluate these publications to determine whether they are relevant and should be cited. There is no requirement to cite these works unless the editor has indicated otherwise.**

**2. Please review your reference list to ensure that it is complete and correct. If you have cited papers that have been retracted, please include the rationale for doing so in the manuscript text, or remove these references and replace them with relevant current references. Any changes to the reference list should be mentioned in the rebuttal letter that accompanies your revised manuscript. If you need to cite a retracted article, indicate the article’s retracted status in the References list and also include a citation and full reference for the retraction notice.**

We sincerely thank you for handling our manuscript and for the constructive and thoughtful feedback provided throughout the review process. We greatly appreciate the time and effort invested by you and the reviewers. We have carefully addressed all points raised. We also appreciate the fast handling of the review process.

------------------------------------------------ comments Reviewer #3 ------------------------------------------

**The article is much improved and clear.**

**I personally don't like paragraphs starting with 'however' (there are 2 in the introduction), It seems the idea is not complete. Besides that, no other recommendations.**

Thank you for this helpful suggestion. We agree that beginning paragraphs with “however” can make the transition appear somewhat abrupt. We therefore replaced these paragraph openings with smoother transitional phrasing to improve the overall flow and readability of the manuscript.

“***At the same time, accumulating evidence indicates that physical effort can simultaneously be perceived as valuable – at least to some degree, for some people, and in some situations (6,10).***” (p. 6)

“***This interpretation should be considered in light of the relatively low effort.***” (p. 7)

------------------------------------------------ comments Reviewer #4 ------------------------------------------

**Required Revisions (Minor)**

**1. fNIRS sensitivity analysis**

**The authors provided signal quality data (6.3% channel rejection) and re-processed the data with a different preprocessing pipeline that yielded consistent results. A point of interest, however, you are specifically asked for an analysis of the sensitivity of the results to tighter motion-correction parameters (such as tighter IQR limits). This query has to be rectified.**

**REVISE: Perform requested sensitivity analysis using more stringent parameters and report the results, or, if the sensitivity analysis is not needed, justify briefly in manuscript why this is not needed.**

Thank you for this important comment regarding the sensitivity of the findings to motion-correction choices.

In the primary preprocessing pipeline, we already employed stringent motion-correction procedures for a high-movement exercise paradigm. Specifically, the wavelet-based correction used an IQR threshold of 1.0, which is stricter than the commonly used threshold of 1.5 (Gao et al., 2022; Molavi & Dumont, 2012). At the same time, excessively aggressive denoising (such as further decreasing the IQR) may attenuate meaningful hemodynamic signal and increase the risk of overcorrection, particularly in physically demanding movement tasks (Brigadoi et al., 2014; Chiarelli er al., 2015).

We therefore believe that further increasing correction strictness would be unlikely to provide meaningful benefits while increasing the risk of overcorrection.

Nevertheless, to further evaluate the robustness of the findings and assess whether the results were dependent on the specific preprocessing choices applied in the primary pipeline, we undertook the additional effort of reprocessing the entire dataset using an alternative preprocessing stream. For this we used an appropriate processing stream that has been employed in previous physical exercise research, as reported in our previous revision.

To enhance transparency for readers, we now explicitly report this supplementary analysis and explicitly note that the observed effects were consistent across preprocessing pipelines, indicating that the findings were not driven by specific motion-correction parameter choices.

***“To assess the robustness of the findings with respect to preprocessing choices, the data were additionally processed using an alternative fNIRS preprocessing pipeline previously applied in physical effort paradigms (47). This alternative approach yielded highly similar results and did not change the overall pattern of findings, indicating that the reported results were not driven by specific preprocessing parameter choices.” (p. 19)***
