# Supplementary material for: An 8-week jump training did not boost effort value or the willingness to exert effort in a student sample
Source: PLoS One. 2026 Jul 16;21(7):e0352897. doi: 10.1371/journal.pone.0352897 (PMC13375129; doi:10.1371/journal.pone.0352897)
Supplement: Peer review history

# Peer review: Decision Letter 0

**DOI:** 10.1371/journal.pone.0352897.r001

**Authors:** Christopher Kirk

10 Nov 2025

PONE-D-25-42417

An 8-week jump training did not boost effort value or the willingness to exert effort in a student sample

PLOS ONE

Dear Dr. Stähler,

Thank you for submitting your manuscript to PLOS ONE. After careful consideration, we feel that it has merit but does not fully meet PLOS ONE’s publication criteria as it currently stands. Therefore, we invite you to submit a revised version of the manuscript that addresses the points raised during the review process.

Please submit your revised manuscript by Dec 25 2025 11:59PM. If you will need more time than this to complete your revisions, please reply to this message or contact the journal office at plosone@plos.org. When you're ready to submit your revision, log on to https://www.editorialmanager.com/pone/ and select the 'Submissions Needing Revision' folder to locate your manuscript file.

Please include the following items when submitting your revised manuscript:

- A rebuttal letter that responds to each point raised by the academic editor and reviewer(s). You should upload this letter as a separate file labeled 'Response to Reviewers'.
- A marked-up copy of your manuscript that highlights changes made to the original version. You should upload this as a separate file labeled 'Revised Manuscript with Track Changes'.
- An unmarked version of your revised paper without tracked changes. You should upload this as a separate file labeled 'Manuscript'.

If you would like to make changes to your financial disclosure, please include your updated statement in your cover letter. Guidelines for resubmitting your figure files are available below the reviewer comments at the end of this letter.

If applicable, we recommend that you deposit your laboratory protocols in protocols.io to enhance the reproducibility of your results. Protocols.io assigns your protocol its own identifier (DOI) so that it can be cited independently in the future. For instructions see: https://journals.plos.org/plosone/s/submission-guidelines#loc-laboratory-protocols. Additionally, PLOS ONE offers an option for publishing peer-reviewed Lab Protocol articles, which describe protocols hosted on protocols.io. Read more information on sharing protocols at https://plos.org/protocols?utm_medium=editorial-email&utm_source=authorletters&utm_campaign=protocols.

We look forward to receiving your revised manuscript.

Kind regards,

Christopher Kirk, PhD

Academic Editor

PLOS ONE

Journal Requirements:

When submitting your revision, we need you to address these additional requirements.

1.Please ensure that your manuscript meets PLOS ONE's style requirements, including those for file naming. The PLOS ONE style templates can be found at

https://journals.plos.org/plosone/s/file?id=wjVg/PLOSOne_formatting_sample_main_body.pdf and

https://journals.plos.org/plosone/s/file?id=ba62/PLOSOne_formatting_sample_title_authors_affiliations.pdf

2. Thank you for stating the following financial disclosure:

“The authors gratefully acknowledge funding from the Committee on Research (AFF) at the University of Konstanz for the research initiative "ProPELL: Promoting Physical Exercise in Lab and Life“”

Please state what role the funders took in the study.  If the funders had no role, please state: "The funders had no role in study design, data collection and analysis, decision to publish, or preparation of the manuscript."

If this statement is not correct you must amend it as needed.

Please include this amended Role of Funder statement in your cover letter; we will change the online submission form on your behalf.

3. We note that the grant information you provided in the ‘Funding Information’ and ‘Financial Disclosure’ sections do not match.

When you resubmit, please ensure that you provide the correct grant numbers for the awards you received for your study in the ‘Funding Information’ section.

4. Please note that funding information should not appear in any section or other areas of your manuscript. We will only publish funding information present in the Funding Statement section of the online submission form. Please remove any funding-related text from the manuscript.

5. Please include your full ethics statement in the ‘Methods’ section of your manuscript file. In your statement, please include the full name of the IRB or ethics committee who approved or waived your study, as well as whether or not you obtained informed written or verbal consent. If consent was waived for your study, please include this information in your statement as well.

6. If the reviewer comments include a recommendation to cite specific previously published works, please review and evaluate these publications to determine whether they are relevant and should be cited. There is no requirement to cite these works unless the editor has indicated otherwise.

Additional Editor Comments:

Please pay particular attention to comments regarding how the fNIRS data have been analysed and presented.

There are also several comments requesting that some statements in the discussion need to be modified to ensure they are fully in keeping with the what the data show.

The length of the introduction needs to be reduced considerably, and please ensure that the required information is all presented in the appropriate sections. The introduction also needs to provide a more robust theoretical justification for the study design and aims.

You may disregard comments from Reviewer 2 in this instance, as they have only provided 2 sentences that do not provide any value to the assessment of this manuscript. This reviewer will not be invited to review the next submission of this work.

[Note: HTML markup is below. Please do not edit.]

Reviewer's Responses to Questions

**Comments to the Author**

1. Is the manuscript technically sound, and do the data support the conclusions?

The manuscript must describe a technically sound piece of scientific research with data that supports the conclusions. Experiments must have been conducted rigorously, with appropriate controls, replication, and sample sizes. The conclusions must be drawn appropriately based on the data presented.

Reviewer #1: No

Reviewer #2: No

Reviewer #3: Yes

Reviewer #4: Partly

**********

2. Has the statistical analysis been performed appropriately and rigorously?

Reviewer #1: No

Reviewer #2: Yes

Reviewer #3: Yes

Reviewer #4: No

**********

3. Have the authors made all data underlying the findings in their manuscript fully available?

The PLOS Data policy requires authors to make all data underlying the findings described in their manuscript fully available without restriction, with rare exception (please refer to the Data Availability Statement in the manuscript PDF file). The data should be provided as part of the manuscript or its supporting information, or deposited to a public repository. For example, in addition to summary statistics, the data points behind means, medians and variance measures should be available. If there are restrictions on publicly sharing data—e.g. participant privacy or use of data from a third party—those must be specified.

Reviewer #1: Yes

Reviewer #2: Yes

Reviewer #3: Yes

Reviewer #4: Yes

**********

4. Is the manuscript presented in an intelligible fashion and written in standard English?

PLOS ONE does not copyedit accepted manuscripts, so the language in submitted articles must be clear, correct, and unambiguous. Any typographical or grammatical errors should be corrected at revision, so please note any specific errors here.

Reviewer #1: Yes

Reviewer #2: No

Reviewer #3: Yes

Reviewer #4: Yes

**********

5. Review Comments to the Author

Please use the space provided to explain your answers to the questions above. You may also include additional comments for the author, including concerns about dual publication, research ethics, or publication ethics. (Please upload your review as an attachment if it exceeds 20,000 characters)

Reviewer #1: Thank you for the invitation to review. I have the following comments:

1. Introduction:

a. In several places the text uses “effort cost” and “effort value” interchangeably, although these are two distinct psychological and neurobiological constructs. It would be helpful to emphasise more clearly that effort can be both costly and valuable at the same time, and that the perception of this relationship depends on context, the individual, and prior experience.

b. The impact of training on VoPE (Value of Physical Effort) may be modulated by factors such as previous sporting experience, temperamental traits, intrinsic motivation, or genetic predispositions (e.g. differences in the dopaminergic reward system). The text focuses mainly on group-level effects while overlooking potentially important individual differences.

c. The authors correctly note that most studies concern low to moderate intensities. However, there is no clear indication that high-intensity efforts (e.g. maximal intervals) may involve different neurophysiological mechanisms (e.g. stronger engagement of the opioid and endocannabinoid systems), which could alter the relationship between effort cost and value.

d. The discussion of near- and far-transfer is valuable, but it lacks an explicit acknowledgement that in the literature on physical effort the transfer of effects is far harder to demonstrate than in cognitive effort research. Learning mechanisms in the context of physical activity may be more task-specific, which should be highlighted as a potential limitation.

e. The argument relies on a few fMRI studies concerning the ACC and vmPFC. However, the methodological limitations are not addressed, such as the low statistical power of neuroimaging studies or the problem of reverse inference. Assigning overly direct functions to brain regions (e.g. ACC = “cost”, vmPFC = “net value”) risks oversimplification.

f. The text provides a good summary of the state of knowledge, but practical implications are missing. If regular training can alter the neural valuation of effort, it would be important to discuss how this knowledge could be applied in designing health interventions, e.g. in programmes for activating older adults, in rehabilitation, or in strategies to counteract sedentary lifestyles.

2. I recommend shortening the Methods section – it is overly lengthy and repetitive.

3. Procedure and Materials:

a. Selection of time window for fNIRS – using only the second minute of each interval (excluding onset and offset) is methodologically justified in terms of signal stabilisation and limiting artefacts, but may restrict the ability to capture effort dynamics. A comparative analysis with the full interval duration should be considered to ensure that important changes were not missed.

b. Definition of exercise intensity – basing intensity on metabolic thresholds (AT, RCP) is a strength as it allows individualisation of loads. Nevertheless, adopting fixed values (e.g. 85% AT, mean of AT and RCP, RCP + 5%) may produce differences in the subjective perception of effort between participants. Additional indices (e.g. % VO₂max, % HRmax) would be valuable.

c. The use of single-item questions and verbal scales is convenient but may reduce measurement sensitivity. Participants might struggle to differentiate states precisely in short time frames. Was test–retest reliability (ICC) checked? – 10.1016/j.jcm.2016.02.012

d. fNIRS measurement – the chosen optode configuration and sensitivity simulations support measurement reliability, but fNIRS in the vmPFC and preSMA is particularly susceptible to motion artefacts and circulatory confounds. The procedures for signal quality control (e.g. filtering, artefact correction, trial rejection) should be described in more detail.

e. The whole session lasts around two hours and involves both high-intensity exercise and additional measurement tasks. This may induce fatigue in participants, potentially affecting RPE, VoPE and physiological parameters. It should be stated how fatigue effects were controlled (e.g. recovery breaks, monitoring of resting HR between tasks).

4. L241 – “training group (n = 34) or a control group (n = 28).” – According to the conducted analyses, this sample size was sufficient to detect a large effect with 80% power. Please indicate this in the manuscript – 10.1016/j.apmr.2025.05.013

5. L361 – “used Bayesian repeated measures ANOVAs” – Please calculate effect sizes and provide confidence intervals. For group differences (Cohen’s d or Hedges’ g), small, medium, and large effects should correspond to 0.1, 0.4, and 0.8, respectively – 10.1016/j.apmr.2025.05.013

6. L366 – Bayes Factors and Cohen’s d represent different approaches: BF indicates the strength of evidence for a hypothesis, while d describes the magnitude of the effect. Literature (e.g. Lakens, 2013; van Doorn et al., 2021) often recommends reporting both, as they complement one another: Bayes Factor = evidential strength, Cohen’s d = effect size irrespective of evidence sufficiency.

7. Results – Please highlight which findings fall within medium and which within large effect sizes. In the conclusions, please base interpretations only on significant p values and large effect sizes.

8. L383 – I suggest closing the paragraph with the statistical outcomes, while the procedural details described by the authors should, in my opinion, be moved earlier.

9. Discussion and Conclusions:

a. The authors suggest that the lack of change in VoPE indicates its relative stability. However, it is possible that the effect was masked by the specifics of the intervention (high intensity, lack of systematic rewards). Therefore, the conclusion about “stability” should be stated more cautiously, with limitations of protocol sensitivity underlined.

b. The point that high intensity may be less beneficial than low or moderate intensity is valuable. However, no moderation analysis was performed – e.g. whether individual tolerance to effort (VO₂max, previous physical activity) influenced the results. Such an analysis could provide important insights into who benefits most from the intervention.

c. The authors refer to the theory of Learned Industriousness and attribute the lack of effect to the absence of rewards. This is a coherent interpretation, but the question remains whether a sense of competence (e.g. improvements in fitness tests) may not have been sufficiently emphasised. The absence of monitoring participants’ perception of progress is a significant limitation.

d. The discussion that VoPE ratings during effort may differ from retrospective evaluation is very valuable. However, the study lacks even a single retrospective measure (e.g. a post-training-cycle questionnaire). This omission prevents verification of one of the authors’ key hypotheses.

e. The observation that young people may be more susceptible to VoPE modification opens an interesting research avenue. Nevertheless, potential sex, cultural or personality differences (e.g. temperamental traits) that may modulate the learning of effort value should also be discussed.

f. The authors rightly note that fNIRS is limited to cortical surface areas and does not capture subcortical structures (e.g. nucleus accumbens). However, their conclusion that no effect was observed “neuronally” is too far-reaching. It should be made clear that the findings refer only to vmPFC and preSMA activity, not the entire reward or effort network.

Yours sincerely,

Reviewer #2: However, the manuscript is methodologically accepted, I recommend an extensive revision of the language used across the manuscript starting from the title itself as it is ambiguous and poorly written. In addition, I find it challenging to address any added value to the existing knowledge. the rationale of the research should clearly fill gaps in the literature.

Reviewer #3: The topic of the study is relevant, the results are interesting to the reader, and the manuscript is generally well written.

However, in my opinion, the text contains an excess of detail and unnecessary information, which forces the reader to go back and forth to fully understand the context. A manuscript exceeding 20 pages becomes difficult to follow. I strongly recommend that the authors summarize and streamline the content to improve readability and focus on the main findings.

For example:

The Introduction consists of ten paragraphs, which is considerably more than the average research paper. Several of these paragraphs serve as a literature review rather than a justification for the study.

The subsection titled Present Research should not appear within the Introduction; instead, it should be incorporated succinctly in the Methods section.

The description of the experimental protocol is excessively long and could be condensed.

The Data Analysis section extends to six paragraphs, followed by further detailed descriptions of data processing for each signal, which is again overly elaborate.

In Table 1, the first two variables present means and standard deviations as rounded numbers. Please ensure that all variables use the same number of decimal places.

Each results table is followed by three to four paragraphs of explanation, which is too extensive for a single table.

In summary, my comments aim to encourage the reduction of redundant and irrelevant information to help maintain the reader’s attention on the study’s key findings and contributions.

Reviewer #4: This paper looks at whether an 8-week high-intensity jump training program can change how people value physical effort and their willingness to try hard. This is an important question, and the study is ambitious, with strengths like a randomized controlled design, real-world relevance, multiple assessment methods (self-report, behavior, and fNIRS), high participation, and open data access. The paper is well-written, and the discussion offers useful insights about no significant findings.

However, several key issues need major changes before the paper can be published. These issues affect how we understand and trust the main conclusions.

Key Issues Needing Major Changes

Inconsistency in Study Design Reasoning

The paper mentions that high-intensity effort is often unpleasant. Despite this, the study uses a high-intensity program as the main intervention. This is inconsistent and might have reduced the chance of seeing positive changes. This choice must be clearly explained in the introduction, and the risks and challenges should be discussed openly.

fNIRS Data Processing Concerns

The authors used "less strict" methods to correct motion artifacts. This increases the risk of noise in the neural data, which might hide real effects. The authors should either:

Provide extra analyses with stricter filtering to show the results are strong, or

Be cautious in interpreting the neural findings, acknowledging that the results might be unreliable due to noise.

Baseline Group Differences in VoPE Not Addressed

The training group had higher VoPE than the control group at the start in the free ride task. This is a major issue that affects the fairness of the randomization. The authors need to:

Clearly show baseline differences,

Use statistical methods (e.g., ANCOVA) with baseline VoPE as a factor, and

Discuss how this affects the idea that VoPE is "stable."

Intervention Fails to Produce Overall Fitness Improvement

While jump height improved, VO₂peak and general fitness did not. This suggests the program might not have been strong enough to change higher-level motivation like VoPE. This should be seen not just as a lack of reinforcement but also as a possible failure of the program's strength.

In summary, while this study makes an important attempt to test the malleability of VoPE, the above concerns must be resolved before the conclusions can be considered robust. Addressing these issues will require additional analyses, reframing of theoretical justification, and methodological clarification. Therefore, I recommend Major Revision.

**********

6. PLOS authors have the option to publish the peer review history of their article (what does this mean?). If published, this will include your full peer review and any attached files.

If you choose “no”, your identity will remain anonymous but your review may still be made public.

**Do you want your identity to be public for this peer review?**  For information about this choice, including consent withdrawal, please see our Privacy Policy.

Reviewer #1: No

Reviewer #2: No

Reviewer #3: No

Reviewer #4: No

**********

[NOTE: If reviewer comments were submitted as an attachment file, they will be attached to this email and accessible via the submission site. Please log into your account, locate the manuscript record, and check for the action link "View Attachments". If this link does not appear, there are no attachment files.]

To ensure your figures meet our technical requirements, please review our figure guidelines: https://journals.plos.org/plosone/s/figures

You may also use PLOS’s free figure tool, NAAS, to help you prepare publication quality figures: https://journals.plos.org/plosone/s/figures#loc-tools-for-figure-preparation.

NAAS will assess whether your figures meet our technical requirements by comparing each figure against our figure specifications.

# Author comment: Author response to Decision Letter 1

**DOI:** 10.1371/journal.pone.0352897.r002

24 Jan 2026

Point-by-point reply

Manuscript number: #PONE-D-25-42417

Article title: An 8-week jump training did not boost effort value or the willingness to exert effort in a student sample

--------------------------------------------- comments Christopher Kirk ----------------------------------------

Dear XXX,

Thank you for submitting your manuscript to PLOS ONE. After careful consideration, we feel that it has merit but does not fully meet PLOS ONE’s publication criteria as it currently stands. Therefore, we invite you to submit a revised version of the manuscript that addresses the points raised during the review process.

Please submit your revised manuscript by Dec 25 2025 11:59PM. If you will need more time than this to complete your revisions, please reply to this message or contact the journal office at plosone@plos.org. When you're ready to submit your revision, log on to https://www.editorialmanager.com/pone/ and select the 'Submissions Needing Revision' folder to locate your manuscript file.

Please include the following items when submitting your revised manuscript:

• A rebuttal letter that responds to each point raised by the academic editor and reviewer(s). You should upload this letter as a separate file labeled 'Response to Reviewers'.

• A marked-up copy of your manuscript that highlights changes made to the original version. You should upload this as a separate file labeled 'Revised Manuscript with Track Changes'.

• An unmarked version of your revised paper without tracked changes. You should upload this as a separate file labeled 'Manuscript'.

If you would like to make changes to your financial disclosure, please include your updated statement in your cover letter. Guidelines for resubmitting your figure files are available below the reviewer comments at the end of this letter.

If applicable, we recommend that you deposit your laboratory protocols in protocols.io to enhance the reproducibility of your results. Protocols.io assigns your protocol its own identifier (DOI) so that it can be cited independently in the future. For instructions see: https://journals.plos.org/plosone/s/submission-guidelines#loc-laboratory-protocols. Additionally, PLOS ONE offers an option for publishing peer-reviewed Lab Protocol articles, which describe protocols hosted on protocols.io. Read more information on sharing protocols at https://plos.org/protocols?utm_medium=editorial-email&utm_source=authorletters&utm_campaign=protocols.

We look forward to receiving your revised manuscript.

Kind regards,

Christopher Kirk, PhD

Academic Editor

PLOS ONE

Journal Requirements:

When submitting your revision, we need you to address these additional requirements.

1.Please ensure that your manuscript meets PLOS ONE's style requirements, including those for file naming. The PLOS ONE style templates can be found at https://journals.plos.org/plosone/s/file?id=wjVg/PLOSOne_formatting_sample_main_body.pdf and

https://journals.plos.org/plosone/s/file?id=ba62/PLOSOne_formatting_sample_title_authors_affiliations.pdf

Thank you for pointing us to these templates. We have revised the manuscript accordingly.

2. Thank you for stating the following financial disclosure:

“The authors gratefully acknowledge funding from the Committee on Research (AFF) at the University of Konstanz for the research initiative "ProPELL: Promoting Physical Exercise in Lab and Life“”

Please state what role the funders took in the study. If the funders had no role, please state: "The funders had no role in study design, data collection and analysis, decision to publish, or preparation of the manuscript."

If this statement is not correct you must amend it as needed.

Please include this amended Role of Funder statement in your cover letter; we will change the online submission form on your behalf.

We appreciate your comment and have now included the Role of Funder statement in the cover letter.

3. We note that the grant information you provided in the ‘Funding Information’ and ‘Financial Disclosure’ sections do not match.

When you resubmit, please ensure that you provide the correct grant numbers for the awards you received for your study in the ‘Funding Information’ section.

Thank you for pointing this out. We have now revised this information.

4. Please note that funding information should not appear in any section or other areas of your manuscript. We will only publish funding information present in the Funding Statement section of the online submission form. Please remove any funding-related text from the manuscript.

We appreciate this guidance and have accordingly removed the funding statement from the manuscript.

5. Please include your full ethics statement in the ‘Methods’ section of your manuscript file. In your statement, please include the full name of the IRB or ethics committee who approved or waived your study, as well as whether or not you obtained informed written or verbal consent. If consent was waived for your study, please include this information in your statement as well.

Thank you for this comment. We have accordingly included the requested information in the Methods section (p. 13).

6. If the reviewer comments include a recommendation to cite specific previously published works, please review and evaluate these publications to determine whether they are relevant and should be cited. There is no requirement to cite these works unless the editor has indicated otherwise.

Additional Editor Comments:

Please pay particular attention to comments regarding how the fNIRS data have been analysed and presented.

There are also several comments requesting that some statements in the discussion need to be modified to ensure they are fully in keeping with the what the data show.

The length of the introduction needs to be reduced considerably, and please ensure that the required information is all presented in the appropriate sections. The introduction also needs to provide a more robust theoretical justification for the study design and aims.

You may disregard comments from Reviewer 2 in this instance, as they have only provided 2 sentences that do not provide any value to the assessment of this manuscript. This reviewer will not be invited to review the next submission of this work.

We would like to express our sincere gratitude to you and the reviewers for your constructive feedback on our manuscript. We have carefully considered your thoughtful comments and made revisions accordingly. In particular, we focused on reviewing the fNIRS-related sections, clarifying the discussion of the results, streamlining the introduction and shortening the manuscript to about 20 pages.

We believe all changes have greatly improved the quality of our manuscript, and we truly appreciate your time and effort in helping us enhance our work.

In the following, you will find our responses highlighted in blue, along with citations of the manuscript in bold and italics. Newly added passages related to the comment are indicated in red to ensure clarity.

-------------------------------------------------- comments Reviewer #1 ----------------------------------------

Thank you for the invitation to review. I have the following comments:

1. Introduction:

a. In several places the text uses “effort cost” and “effort value” interchangeably, although these are two distinct psychological and neurobiological constructs. It would be helpful to emphasise more clearly that effort can be both costly and valuable at the same time, and that the perception of this relationship depends on context, the individual, and prior experience.

We appreciate this helpful comment and fully agree that effort cost and value represent distinct constructs. We have carefully revised the manuscript to ensure that cost and value are no longer used interchangeably. In addition, we explicitly clarified that these constructs are conceptually distinct and may co-occur. We believe these revisions improve the conceptual clarity of the manuscript

“Conversely, accumulating evidence indicates that physical effort can simultaneously be perceived as valuable – at least to some degree, for some people, and in some situations (12,20).” (pp. 4-5)

“Consistent with this, theoretical work suggests mechanisms by which reduced effort aversiveness can increase individuals’ tendency to mobilize effort over time (see 12). One prominent example is Learned Industriousness, which proposes that repeatedly pairing effort with reward reduces effort’s aversiveness and increases willingness to exert effort (30). Notably, effort aversiveness can in principle be reduced via two mechanisms: by decreasing perceived effort costs and/or by increasing VoPE. However, whether Learned Industriousness also increases VoPE remains unclear, as both theoretical and empirical work have predominantly focused on reductions in effort costs (31–34) rather than potential increases in VoPE (20).” (p. 5)

“Thus, while previous research has begun to explore near-transfer effects in decision-making (31), it remains largely unclear whether regular physical effort alters how effort is valued (rather than its costs) during actual exertion, whether such changes generalize across tasks, and how they depend on exercise intensity (20).” (p. 7)

b. The impact of training on VoPE (Value of Physical Effort) may be modulated by factors such as previous sporting experience, temperamental traits, intrinsic motivation, or genetic predispositions (e.g. differences in the dopaminergic reward system). The text focuses mainly on group-level effects while overlooking potentially important individual differences.

We agree that the impact of training on the value of physical effort may vary across individuals as a function of prior sporting experience, motivational traits and other individual differences. As our study focuses on group-level effects, we now explicitly acknowledge this in the discussion and note that future work is needed to examine such moderating influences. (see our answer to your comment 9e)

“Another possible explanation lies in the developmental sensitivity of learning VoPE (20). Adolescents appear to be less sensitive to effort costs and exert more effort relative to task demands and rewards at stake than adults (78,79). This period of heightened responsiveness may facilitate the learning of the relationship between effort and reward, making it easier to internalize the contingency that greater effort leads to greater rewards. By contrast, by the time individuals reach early adulthood, as in the current sample, it may be more challenging to relearn or modify their VoPE. While this observation opens an interesting avenue for developmental research, it is also important to consider potential moderating factors such as previous learning experiences in the sport context, or genetic predispositions (e.g., differences in the dopaminergic reward system) that may influence how VoPE is learned. Future research should therefore test similar interventions with adolescent samples and systematically examine individual differences that may shape sensitivity to learning VoPE.” (pp. 37-38)

c. The authors correctly note that most studies concern low to moderate intensities. However, there is no clear indication that high-intensity efforts (e.g. maximal intervals) may involve different neurophysiological mechanisms (e.g. stronger engagement of the opioid and endocannabinoid systems), which could alter the relationship between effort cost and value.

This is an important comment, and we thank the reviewer for raising this point. In response, we have revised the Introduction to explicitly note that different exercise intensities may engage distinct neurophysiological mechanisms, particularly with respect to the endogenous opioid and endocannabinoid systems. These intensity-related differences may, in turn, influence the valuation of physical effort, thereby strengthening the rationale for considering high-intensity exercise (see also Reviewer #4, comment 1).

“In Bernacer et al. (31), initially inactive participants completed a three-month low-intensity exercise program, after which effort costs had less influence on effort-based decisions, accompanied by decreased effort-related activity in the anterior cingulate cortex (ACC) (31). These findings suggest that regular exercise may reduce effort costs and alter neural responses to effort (31). However, as effort intensity was low and higher intensities are often valued less (26) and perceived as more aversive (39–41), it remains unclear whether training at higher intensities, might yield stronger effects on effort valuation. Notably, high-intensity exercise may engages additional neurophysiological mechanisms, such as increased activation of the endogenous opioid and endocannabinoid systems, which have been linked to affective responses and reward processing during intense exertion (39,40). Thus, while previous research has begun to explore near-transfer effects in decision-making (31), it remains largely unclear whether regular physical effort alters how effort is valued (rather than its costs) during actual exertion, whether such changes generalize across tasks, and how they depend on exercise intensity (20).” (pp. 6-7)

d. The discussion of near- and far-transfer is valuable, but it lacks an explicit acknowledgement that in the literature on physical effort the transfer of effects is far harder to demonstrate than in cognitive effort research. Learning mechanisms in the context of physical activity may be more task-specific, which should be highlighted as a potential limitation.

Thank you for this important comment. We have revised the Discussion to explicitly acknowledge that evidence for transfer effects in the domain of physical effort is considerably more limited than in cognitive effort research. In particular, we now highlight that far-transfer effects are more difficult to demonstrate for physical effort and that learning mechanisms in the context of physical activity tend to be more task- and context-specific.

“Importantly, evidence for transfer effects in the domain of physical effort is considerably more limited and less consistent than in cognitive effort research, with learning effects often appearing to be task- and context-specific (75). Although previous studies have demonstrated both near- and far-transfer effects, like reduced perceived physical effort costs and increased industriousness, in both animals (76) and humans (31), the conditions under which such transfer effects occur remain poorly understood.“ (p. 34)

e. The argument relies on a few fMRI studies concerning the ACC and vmPFC. However, the methodological limitations are not addressed, such as the low statistical power of neuroimaging studies or the problem of reverse inference. Assigning overly direct functions to brain regions (e.g. ACC = “cost”, vmPFC = “net value”) risks oversimplification.

Thank you for highlighting this important conceptual and methodological concern. We have revised the Discussion and the interpretation of our results now explicitly takes these limitations into account. In line with your suggestions, we now clarify that the assignment of roles to specific brain regions represents a simplified framework, rather than definitive functional localization. We further emphasize that this simplification is useful at the current stage of research but should be interpreted with caution when discussing our findings.

Discussion: “Finally, the current study assessed neural activation during periods of (partially) intense physical exertion, which offers valuable ecological validity. However, physical exercise involves multiple concurrent cognitive processes (80,81), which may obscure neural mechanisms specific to VoPE. Moreover, interpreting activation in specific brain regions necessarily relies on simplified heuristic functional attributions, which entails the risk of oversimplification and reverse inference. While this approach was useful for addressing the present research question at the current stage of the literature, it may have constrained our ability to draw clearer inferences, as network neuroscience emphasizes that functions likely emerge from interactions rather than one-to-one map

Attachment Submitted filename: Response to Reviewers.docx

# Peer review: Decision Letter 1

**DOI:** 10.1371/journal.pone.0352897.r003

**Authors:** Christopher Kirk, Christopher Kirk

6 Mar 2026

PONE-D-25-42417R1

An 8-week jump training did not boost effort value or the willingness to exert effort in a student sample

PLOS One

Dear Dr. Stähler,

Thank you for submitting your manuscript to PLOS ONE. After careful consideration, we feel that it has merit but does not fully meet PLOS ONE’s publication criteria as it currently stands. Therefore, we invite you to submit a revised version of the manuscript that addresses the points raised during the review process.

Please submit your revised manuscript by Apr 20 2026 11:59PM. If you will need more time than this to complete your revisions, please reply to this message or contact the journal office at plosone@plos.org. When you're ready to submit your revision, log on to https://www.editorialmanager.com/pone/ and select the 'Submissions Needing Revision' folder to locate your manuscript file.

Please include the following items when submitting your revised manuscript:

- A letter that responds to each point raised by the academic editor and reviewer(s). You should upload this letter as a separate file labeled 'Response to Reviewers'.
- A marked-up copy of your manuscript that highlights changes made to the original version. You should upload this as a separate file labeled 'Revised Manuscript with Track Changes'.
- An unmarked version of your revised paper without tracked changes. You should upload this as a separate file labeled 'Manuscript'.

If you would like to make changes to your financial disclosure, please include your updated statement in your cover letter. Guidelines for resubmitting your figure files are available below the reviewer comments at the end of this letter.

If applicable, we recommend that you deposit your laboratory protocols in protocols.io to enhance the reproducibility of your results. Protocols.io assigns your protocol its own identifier (DOI) so that it can be cited independently in the future. For instructions see: https://journals.plos.org/plosone/s/submission-guidelines#loc-laboratory-protocols. Additionally, PLOS ONE offers an option for publishing peer-reviewed Lab Protocol articles, which describe protocols hosted on protocols.io. Read more information on sharing protocols at https://plos.org/protocols?utm_medium=editorial-email&utm_source=authorletters&utm_campaign=protocols.

We look forward to receiving your revised manuscript.

Kind regards,

Christopher Kirk, PhD

Academic Editor

PLOS One

Journal Requirements:

1. If the reviewer comments include a recommendation to cite specific previously published works, please review and evaluate these publications to determine whether they are relevant and should be cited. There is no requirement to cite these works unless the editor has indicated otherwise.

Additional Editor Comments:

Two reviewers are asking for relatively simple amendements to the structure of the manuscript, and specific details regarding the interpretation of the reported effect sizes. The third reviewer has highlighted some more detailed requirements about how of the variables and measurement methods have been discussed and interpreted.

[Note: HTML markup is below. Please do not edit.]

Reviewer's Responses to Questions

**Comments to the Author**

1. If the authors have adequately addressed your comments raised in a previous round of review and you feel that this manuscript is now acceptable for publication, you may indicate that here to bypass the “Comments to the Author” section, enter your conflict of interest statement in the “Confidential to Editor” section, and submit your "Accept" recommendation.

Reviewer #1: (No Response)

Reviewer #3: All comments have been addressed

Reviewer #4: (No Response)

**********

2. Is the manuscript technically sound, and do the data support the conclusions?

The manuscript must describe a technically sound piece of scientific research with data that supports the conclusions. Experiments must have been conducted rigorously, with appropriate controls, replication, and sample sizes. The conclusions must be drawn appropriately based on the data presented.

Reviewer #1: (No Response)

Reviewer #3: Yes

Reviewer #4: Partly

**********

3. Has the statistical analysis been performed appropriately and rigorously?

Reviewer #1: (No Response)

Reviewer #3: Yes

Reviewer #4: No

**********

4. Have the authors made all data underlying the findings in their manuscript fully available?

The PLOS Data policy requires authors to make all data underlying the findings described in their manuscript fully available without restriction, with rare exception (please refer to the Data Availability Statement in the manuscript PDF file). The data should be provided as part of the manuscript or its supporting information, or deposited to a public repository. For example, in addition to summary statistics, the data points behind means, medians and variance measures should be available. If there are restrictions on publicly sharing data—e.g. participant privacy or use of data from a third party—those must be specified.

Reviewer #1: (No Response)

Reviewer #3: Yes

Reviewer #4: Yes

**********

5. Is the manuscript presented in an intelligible fashion and written in standard English?

PLOS ONE does not copyedit accepted manuscripts, so the language in submitted articles must be clear, correct, and unambiguous. Any typographical or grammatical errors should be corrected at revision, so please note any specific errors here.

Reviewer #1: (No Response)

Reviewer #3: Yes

Reviewer #4: Yes

**********

6. Review Comments to the Author

Please use the space provided to explain your answers to the questions above. You may also include additional comments for the author, including concerns about dual publication, research ethics, or publication ethics. (Please upload your review as an attachment if it exceeds 20,000 characters)

Reviewer #1: In the section on statistical analyses, the authors state that ‘Effect sizes were calculated to quantify the strength of any observed effects using Cohen’s d along with corresponding 95% confidence intervals’. However, they do not specify which effect size magnitudes these calculations refer to, nor which interpretative thresholds were applied. This constitutes a methodological shortcoming, as there exists a broad spectrum of effect size magnitudes, and without explicit criteria it is unclear how the authors interpret their results. For this reason, I suggested providing concrete thresholds. Specifically, for Cohen’s d, the following cut-offs should be used: 0.1, 0.4, and 0.8, respectively, with the corresponding citation (DOI: 10.1016/j.apmr.2025.05.013). Additionally, for ANOVA, the following thresholds should be reported: Small: W ≥ 0.1, Medium: W ≥ 0.3, Large: W ≥ 0.5, with appropriate citation (DOI: 10.11613/BM.2026.010101). According to these guidelines, the analysis should have been conducted accordingly; however, this information is still absent from the manuscript. Yours sincerely

Reviewer #3: I appreciate the effort the authors have made in revising the manuscript. The text is now considerably clearer and more pleasant to read.

In this review, I will focus primarily on the revisions made in response to my initial comments, as I recognize that several additional modifications were implemented to address remarks from other reviewers.

Introduction: Although the section has been substantially reduced, the inclusion of subtopics within the introduction is not standard practice. I recommend restructuring this section to present a more direct and straightforward justification of the study.

Objectives: The objectives are currently presented as a justification for the study rather than as clear statements. I suggest rewriting them in an affirmative and explicit manner.

Conclusions: The conclusions refer only to VoPE. It would be appropriate to also incorporate the other variables examined in the study to provide a more comprehensive summary of the findings.

References: Approximately 87 references appear excessive for a single article. I recommend reducing the number to around 60, ensuring that only the most relevant and essential sources are retained.

Reviewer #4: Dear Authors,

Thanks to you, your work has been revised. The manuscript is improved; however, there are still several critical points that should be corrected to be published.

1. fNIRS Data Robustness (High Priority) you failed to perform the sensitivity analysis with more stringent motor-correction parameters. Signal quality aspects such as percent rejected channels, SNR or channel acceptance rates were not reported. Findings of neural nulls are not explained well in the light of potential motion artifact.

Required: Conduct more severe settings sensitivity analysis, report signal quality metrics, and talk more about motion related limitations.

2. Multiple Comparisons (High Priority) You compared a large number of behavioral and neural outcomes and at various levels and times. You have not mentioned whether you have made adjustments to several comparisons. The risk management of Type I errors is not clear.

Note: indicate whether you used multiplicity corrections. Otherwise, justify and distinguish between confirmatory and exploratory analysis.

3. Baseline Transparency (High Priority) Your ANCOVA, but no CONSORT-type baseline equivalence table. Group comparability on demographics and baseline variables were not clearly reported.

Necessary: Provide a detailed and presented Table 1 with a demographic and baseline comparison, statistical tests, and effect sizes.

4. VoPE Stability (Medium Priority) Conclusions may be excessive. Boundary conditions are not clearly spelt out.

Limitations: Conclusions may be made only pertaining to: High-intensity, non-reinforced training, Young, low-active adults, Concurrent measurement paradigm.

5. Limitations of Moderation Analysis (Medium Priority) You failed to perform subgroup analyses or moderation. It is a limitation that is brought up but not emphasized.

Additional: Add a limitation paragraph explaining clearly why moderation could not be done, why (e.g., VO 2max, prior activity, effort tolerance) should be measured in the future studies.

**********

7. PLOS authors have the option to publish the peer review history of their article (what does this mean?). If published, this will include your full peer review and any attached files.

If you choose “no”, your identity will remain anonymous but your review may still be made public.

**Do you want your identity to be public for this peer review?**  For information about this choice, including consent withdrawal, please see our Privacy Policy.

Reviewer #1: No

Reviewer #3: No

Reviewer #4: No

**********

[NOTE: If reviewer comments were submitted as an attachment file, they will be attached to this email and accessible via the submission site. Please log into your account, locate the manuscript record, and check for the action link "View Attachments". If this link does not appear, there are no attachment files.]

To ensure your figures meet our technical requirements, please review our figure guidelines: https://journals.plos.org/plosone/s/figures

You may also use PLOS’s free figure tool, NAAS, to help you prepare publication quality figures: https://journals.plos.org/plosone/s/figures#loc-tools-for-figure-preparation.

NAAS will assess whether your figures meet our technical requirements by comparing each figure against our figure specifications.

# Author comment: Author response to Decision Letter 2

**DOI:** 10.1371/journal.pone.0352897.r004

12 Apr 2026

Point-by-point reply

Manuscript number: #PONE-D-25-42417

Article title: An 8-week jump training did not boost effort value or the willingness to exert effort in a student sample

--------------------------------------------- comments Christopher Kirk ----------------------------------------

Dear XXX,

Thank you for submitting your manuscript to PLOS ONE. After careful consideration, we feel that it has merit but does not fully meet PLOS ONE’s publication criteria as it currently stands. Therefore, we invite you to submit a revised version of the manuscript that addresses the points raised during the review process.

Please submit your revised manuscript by Apr 20 2026 11:59PM. If you will need more time than this to complete your revisions, please reply to this message or contact the journal office at plosone@plos.org. When you're ready to submit your revision, log on to https://www.editorialmanager.com/pone/ and select the 'Submissions Needing Revision' folder to locate your manuscript file.

Please include the following items when submitting your revised manuscript:

• A letter that responds to each point raised by the academic editor and reviewer(s). You should upload this letter as a separate file labeled 'Response to Reviewers'.

• A marked-up copy of your manuscript that highlights changes made to the original version. You should upload this as a separate file labeled 'Revised Manuscript with Track Changes'.

• An unmarked version of your revised paper without tracked changes. You should upload this as a separate file labeled 'Manuscript'.

If you would like to make changes to your financial disclosure, please include your updated statement in your cover letter. Guidelines for resubmitting your figure files are available below the reviewer comments at the end of this letter.

If applicable, we recommend that you deposit your laboratory protocols in protocols.io to enhance the reproducibility of your results. Protocols.io assigns your protocol its own identifier (DOI) so that it can be cited independently in the future. For instructions see: https://journals.plos.org/plosone/s/submission-guidelines#loc-laboratory-protocols. Additionally, PLOS ONE offers an option for publishing peer-reviewed Lab Protocol articles, which describe protocols hosted on protocols.io. Read more information on sharing protocols at https://plos.org/protocols?utm_medium=editorial-email&utm_source=authorletters&utm_campaign=protocols.

We look forward to receiving your revised manuscript.

Kind regards,

Christopher Kirk, PhD

Academic Editor

PLOS One

Journal Requirements:

1. If the reviewer comments include a recommendation to cite specific previously published works, please review and evaluate these publications to determine whether they are relevant and should be cited. There is no requirement to cite these works unless the editor has indicated otherwise.

Additional Editor Comments:

Two reviewers are asking for relatively simple amendments to the structure of the manuscript, and specific details regarding the interpretation of the reported effect sizes. The third reviewer has highlighted some more detailed requirements about how of the variables and measurement methods have been discussed and interpreted.

We sincerely thank you for your continued constructive and helpful feedback. We have revised the manuscript accordingly and improved the overall structure of the Introduction to enhance clarity and readability. In addition, we now report and more carefully interpret effect sizes.

Furthermore, we reprocessed the fNIRS data using an alternative preprocessing pipeline to assess the robustness of our findings. The results remained consistent, supporting the stability of our results. We have also strengthened the discussion by placing the findings more clearly within the broader context of the study.

Lastly, we reviewed the abstract to make it clearer, remove redundancies, and improve readability.

“Physical inactivity remains highly prevalent, partly driven by the aversive nature of effort. However, effort can also be experienced as rewarding, which is associated with greater overall physical activity. Accordingly, the present study investigates whether regular physical exercise alters the self-reported value of physical effort, neural activation during exercise, and the willingness to exert effort. Sixty-two young adults were assigned to either an eight-week high-intensity jump training or a control group. Participants completed a two-task cycling ergometer exercise before and after the intervention. The first task assessed the value of effort at three pre-determined intensity levels, whereas the second task allowed participants to self-select the intensity to measure their willingness to exert effort. Participants’ perceived exertion and state value of physical effort were assessed, along with neural activation in the ventromedial prefrontal cortex and pre-supplementary motor area using functional near-infrared spectroscopy. Bayesian analyses provided evidence against main effects of condition, time, as well as their interaction, for self-reported value of physical effort, willingness to exert effort, and neural activity. This suggests that the value of effort may be relatively stable, as an eight-week training intervention did not alter the value of physical effort or the willingness to exert effort.” (p. 3)

We believe that these revisions have again improved the manuscript and appreciate the opportunity to refine our work.

-------------------------------------------------- comments Reviewer #1 ----------------------------------------

In the section on statistical analyses, the authors state that ‘Effect sizes were calculated to quantify the strength of any observed effects using Cohen’s d along with corresponding 95% confidence intervals’. However, they do not specify which effect size magnitudes these calculations refer to, nor which interpretative thresholds were applied. This constitutes a methodological shortcoming, as there exists a broad spectrum of effect size magnitudes, and without explicit criteria it is unclear how the authors interpret their results. For this reason, I suggested providing concrete thresholds. Specifically, for Cohen’s d, the following cut-offs should be used: 0.1, 0.4, and 0.8, respectively, with the corresponding citation (DOI: 10.1016/j.apmr.2025.05.013). Additionally, for ANOVA, the following thresholds should be reported: Small: W ≥ 0.1, Medium: W ≥ 0.3, Large: W ≥ 0.5, with appropriate citation (DOI: 10.11613/BM.2026.010101). According to these guidelines, the analysis should have been conducted accordingly; however, this information is still absent from the manuscript. Yours sincerely

We thank the reviewer for this suggestion. However, we followed the conventional thresholds for Cohen’s d (0.2 = small, 0.5 = medium, 0.8 = large) as originally proposed by Cohen (1988), which remain the most widely used and broadly accepted benchmark across disciplines.

For ANOVA effect sizes, we used ω² instead of Kendall’s W, which is more appropriate for variance-based effect size estimation in mixed ANOVA designs.

“In addition to Bayesian analyses, effect sizes were calculated to quantify the magnitude of observed effects. For ANOVA effects, ω² was reported (0.01 = small, 0.06 = medium, 0.14 = large) (50). For pairwise comparisons, Cohen’s d was reported (0.2 = small, 0.5 = medium, 0.8 = large), along with corresponding 95% confidence intervals (50).” (p. 21)

Results – see for example:

“The best model including the Time x Condition interaction, also included main effects of Time, Condition, and Interval, BF10 = 4.98, P(M/data) = .04, ω² = 0.017 (for complete results tables, see OSF), providing substantial evidence for the alternative hypothesis (51).” (p. 26)

------------------------------------------------ comments Reviewer #3 ------------------------------------------

I appreciate the effort the authors have made in revising the manuscript. The text is now considerably clearer and more pleasant to read.

In this review, I will focus primarily on the revisions made in response to my initial comments, as I recognize that several additional modifications were implemented to address remarks from other reviewers.

Thank you very much for this helpful feedback. We have addressed the comments accordingly and believe that these revisions have further improved our manuscript.

1. Introduction: Although the section has been substantially reduced, the inclusion of subtopics within the introduction is not standard practice. I recommend restructuring this section to present a more direct and straightforward justification of the study.

Thank you for this remark. We have revised the introduction so that separate subtopics have been integrated into the main text. The section has also been streamlined and more clearly focused on the present study, providing a more direct justification of our research. (See pp. 4-11)

2. Objectives: The objectives are currently presented as a justification for the study rather than as clear statements. I suggest rewriting them in an affirmative and explicit manner.

Thank you for this helpful suggestion. The objectives are now presented more explicitly and in an affirmative manner. Additionally, the study aims are now stated more clearly to more directly outline the purpose of the study.

“The aim of the present study therefore was to examine whether regular physical exercise alters the value of physical effort during actual physical exertion. Specifically, we investigated whether changes in subjective, behavioral and neural indicators of effort valuation generalized beyond the training task.” (p. 9)

3. Conclusions: The conclusions refer only to VoPE. It would be appropriate to also incorporate the other variables examined in the study to provide a more comprehensive summary of the findings.

We appreciate this helpful suggestion and revised the conclusion to reflect the broader set of variables. In particular, we now explicitly refer to the behavioral and neural measures alongside VoPE to provide a more comprehensive summary of the study’s findings. (See also our response to Reviewer 4, comment #4)

“In summary, this study provides initial insights into whether regular physical exercise can influence the valuation of physical effort during exercise across behavioral, subjective, and neural levels. Contrary to our expectations, eight weeks of high-intensity jump training did not alter participants’ willingness to exert effort, their self-reported value of physical effort (VoPE), or neural responses in the vmPFC associated with effort valuation. Together, these findings suggest a relative stability of concurrent effort valuation in the context of the present training paradigm. Future research should therefore examine whether different training characteristics (e.g., training intensity), timing of VoPE assessment, the role of intrinsic and extrinsic rewards, or other populations (e.g., active individuals, adolescents), influence the modifiability of effort valuation.” (p. 41)

References: Approximately 87 references appear excessive for a single article. I recommend reducing the number to around 60, ensuring that only the most relevant and essential sources are retained.

Thank you for this suggestion. We have carefully reviewed the references and reduced the number of citations to 61 by removing less essential and redundant references while retaining the most relevant sources necessary to support the argumentation and theoretical background of the manuscript.

------------------------------------------------ comments Reviewer #4 ------------------------------------------

Thanks to you, your work has been revised. The manuscript is improved; however, there are still several critical points that should be corrected to be published.

1. fNIRS Data Robustness (High Priority) you failed to perform the sensitivity analysis with more stringent motor-correction parameters. Signal quality aspects such as percent rejected channels, SNR or channel acceptance rates were not reported. Findings of neural nulls are not explained well in the light of potential motion artifact.

Required: Conduct more severe settings sensitivity analysis, report signal quality metrics, and talk more about motion related limitations.

We thank the reviewer for this important and constructive comment. To assess robustness of the present results against different preprocessing choices, we preprocessed the data with another processing stream that has been used in prior research that employed fNIRS in the context of a physical effort task (see, doi: 10.1038/s41598-018-34009-2). This did not change our results. If the editor wishes, we would be happy to provide these additional analyses as supplementary material.

enPruneChannels function: remove channels when the signal was too weak or too strong

Intensity_to_OD: optical intensity converted to optical density

Wavelet_Motion_Correction: remove motion artifacts (IQR of 1).

low pass filter (0.5 Hz)

converted to oxy- and deoxyhemoglobin with the modified Beer-Lambert law

Furthermore, we have now included explicit reporting of signal quality metrics in the Method section:

“Overall, 6.3% of channels were rejected due to insufficient signal quality. Of these rejected channels, 29% were excluded automatically by the preprocessing stream, while the remaining 71% were identified and removed through manual inspection.” (p. 19)

Finally, we have expanded the Discussion to more explicitly address the potential impact of motion-related artifacts:

“Finally, the current study assessed neural activation during periods of (partially) intense physical exertion, which offers valuable ecological validity. However, although fNIRS is relatively robust to motion artifacts (56), it is important to note that motion-related noise cannot be fully excluded, particularly in the context of physical demanding tasks such as a cycling task. Despite careful preprocessing and quality control procedures, residual motion artifacts may have influenced the signal and could have contributed to the observed null findings. Importantly, however, no corresponding effects were observed at the behavioral or subjective level, suggesting that the absence of neural differences is consistent across multiple levels of analysis and not solely attributable to measurement noise.” (p. 39)

2. Multiple Comparisons (High Priority) You compared a large number of behavioral and neural outcomes and at various levels and times. You have not mentioned whether you have made adjustments to several comparisons. The risk management of Type I errors is not clear.

Note: indicate whether you used multiplicity corrections. Otherwise, justify and distinguish between confirmatory and exploratory analysis.

Thank you for this important comment. We agree that the large number of comparisons requires careful consideration and transparent reporting.

All primary analyses were conducted within a Bayesian framework, in which multiplicity corrections are not required (e.g., 10.1080/19345747.2011.618213), as Bayes factors quantify the relative evidence for each model given the observed data, rather than relying on a repeated-sampling logic that necessitates control of Type I error rates (10.3758/s13423-017-1343-3). Nevertheless, we acknowledge that conducting multiple analyses increases the risk of overinterpretation. To further strengthen the interpretability of our findings and guard against over-interpretation, we now explicitly label analyses as confirmatory or exploratory in the Methods section.

“For state VoPE and cerebral oxygenation (oxygenated (HbO) and de-oxygenated (HbR), hemoglobin concentration) of the interval task, mean values of the second minute of the ride were analyzed using the same 2-between (condition: TG, CG) x 3-within (intensity levels: low, moderate, vigorous) x 2-within (time: pre- and post-training) Bayesian repeated measures ANOVA design in JASP. For the free ride task, 2-between (condition: TG, CG) x 2-within (time: pre- and post-training) Bayesian repeated measures ANOVAs were conducted for heart rate (10-minute mean), RPE, rendered power (10-minute mean, normalized to the RCP), and state VoPE. All

Attachment Submitted filename: Response_to_Reviewers_auresp_2.docx

# Editor report: Decision Letter 2

**DOI:** 10.1371/journal.pone.0352897.r005

**Authors:** Christopher Kirk, Christopher Kirk, Christopher Kirk

21 Apr 2026

PONE-D-25-42417R2

An 8-week jump training did not boost effort value or the willingness to exert effort in a student sample

PLOS One

Dear Dr. Stähler,

Thank you for submitting your manuscript to PLOS ONE. After careful consideration, we feel that it has merit but does not fully meet PLOS ONE’s publication criteria as it currently stands. Therefore, we invite you to submit a revised version of the manuscript that addresses the points raised during the review process.

Two reviewers have provided realtively minor amendments to make for clarity and confirmation. The third reviewer has some more substantial edits required, please consider each accordingly.

Please submit your revised manuscript by Jun 05 2026 11:59PM. If you will need more time than this to complete your revisions, please reply to this message or contact the journal office at plosone@plos.org. When you're ready to submit your revision, log on to https://www.editorialmanager.com/pone/ and select the 'Submissions Needing Revision' folder to locate your manuscript file.

Please include the following items when submitting your revised manuscript:

- A letter that responds to each point raised by the academic editor and reviewer(s). You should upload this letter as a separate file labeled 'Response to Reviewers'.
- A marked-up copy of your manuscript that highlights changes made to the original version. You should upload this as a separate file labeled 'Revised Manuscript with Track Changes'.
- An unmarked version of your revised paper without tracked changes. You should upload this as a separate file labeled 'Manuscript'.

If you would like to make changes to your financial disclosure, please include your updated statement in your cover letter. Guidelines for resubmitting your figure files are available below the reviewer comments at the end of this letter.

If applicable, we recommend that you deposit your laboratory protocols in protocols.io to enhance the reproducibility of your results. Protocols.io assigns your protocol its own identifier (DOI) so that it can be cited independently in the future. For instructions see: https://journals.plos.org/plosone/s/submission-guidelines#loc-laboratory-protocols. Additionally, PLOS ONE offers an option for publishing peer-reviewed Lab Protocol articles, which describe protocols hosted on protocols.io. Read more information on sharing protocols at https://plos.org/protocols?utm_medium=editorial-email&utm_source=authorletters&utm_campaign=protocols.

As the corresponding author, your ORCID iD is verified in the submission system and will appear in the published article. PLOS supports the use of ORCID, and we encourage all coauthors to register for an ORCID iD and use it as well. Please encourage your coauthors to verify their ORCID iD within the submission system before final acceptance, as unverified ORCID iDs will not appear in the published article. *Only*  the individual author can complete the verification step; PLOS staff *cannot*  verify ORCID iDs on behalf of authors.

We look forward to receiving your revised manuscript.

Kind regards,

Christopher Kirk, PhD, FCASES

Academic Editor

PLOS One

Journal Requirements:

If the reviewer comments include a recommendation to cite specific previously published works, please review and evaluate these publications to determine whether they are relevant and should be cited. There is no requirement to cite these works unless the editor has indicated otherwise.

[Note: HTML markup is below. Please do not edit.]

[NOTE: If reviewer comments were submitted as an attachment file, they will be attached to this email and accessible via the submission site. Please log into your account, locate the manuscript record, and check for the action link "View Attachments". If this link does not appear, there are no attachment files.]

To ensure your figures meet our technical requirements, please review our figure guidelines: https://journals.plos.org/plosone/s/figures

You may also use PLOS’s free figure tool, NAAS, to help you prepare publication quality figures: https://journals.plos.org/plosone/s/figures#loc-tools-for-figure-preparation.

NAAS will assess whether your figures meet our technical requirements by comparing each figure against our figure specifications.

# Author comment: Author response to Decision Letter 3

**DOI:** 10.1371/journal.pone.0352897.r006

12 May 2026

Point-by-point reply

Manuscript number: #PONE-D-25-42417

Article title: An 8-week jump training did not boost effort value or the willingness to exert effort in a student sample

--------------------------------------------- comments Christopher Kirk ----------------------------------------

Dear XXX,

Thank you for submitting your manuscript to PLOS ONE. After careful consideration, we feel that it has merit but does not fully meet PLOS ONE’s publication criteria as it currently stands. Therefore, we invite you to submit a revised version of the manuscript that addresses the points raised during the review process.

Please submit your revised manuscript by Apr 20 2026 11:59PM. If you will need more time than this to complete your revisions, please reply to this message or contact the journal office at plosone@plos.org. When you're ready to submit your revision, log on to https://www.editorialmanager.com/pone/ and select the 'Submissions Needing Revision' folder to locate your manuscript file.

Please include the following items when submitting your revised manuscript:

• A letter that responds to each point raised by the academic editor and reviewer(s). You should upload this letter as a separate file labeled 'Response to Reviewers'.

• A marked-up copy of your manuscript that highlights changes made to the original version. You should upload this as a separate file labeled 'Revised Manuscript with Track Changes'.

• An unmarked version of your revised paper without tracked changes. You should upload this as a separate file labeled 'Manuscript'.

If you would like to make changes to your financial disclosure, please include your updated statement in your cover letter. Guidelines for resubmitting your figure files are available below the reviewer comments at the end of this letter.

If applicable, we recommend that you deposit your laboratory protocols in protocols.io to enhance the reproducibility of your results. Protocols.io assigns your protocol its own identifier (DOI) so that it can be cited independently in the future. For instructions see: https://journals.plos.org/plosone/s/submission-guidelines#loc-laboratory-protocols. Additionally, PLOS ONE offers an option for publishing peer-reviewed Lab Protocol articles, which describe protocols hosted on protocols.io. Read more information on sharing protocols at https://plos.org/protocols?utm_medium=editorial-email&utm_source=authorletters&utm_campaign=protocols.

We look forward to receiving your revised manuscript.

Kind regards,

Christopher Kirk, PhD

Academic Editor

PLOS One

Journal Requirements:

1. If the reviewer comments include a recommendation to cite specific previously published works, please review and evaluate these publications to determine whether they are relevant and should be cited. There is no requirement to cite these works unless the editor has indicated otherwise.

Additional Editor Comments:

Two reviewers are asking for relatively simple amendments to the structure of the manuscript, and specific details regarding the interpretation of the reported effect sizes. The third reviewer has highlighted some more detailed requirements about how of the variables and measurement methods have been discussed and interpreted.

We sincerely thank you for your continued constructive and helpful feedback. We have revised the manuscript accordingly and improved the overall structure of the Introduction to enhance clarity and readability. In addition, we now report and more carefully interpret effect sizes.

Furthermore, we reprocessed the fNIRS data using an alternative preprocessing pipeline to assess the robustness of our findings. The results remained consistent, supporting the stability of our results. We have also strengthened the discussion by placing the findings more clearly within the broader context of the study.

Lastly, we reviewed the abstract to make it clearer, remove redundancies, and improve readability.

“Physical inactivity remains highly prevalent, partly driven by the aversive nature of effort. However, effort can also be experienced as rewarding, which is associated with greater overall physical activity. Accordingly, the present study investigates whether regular physical exercise alters the self-reported value of physical effort, neural activation during exercise, and the willingness to exert effort. Sixty-two young adults were assigned to either an eight-week high-intensity jump training or a control group. Participants completed a two-task cycling ergometer exercise before and after the intervention. The first task assessed the value of effort at three pre-determined intensity levels, whereas the second task allowed participants to self-select the intensity to measure their willingness to exert effort. Participants’ perceived exertion and state value of physical effort were assessed, along with neural activation in the ventromedial prefrontal cortex and pre-supplementary motor area using functional near-infrared spectroscopy. Bayesian analyses provided evidence against main effects of condition, time, as well as their interaction, for self-reported value of physical effort, willingness to exert effort, and neural activity. This suggests that the value of effort may be relatively stable, as an eight-week training intervention did not alter the value of physical effort or the willingness to exert effort.” (p. 3)

We believe that these revisions have again improved the manuscript and appreciate the opportunity to refine our work.

-------------------------------------------------- comments Reviewer #1 ----------------------------------------

In the section on statistical analyses, the authors state that ‘Effect sizes were calculated to quantify the strength of any observed effects using Cohen’s d along with corresponding 95% confidence intervals’. However, they do not specify which effect size magnitudes these calculations refer to, nor which interpretative thresholds were applied. This constitutes a methodological shortcoming, as there exists a broad spectrum of effect size magnitudes, and without explicit criteria it is unclear how the authors interpret their results. For this reason, I suggested providing concrete thresholds. Specifically, for Cohen’s d, the following cut-offs should be used: 0.1, 0.4, and 0.8, respectively, with the corresponding citation (DOI: 10.1016/j.apmr.2025.05.013). Additionally, for ANOVA, the following thresholds should be reported: Small: W ≥ 0.1, Medium: W ≥ 0.3, Large: W ≥ 0.5, with appropriate citation (DOI: 10.11613/BM.2026.010101). According to these guidelines, the analysis should have been conducted accordingly; however, this information is still absent from the manuscript. Yours sincerely

We thank the reviewer for this suggestion. However, we followed the conventional thresholds for Cohen’s d (0.2 = small, 0.5 = medium, 0.8 = large) as originally proposed by Cohen (1988), which remain the most widely used and broadly accepted benchmark across disciplines.

For ANOVA effect sizes, we used ω² instead of Kendall’s W, which is more appropriate for variance-based effect size estimation in mixed ANOVA designs.

“In addition to Bayesian analyses, effect sizes were calculated to quantify the magnitude of observed effects. For ANOVA effects, ω² was reported (0.01 = small, 0.06 = medium, 0.14 = large) (50). For pairwise comparisons, Cohen’s d was reported (0.2 = small, 0.5 = medium, 0.8 = large), along with corresponding 95% confidence intervals (50).” (p. 21)

Results – see for example:

“The best model including the Time x Condition interaction, also included main effects of Time, Condition, and Interval, BF10 = 4.98, P(M/data) = .04, ω² = 0.017 (for complete results tables, see OSF), providing substantial evidence for the alternative hypothesis (51).” (p. 26)

------------------------------------------------ comments Reviewer #3 ------------------------------------------

I appreciate the effort the authors have made in revising the manuscript. The text is now considerably clearer and more pleasant to read.

In this review, I will focus primarily on the revisions made in response to my initial comments, as I recognize that several additional modifications were implemented to address remarks from other reviewers.

Thank you very much for this helpful feedback. We have addressed the comments accordingly and believe that these revisions have further improved our manuscript.

1. Introduction: Although the section has been substantially reduced, the inclusion of subtopics within the introduction is not standard practice. I recommend restructuring this section to present a more direct and straightforward justification of the study.

Thank you for this remark. We have revised the introduction so that separate subtopics have been integrated into the main text. The section has also been streamlined and more clearly focused on the present study, providing a more direct justification of our research. (See pp. 4-11)

2. Objectives: The objectives are currently presented as a justification for the study rather than as clear statements. I suggest rewriting them in an affirmative and explicit manner.

Thank you for this helpful suggestion. The objectives are now presented more explicitly and in an affirmative manner. Additionally, the study aims are now stated more clearly to more directly outline the purpose of the study.

“The aim of the present study therefore was to examine whether regular physical exercise alters the value of physical effort during actual physical exertion. Specifically, we investigated whether changes in subjective, behavioral and neural indicators of effort valuation generalized beyond the training task.” (p. 9)

3. Conclusions: The conclusions refer only to VoPE. It would be appropriate to also incorporate the other variables examined in the study to provide a more comprehensive summary of the findings.

We appreciate this helpful suggestion and revised the conclusion to reflect the broader set of variables. In particular, we now explicitly refer to the behavioral and neural measures alongside VoPE to provide a more comprehensive summary of the study’s findings. (See also our response to Reviewer 4, comment #4)

“In summary, this study provides initial insights into whether regular physical exercise can influence the valuation of physical effort during exercise across behavioral, subjective, and neural levels. Contrary to our expectations, eight weeks of high-intensity jump training did not alter participants’ willingness to exert effort, their self-reported value of physical effort (VoPE), or neural responses in the vmPFC associated with effort valuation. Together, these findings suggest a relative stability of concurrent effort valuation in the context of the present training paradigm. Future research should therefore examine whether different training characteristics (e.g., training intensity), timing of VoPE assessment, the role of intrinsic and extrinsic rewards, or other populations (e.g., active individuals, adolescents), influence the modifiability of effort valuation.” (p. 41)

References: Approximately 87 references appear excessive for a single article. I recommend reducing the number to around 60, ensuring that only the most relevant and essential sources are retained.

Thank you for this suggestion. We have carefully reviewed the references and reduced the number of citations to 61 by removing less essential and redundant references while retaining the most relevant sources necessary to support the argumentation and theoretical background of the manuscript.

------------------------------------------------ comments Reviewer #4 ------------------------------------------

Thanks to you, your work has been revised. The manuscript is improved; however, there are still several critical points that should be corrected to be published.

1. fNIRS Data Robustness (High Priority) you failed to perform the sensitivity analysis with more stringent motor-correction parameters. Signal quality aspects such as percent rejected channels, SNR or channel acceptance rates were not reported. Findings of neural nulls are not explained well in the light of potential motion artifact.

Required: Conduct more severe settings sensitivity analysis, report signal quality metrics, and talk more about motion related limitations.

We thank the reviewer for this important and constructive comment. To assess robustness of the present results against different preprocessing choices, we preprocessed the data with another processing stream that has been used in prior research that employed fNIRS in the context of a physical effort task (see, doi: 10.1038/s41598-018-34009-2). This did not change our results. If the editor wishes, we would be happy to provide these additional analyses as supplementary material.

enPruneChannels function: remove channels when the signal was too weak or too strong

Intensity_to_OD: optical intensity converted to optical density

Wavelet_Motion_Correction: remove motion artifacts (IQR of 1).

low pass filter (0.5 Hz)

converted to oxy- and deoxyhemoglobin with the modified Beer-Lambert law

Furthermore, we have now included explicit reporting of signal quality metrics in the Method section:

“Overall, 6.3% of channels were rejected due to insufficient signal quality. Of these rejected channels, 29% were excluded automatically by the preprocessing stream, while the remaining 71% were identified and removed through manual inspection.” (p. 19)

Finally, we have expanded the Discussion to more explicitly address the potential impact of motion-related artifacts:

“Finally, the current study assessed neural activation during periods of (partially) intense physical exertion, which offers valuable ecological validity. However, although fNIRS is relatively robust to motion artifacts (56), it is important to note that motion-related noise cannot be fully excluded, particularly in the context of physical demanding tasks such as a cycling task. Despite careful preprocessing and quality control procedures, residual motion artifacts may have influenced the signal and could have contributed to the observed null findings. Importantly, however, no corresponding effects were observed at the behavioral or subjective level, suggesting that the absence of neural differences is consistent across multiple levels of analysis and not solely attributable to measurement noise.” (p. 39)

2. Multiple Comparisons (High Priority) You compared a large number of behavioral and neural outcomes and at various levels and times. You have not mentioned whether you have made adjustments to several comparisons. The risk management of Type I errors is not clear.

Note: indicate whether you used multiplicity corrections. Otherwise, justify and distinguish between confirmatory and exploratory analysis.

Thank you for this important comment. We agree that the large number of comparisons requires careful consideration and transparent reporting.

All primary analyses were conducted within a Bayesian framework, in which multiplicity corrections are not required (e.g., 10.1080/19345747.2011.618213), as Bayes factors quantify the relative evidence for each model given the observed data, rather than relying on a repeated-sampling logic that necessitates control of Type I error rates (10.3758/s13423-017-1343-3). Nevertheless, we acknowledge that conducting multiple analyses increases the risk of overinterpretation. To further strengthen the interpretability of our findings and guard against over-interpretation, we now explicitly label analyses as confirmatory or exploratory in the Methods section.

“For state VoPE and cerebral oxygenation (oxygenated (HbO) and de-oxygenated (HbR), hemoglobin concentration) of the interval task, mean values of the second minute of the ride were analyzed using the same 2-between (condition: TG, CG) x 3-within (intensity levels: low, moderate, vigorous) x 2-within (time: pre- and post-training) Bayesian repeated measures ANOVA design in JASP. For the free ride task, 2-between (condition: TG, CG) x 2-within (time: pre- and post-training) Bayesian repeated measures ANOVAs were conducted for heart rate (10-minute mean), RPE, rendered power (10-minute mean, normalized to the RCP), and state VoPE. All

Attachment Submitted filename: Response_to_Reviewers_auresp_3.docx

# Peer review: Decision Letter 3

**DOI:** 10.1371/journal.pone.0352897.r007

**Authors:** Christopher Kirk, Christopher Kirk, Christopher Kirk, Christopher Kirk

21 May 2026

PONE-D-25-42417R3

An 8-week jump training did not boost effort value or the willingness to exert effort in a student sample

PLOS One

Dear Dr. Stähler,

Thank you for submitting your manuscript to PLOS ONE. After careful consideration, we feel that it has merit but does not fully meet PLOS ONE’s publication criteria as it currently stands. Therefore, we invite you to submit a revised version of the manuscript that addresses the points raised during the review process.

================================

The reviewers require one final point to be addressed:

Required Revisions (Minor)

1. fNIRS sensitivity analysis

The authors provided signal quality data (6.3% channel rejection) and re-processed the data with a different preprocessing pipeline that yielded consistent results. A point of interest, however, you are specifically asked for an analysis of the sensitivity of the results to tighter motion-correction parameters (such as tighter IQR limits). This query has to be rectified.

REVISE: Perform requested sensitivity analysis using more stringent parameters and report the results, or, if the sensitivity analysis is not needed, justify briefly in manuscript why this is not needed.

Please either complete and report these analyses, or provide a justification as to why such an analysis would not be needed in this instance.

================================

Please submit your revised manuscript by Jul 05 2026 11:59PM. If you will need more time than this to complete your revisions, please reply to this message or contact the journal office at plosone@plos.org. When you're ready to submit your revision, log on to https://www.editorialmanager.com/pone/ and select the 'Submissions Needing Revision' folder to locate your manuscript file.

Please include the following items when submitting your revised manuscript:

- A letter that responds to each point raised by the academic editor and reviewer(s). You should upload this letter as a separate file labeled 'Response to Reviewers'.
- A marked-up copy of your manuscript that highlights changes made to the original version. You should upload this as a separate file labeled 'Revised Manuscript with Track Changes'.
- An unmarked version of your revised paper without tracked changes. You should upload this as a separate file labeled 'Manuscript'.

If you would like to make changes to your financial disclosure, please include your updated statement in your cover letter. Guidelines for resubmitting your figure files are available below the reviewer comments at the end of this letter.

If applicable, we recommend that you deposit your laboratory protocols in protocols.io to enhance the reproducibility of your results. Protocols.io assigns your protocol its own identifier (DOI) so that it can be cited independently in the future. For instructions see: https://journals.plos.org/plosone/s/submission-guidelines#loc-laboratory-protocols. Additionally, PLOS ONE offers an option for publishing peer-reviewed Lab Protocol articles, which describe protocols hosted on protocols.io. Read more information on sharing protocols at https://plos.org/protocols?utm_medium=editorial-email&utm_source=authorletters&utm_campaign=protocols.

As the corresponding author, your ORCID iD is verified in the submission system and will appear in the published article. PLOS supports the use of ORCID, and we encourage all coauthors to register for an ORCID iD and use it as well. Please encourage your coauthors to verify their ORCID iD within the submission system before final acceptance, as unverified ORCID iDs will not appear in the published article. *Only*  the individual author can complete the verification step; PLOS staff *cannot*  verify ORCID iDs on behalf of authors.

We look forward to receiving your revised manuscript.

Kind regards,

Christopher Kirk, PhD, FCASES

Academic Editor

PLOS One

Journal Requirements:

1. If the reviewer comments include a recommendation to cite specific previously published works, please review and evaluate these publications to determine whether they are relevant and should be cited. There is no requirement to cite these works unless the editor has indicated otherwise.

2. Please review your reference list to ensure that it is complete and correct. If you have cited papers that have been retracted, please include the rationale for doing so in the manuscript text, or remove these references and replace them with relevant current references. Any changes to the reference list should be mentioned in the rebuttal letter that accompanies your revised manuscript. If you need to cite a retracted article, indicate the article’s retracted status in the References list and also include a citation and full reference for the retraction notice.

[Note: HTML markup is below. Please do not edit.]

Reviewers' comments:

Reviewer's Responses to Questions

**Comments to the Author**

1. If the authors have adequately addressed your comments raised in a previous round of review and you feel that this manuscript is now acceptable for publication, you may indicate that here to bypass the “Comments to the Author” section, enter your conflict of interest statement in the “Confidential to Editor” section, and submit your "Accept" recommendation.

Reviewer #3: All comments have been addressed

Reviewer #4: (No Response)

**********

2. Is the manuscript technically sound, and do the data support the conclusions?

The manuscript must describe a technically sound piece of scientific research with data that supports the conclusions. Experiments must have been conducted rigorously, with appropriate controls, replication, and sample sizes. The conclusions must be drawn appropriately based on the data presented.

Reviewer #3: Yes

Reviewer #4: Partly

**********

3. Has the statistical analysis been performed appropriately and rigorously?

Reviewer #3: Yes

Reviewer #4: No

**********

4. Have the authors made all data underlying the findings in their manuscript fully available?

The PLOS Data policy requires authors to make all data underlying the findings described in their manuscript fully available without restriction, with rare exception (please refer to the Data Availability Statement in the manuscript PDF file). The data should be provided as part of the manuscript or its supporting information, or deposited to a public repository. For example, in addition to summary statistics, the data points behind means, medians and variance measures should be available. If there are restrictions on publicly sharing data—e.g. participant privacy or use of data from a third party—those must be specified.

Reviewer #3: Yes

Reviewer #4: Yes

**********

5. Is the manuscript presented in an intelligible fashion and written in standard English?

PLOS ONE does not copyedit accepted manuscripts, so the language in submitted articles must be clear, correct, and unambiguous. Any typographical or grammatical errors should be corrected at revision, so please note any specific errors here.

Reviewer #3: Yes

Reviewer #4: Yes

**********

6. Review Comments to the Author

Please use the space provided to explain your answers to the questions above. You may also include additional comments for the author, including concerns about dual publication, research ethics, or publication ethics. (Please upload your review as an attachment if it exceeds 20,000 characters)

Reviewer #3: The article is much improved and clear.

I personally don't like paragraphs starting with 'however' (there are 2 in the introduction), It seems the idea is not complete. Besides that, no other recommendations.

Reviewer #4: Required Revisions (Minor)

1. fNIRS sensitivity analysis

The authors provided signal quality data (6.3% channel rejection) and re-processed the data with a different preprocessing pipeline that yielded consistent results. A point of interest, however, you are specifically asked for an analysis of the sensitivity of the results to tighter motion-correction parameters (such as tighter IQR limits). This query has to be rectified.

REVISE: Perform requested sensitivity analysis using more stringent parameters and report the results, or, if the sensitivity analysis is not needed, justify briefly in manuscript why this is not needed.

Addressed items – comments (For Author Information – No Further Action Needed)

2. Multiple comparisons – Sufficiently dealt with. The authors provided justification for the Bayesian approach and clearly differentiated between confirmatory vs. exploratory analyses.

3. Baseline transparency – Good; dealt with well. The baseline table was provided with detailed information including statistical tests and effect sizes. Means were calculated for each group with the baseline difference taken into account using ANCOVA.

The scope of boundary conditions / conclusion is addressed adequately. The final statement now truly addresses the shortcomings of the training paradigm, sample and measurement timing.

5.1 Problem with moderation analysis – 5.1.1 Limitation – addressed. A limitation paragraph was added, which included the absence of a subgroup analysis, small sample sizes, and recommendations for future studies (VO2max, previous activity and tolerance to effort).

**********

7. PLOS authors have the option to publish the peer review history of their article (what does this mean?). If published, this will include your full peer review and any attached files.

If you choose “no”, your identity will remain anonymous but your review may still be made public.

**Do you want your identity to be public for this peer review?**  For information about this choice, including consent withdrawal, please see our Privacy Policy.

Reviewer #3: No

Reviewer #4: No

**********

[NOTE: If reviewer comments were submitted as an attachment file, they will be attached to this email and accessible via the submission site. Please log into your account, locate the manuscript record, and check for the action link "View Attachments". If this link does not appear, there are no attachment files.]

To ensure your figures meet our technical requirements, please review our figure guidelines: https://journals.plos.org/plosone/s/figures

You may also use PLOS’s free figure tool, NAAS, to help you prepare publication quality figures: https://journals.plos.org/plosone/s/figures#loc-tools-for-figure-preparation.

NAAS will assess whether your figures meet our technical requirements by comparing each figure against our figure specifications.

# Author comment: Author response to Decision Letter 4

**DOI:** 10.1371/journal.pone.0352897.r008

12 Jun 2026

------------------------------------------------ comments Reviewer #3 ------------------------------------------

The article is much improved and clear.

I personally don't like paragraphs starting with 'however' (there are 2 in the introduction), It seems the idea is not complete. Besides that, no other recommendations.

Thank you for this helpful suggestion. We agree that beginning paragraphs with “however” can make the transition appear somewhat abrupt. We therefore replaced these paragraph openings with smoother transitional phrasing to improve the overall flow and readability of the manuscript.

“At the same time, accumulating evidence indicates that physical effort can simultaneously be perceived as valuable – at least to some degree, for some people, and in some situations (6,10).” (p. 6)

“This interpretation should be considered in light of the relatively low effort.” (p. 7)

------------------------------------------------ comments Reviewer #4 ------------------------------------------

Required Revisions (Minor)

1. fNIRS sensitivity analysis

The authors provided signal quality data (6.3% channel rejection) and re-processed the data with a different preprocessing pipeline that yielded consistent results. A point of interest, however, you are specifically asked for an analysis of the sensitivity of the results to tighter motion-correction parameters (such as tighter IQR limits). This query has to be rectified.

REVISE: Perform requested sensitivity analysis using more stringent parameters and report the results, or, if the sensitivity analysis is not needed, justify briefly in manuscript why this is not needed.

Thank you for this important comment regarding the sensitivity of the findings to motion-correction choices.

In the primary preprocessing pipeline, we already employed stringent motion-correction procedures for a high-movement exercise paradigm. Specifically, the wavelet-based correction used an IQR threshold of 1.0, which is stricter than the commonly used threshold of 1.5 (Gao et al., 2022; Molavi & Dumont, 2012). At the same time, excessively aggressive denoising (such as further decreasing the IQR) may attenuate meaningful hemodynamic signal and increase the risk of overcorrection, particularly in physically demanding movement tasks (Brigadoi et al., 2014; Chiarelli er al., 2015).

We therefore believe that further increasing correction strictness would be unlikely to provide meaningful benefits while increasing the risk of overcorrection.

Nevertheless, to further evaluate the robustness of the findings and assess whether the results were dependent on the specific preprocessing choices applied in the primary pipeline, we undertook the additional effort of reprocessing the entire dataset using an alternative preprocessing stream. For this we used an appropriate processing stream that has been employed in previous physical exercise research, as reported in our previous revision.

To enhance transparency for readers, we now explicitly report this supplementary analysis and explicitly note that the observed effects were consistent across preprocessing pipelines, indicating that the findings were not driven by specific motion-correction parameter choices.

“To assess the robustness of the findings with respect to preprocessing choices, the data were additionally processed using an alternative fNIRS preprocessing pipeline previously applied in physical effort paradigms (47). This alternative approach yielded highly similar results and did not change the overall pattern of findings, indicating that the reported results were not driven by specific preprocessing parameter choices.” (p. 19)

Attachment Submitted filename: Response_to_Reviewers_auresp_4.docx

# Editor report: Decision Letter 4

**DOI:** 10.1371/journal.pone.0352897.r009

**Authors:** Christopher Kirk, Christopher Kirk, Christopher Kirk, Christopher Kirk, Christopher Kirk

16 Jun 2026

An 8-week jump training did not boost effort value or the willingness to exert effort in a student sample

PONE-D-25-42417R4

Dear Dr. Stähler,

We’re pleased to inform you that your manuscript has been judged scientifically suitable for publication and will be formally accepted for publication once it meets all outstanding technical requirements.

Within one week, you’ll receive an e-mail detailing the required amendments. When these have been addressed, you’ll receive a formal acceptance letter and your manuscript will be scheduled for publication.

An invoice will be generated when your article is formally accepted. Please note, if your institution has a publishing partnership with PLOS and your article meets the relevant criteria, all or part of your publication costs will be covered. Please make sure your user information is up-to-date by logging into Editorial Manager at Editorial Manager® and clicking the ‘Update My Information' link at the top of the page. For questions related to billing, please contact billing support.

If your institution or institutions have a press office, please notify them about your upcoming paper to help maximize its impact. If they’ll be preparing press materials, please inform our press team as soon as possible -- no later than 48 hours after receiving the formal acceptance. Your manuscript will remain under strict press embargo until 2 pm Eastern Time on the date of publication. For more information, please contact onepress@plos.org.

Kind regards,

Christopher Kirk, PhD, FCASES

Academic Editor

PLOS One

Additional Editor Comments (optional):

Reviewers' comments:

# Editor report: Acceptance letter

**DOI:** 10.1371/journal.pone.0352897.r010

**Authors:** Christopher Kirk, Christopher Kirk, Christopher Kirk, Christopher Kirk, Christopher Kirk

PONE-D-25-42417R4

PLOS One

Dear Dr. Stähler,

I'm pleased to inform you that your manuscript has been deemed suitable for publication in PLOS One. Congratulations! Your manuscript is now being handed over to our production team.

At this stage, our production department will prepare your paper for publication. This includes ensuring the following:

* All references, tables, and figures are properly cited

* All relevant supporting information is included in the manuscript submission,

* There are no issues that prevent the paper from being properly typeset

You will receive further instructions from the production team, including instructions on how to review your proof when it is ready. Please keep in mind that we are working through a large volume of accepted articles, so please give us a few days to review your paper and let you know the next and final steps.

Lastly, if your institution or institutions have a press office, please let them know about your upcoming paper now to help maximize its impact. If they'll be preparing press materials, please inform our press team within the next 48 hours. Your manuscript will remain under strict press embargo until 2 pm Eastern Time on the date of publication. For more information, please contact onepress@plos.org.

You will receive an invoice from PLOS for your publication fee after your manuscript has reached the completed accept phase. If you receive an email requesting payment before acceptance or for any other service, this may be a phishing scheme. Learn how to identify phishing emails and protect your accounts at https://explore.plos.org/phishing.

If we can help with anything else, please email us at customercare@plos.org.

Thank you for submitting your work to PLOS ONE and supporting open access.

Kind regards,

PLOS ONE Editorial Office Staff

on behalf of

Dr. Christopher Kirk

Academic Editor

PLOS One
